# Characterization of a thermostable Cas13 enzyme for one-pot detection of SARS-CoV-2

**DOI:** 10.1073/pnas.2118260119

**Published:** 2022-06-28

**Authors:** Ahmed Mahas, Tin Marsic, Mauricio Lopez-Portillo Masson, Qiaochu Wang, Rashid Aman, Cheng Zheng, Zahir Ali, Madain Alsanea, Ahmed Al-Qahtani, Bernard Ghanem, Fatimah Alhamlan, Magdy Mahfouz

**Affiliations:** ^a^Laboratory for Genome Engineering and Synthetic Biology, Division of Biological Sciences, King Abdullah University of Science and Technology, Thuwal 23955-6900, Saudi Arabia;; ^b^Image and Video Understanding Laboratory, Computer, Electrical and Mathematical Sciences and Engineering, King Abdullah University of Science and Technology, Thuwal 23955-6900, Saudi Arabia;; ^c^Department of Infection and Immunity, King Faisal Specialist Hospital and Research Centre, Riyadh 11564, Saudi Arabia

**Keywords:** CRISPR Cas13, diagnostics, thermostable Cas13, CRISPR diagnostics, transcriptome editing

## Abstract

Type VI CRISPR-Cas systems have been repurposed for various applications such as gene knockdown, viral interference, and diagnostics. However, the identification and characterization of thermophilic orthologs will expand and unlock the potential of diverse biotechnological applications. Herein, we identified and characterized a thermostable ortholog of the Cas13a family from the thermophilic organism *Thermoclostridium caenicola* (TccCas13a). We show that TccCas13a has a close phylogenetic relation to the HheCas13a ortholog from the thermophilic bacterium *Herbinix hemicellulosilytica* and shares several properties such as thermostability and inability to process its own pre-CRISPR RNA. We demonstrate that TccCas13a possesses robust *cis* and *trans* activities at a broad temperature range of 37 to 70 °C, compared with HheCas13a, which has a more limited range and lower activity. We harnessed TccCas13a thermostability to develop a sensitive, robust, rapid, and one-pot assay, named OPTIMA-dx, for severe acute respiratory syndrome coronavirus 2 (SARS-CoV-2) detection. OPTIMA-dx exhibits no cross-reactivity with other viruses and a limit of detection of 10 copies/μL when using a synthetic SARS-CoV-2 genome. We used OPTIMA-dx for SARS-CoV-2 detection in clinical samples, and our assay showed 95% sensitivity and 100% specificity compared with qRT-PCR. Furthermore, we demonstrated that OPTIMA-dx is suitable for multiplexed detection and is compatible with the quick extraction protocol. OPTIMA-dx exhibits critical features that enable its use at point of care (POC). Therefore, we developed a mobile phone application to facilitate OPTIMA-dx data collection and sharing of patient sample results. This work demonstrates the power of CRISPR-Cas13 thermostable enzymes in enabling key applications in one-pot POC diagnostics and potentially in transcriptome engineering, editing, and therapies.

## Significance

Thermostable Cas13 orthologues will expand and unlock the power of CRISPR systems for synthetic biology and biotechnology applications. We identified and characterized a thermostable Cas13 orthologue from *Thermoclostridium caenicola* (TccCas13a), which exhibits robust *cis* and *trans* catalytic activities over a broad range of temperatures from 37 to 70 °C. To demonstrate the utility of the thermostable Cas13 enzymes in enabling biotechnological applications, we coupled reverse transcription–loop-mediated isothermal amplification to TccCas13a to develop a one-pot assay for virus detection. The developed assay exhibits key point-of-care (POC) features, enabling its use for the detection of different pathogens. This platform was clinically validated and is suitable for multiplexed detection. TccCas13a will enable advancements in POC diagnostics, transcriptome engineering, editing, and targeted therapies.

CRISPR-Cas systems endow bacterial and archaeal species with adaptive molecular immunity to help fend off invading phages and plasmids via RNA-guided nucleases (1–3). CRISPR-Cas systems acquire a molecular record, a spacer, from the invader’s genome, transcribe and process the CRISPR array containing the spacer, and use a Cas endonuclease to interfere with the invader and target its genome for degradation (4, 5). Class II CRISPR systems use a single effector RNA-guided nuclease for interference. Due to their facile engineering, they have been harnessed in diverse applications, including genome engineering, gene knockdown, virus interference, bioimaging, and diagnostics (1, 6–8).

Type VI CRISPR-Cas systems target single-stranded RNA (ssRNA), with six subtypes identified (subtypes VI A–D, X, and Y) and five subtypes functionally characterized (types VI A, B, D, X, and Y) with their signature effectors Cas13a, Cas13b, Cas13d, Cas13X, and Cas13Y, respectively (9–11). Intriguingly, Cas13 enzymes possess two distinct but interdependent ribonuclease (RNase) activities (12). Type VI CRISPR-Cas13 systems have an RNA-guided RNase domain; once activated, this domain induces promiscuous collateral degradation of nearby ssRNA molecules (13). Indeed, Cas13 enzymes have been employed for various CRISPR-based detection applications (14–18). These CRISPR-based modalities have paved the way for developing the next generation of diagnostics by exploiting highly specific target recognition and cleavage by Cas enzymes, followed by cleavage of reporters in *trans* by collateral activity (19–22). The unprecedented versatility, programmability, and simplicity of CRISPR-based diagnostics without the need for sophisticated instruments render them amenable to the development of point-of-care (POC) diagnostics (23–26).

Characterizing Cas13 enzymes from thermophiles is critical to expand the molecular toolbox of RNA engineering and unlock the potential of biotechnological applications, including molecular diagnostics. Indeed, as the discovery of a thermostable DNA polymerase from *Thermus aquaticus* opened up remarkable possibilities for new assays (27), the discovery of Cas13 enzymes with broader operating temperatures will enable the development of assays that require fewer steps, thus limiting opportunities for contamination and making these easy to use and implement in developing single-pot closed-system devices for POC diagnostics.

Advances in synthetic biology and bioengineering have spurred a wave of innovations in molecular diagnostics (28). To increase their sensitivity, CRISPR-based systems have been coupled to isothermal amplification methods such as recombinase polymerase amplification (RPA) or loop-mediated isothermal amplification (LAMP), enabling target nucleic acid detection at attomolar (10^−18^ M) levels (16, 18, 29). Cas13 systems coupled with reverse transcription (RT)-RPA or RT-LAMP have been employed for severe acute respiratory syndrome coronavirus 2 (SARS-CoV-2) detection with several optimizations to enhance their sensitivity and specificity (30–32). LAMP has arisen as the method of choice for sensitive and specific detection due to its high sensitivity, rapid turnaround time, simple operation, and low cost (33). However, current CRISPR systems use Cas nucleases isolated from mesophilic bacteria and operating best at ∼37 °C, while LAMP systems operate at higher temperatures (∼55 to 65 °C). Therefore, RT-RPA– and RT-LAMP–coupled CRISPR detection assays are mostly conducted in two pots or tubes.

Two-pot assays suffer from cross-contamination, as opening the first tube can create aerosols, necessitating the two reactions be conducted in physically separate areas. In addition, recently developed one-pot assays rely on lateral flow readouts that require opening the reaction tube postamplification, increasing the chance of cross-contamination (34). Therefore, current work focuses on developing single-pot, single-temperature assays to limit cross-contamination, facilitate end-user handling, enable POC applications, and develop at-home testing kits.

Identification of thermostable CRISPR-Cas enzymes will enable their simultaneous application with RT-LAMP in a single tube. In this work, we report the identification and characterization of a thermophilic Cas13a ortholog from *Thermoclostridium caenicola* (TccCas13a) that is highly active at the temperatures required for RT-LAMP. TccCas13a exhibits robust *cis* and *trans* catalytic activities at a broad range of temperatures (37 to 70 °C). Interestingly, TccCas13a does not process its own pre–CRISPR RNA (crRNA). We coupled this thermophilic Cas13a variant with RT-LAMP for virus detection in a one-pot assay, which we named OPTIMA-dx. In addition, by coupling the specific *cis* and *trans* cleavage preferences of TccCas13a with another thermostable Cas enzyme (AapCas12b), we developed OPTIMA-dx for multiplexed detection of more than one target in the same reaction. We validated our one-pot detection module for SARS-CoV-2 detection in clinical samples from patients with COVID-19. Furthermore, we developed a mobile phone application using a machine-learning approach to streamline the reading and collection of test results and allow data sharing with central health care systems. These fundamental advances will help develop an effective and low-cost POC modality for massive-scale testing that detects SARS-CoV-2 and will be useful for future tests for other pathogens. The characterization of thermophilic Cas13 enzymes will expand the molecular toolbox for RNA engineering and editing and enable diverse biotechnological applications, including sensitive and specific POC diagnostics.

## Results

### Identification of Thermophilic Cas13 Proteins.

**Fig. 1.**
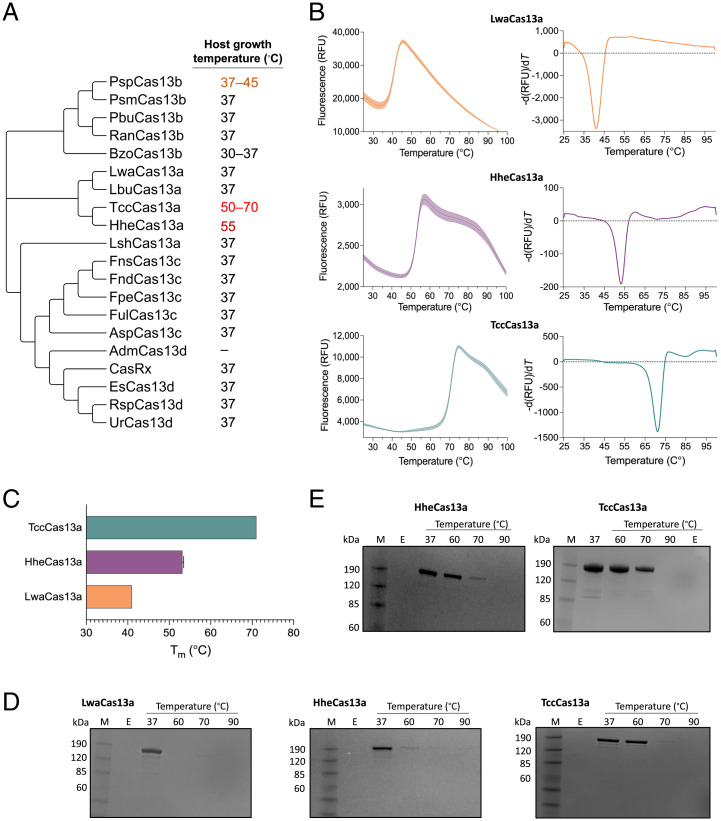
Thermostability analysis of thermophilic Cas13 effectors. (*A*) Maximum-likelihood phylogenetic tree of Cas13 proteins from different organisms. The tree was generated using MEGA X software. Most selected proteins were isolated from mesophilic bacteria, although several have been cultivated as thermophiles and thus offer an interesting collection of high temperature–stable proteins. TccCas13a and HheCas13a were selected as potentially thermophilic Cas13 proteins. (*B*) Differential scanning fluorimetry (DSF) profiles of protein melting points using a conventional real-time PCR instrument. The peak in the left graph indicates protein denaturation. The right-side graph is the derivative of the left-side graph. (*C*) Denaturation temperatures of TccCas13a, HheCas13a, and LwaCas13a proteins, as determined by DSF in *B*. Data are shown as mean ± SD (SD) (*n* = 3). (*D*) SDS-PAGE illustrating the protein stability of TccCas13a, HheCas13a, and LwaCas13a after incubation for 30 min at different temperatures. M, protein marker; E, empty well. (*E*) Sodium dodecyl sulfate–polyacrylamide gel electrophoresis (SDS-PAGE) showing the protein stability of TccCas13a and HheCas13a RNPs assembled with their cognate crRNAs. After assembly with crRNAs, TccCas13a and HheCas13a RNPs were incubated for 30 min at different temperatures before electrophoresis. RFU, relative fluorescence unit.

Here, we set out to identify Cas13 proteins from thermophilic bacteria. We interrogated existing Cas13 variants to determine whether they originated from thermophilic hosts. We thus identified HheCas13a from the thermophilic bacterium *Herbinix hemicellulosilytica* as a potential thermophilic protein (35, 36). Subsequently, we used the HheCas13a protein sequence as a query in a BLAST-P search against nonredundant protein databases, leading to the isolation of another likely thermophilic Cas13a homolog from *T*. *caenicola* (i.e., TccCas13a) (Fig. 1*A*).

We synthesized the gene encoding TccCas13a and used the available clone of HheCas13a for heterologous production in *Escherichia coli* and purified the recombinant proteins to homogeneity. Subsequently, we conducted differential scanning fluorimetry to test their thermostability. Both proteins possessed a denaturation temperature higher than Cas13 enzymes from mesophilic bacteria, e.g., LwaCas13a (Fig. 1 *B* and *C*). We next reasoned that complexing the crRNA to the HheCas13a and TccCas13a proteins would further stabilize the proteins at higher temperatures. We performed an in silico search for the TccCas13a crRNA direct-repeat sequence using CRISPRCasFinder software. We identified the TccCas13a-associated CRISPR array and designed the TccCas13a crRNAs based on this in silico prediction (*SI Appendix*, Fig. S1). For HheCas13a, we based the crRNA design on the previously reported sequence (36). We incubated TccCas13a and HheCas13a with and without their respective in vitro–transcribed crRNAs at different temperatures and separated the complexes by sodium dodecyl sulfate–polyacrylamide gel electrophoresis. Indeed, TccCas13a and HheCas13a loaded with their cognate crRNAs exhibited higher thermostability, with TccCas13a showing greater stability than HheCas13a (Fig. 1 *D* and *E*). We concluded that both proteins exhibit the needed thermostability for our projected downstream applications requiring *cis* and *trans* catalytic activities at higher temperatures.

### Characterization of the *Cis* and *Trans* Catalytic Activities of TccCas13a and HheCas13a.

**Fig. 2.**
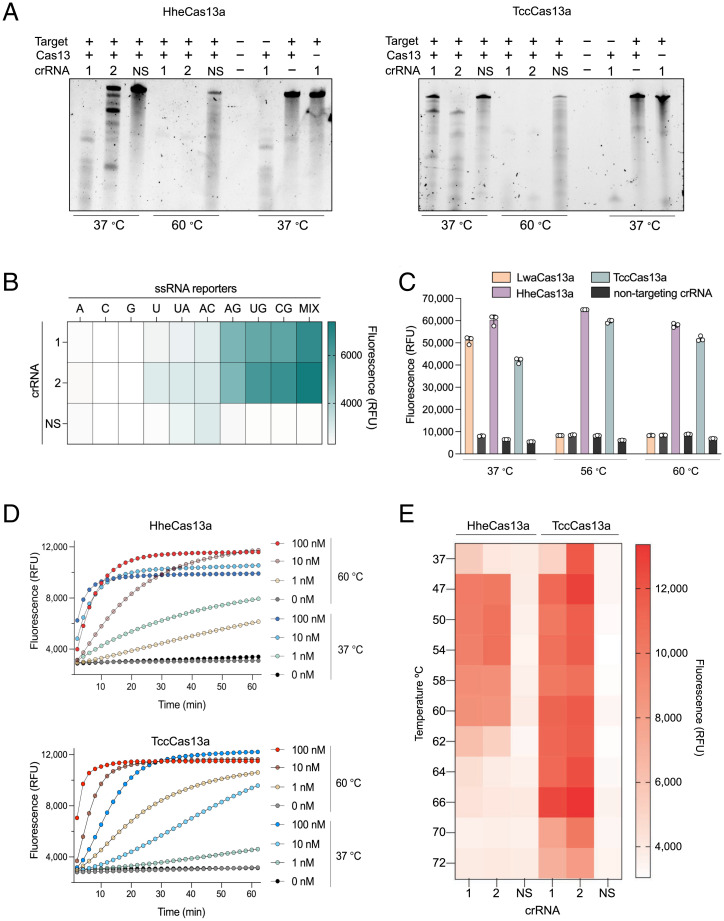
Characterization of *cis* and *trans* cleavage activities of thermophilic HheCas13a and TccCas13a. (*A*) Representative denaturing gels showing the targeted in vitro RNase cleavage activity of HheCas13a and TccCas13a proteins when incubated with ssRNA targets and different crRNAs at different temperatures. NS, nonspecific crRNA control. (*B*) TccCas13a collateral cleavage preference for the ssRNA reporter. Reactions consisting of TccCas13a and its respective cognate crRNAs or NS were performed in the presence of ssRNA targets and one of 10 ssRNA reporters. Data are shown as mean (*n* = 3). Reactions were incubated at 56 °C, and the end-point fluorescent signal was measured after 30 min. ssRNA reporter sequences are shown on top of the heat map. A, poly A reporter; C, poly C reporter; G, poly G reporter; U, poly U reporter; UA, LwaCas13a reporter; AC, 3(AC) reporter; AG, 3(AG) reporter; UG, 3(UG) reporter; CG, 3(CG) reporter; Mix, mix reporter (UGACGU) (*SI Appendix*, Table S6). (*C*) End-point activity of LwaCas13a, HheCas13a, and TccCas13a at different temperatures using their preferred reporter (*SI Appendix*, Table S6 for LwaCas13a reporter). One crRNA and a nonspecific crRNA were tested for each. Values are shown as mean ± SD and represent end-point fluorescence at 30 min. (*D*) Measurement of real-time fluorescence output comparing the detection activity of HheCas13a and TccCas13a at three different target RNA concentrations (100, 10, and 1 nM) and two temperatures (37 and 60 °C). Data are shown as mean (*n* = 3). (*E*) End-point activity of HheCas13a and TccCas13a at different temperatures using their preferred reporter after 30 min. Two crRNAs and a nonspecific crRNA were tested for each. Data are shown as mean (*n* = 3). RFU, relative fluorescence unit.

Our initial characterization demonstrated that both proteins remained folded at higher temperatures and that loading of the crRNA enhanced their thermostability. We next tested whether their respective ribonucleoprotein (RNP) complexes are active and mediate the *cis* and *trans* activities essential for downstream applications. Accordingly, we designed crRNAs targeting a synthetic RNA sequence to determine whether either protein exhibited catalytic *cis* activities at elevated temperatures. Both HheCas13a and TccCas13a proteins did show robust and higher *cis* catalytic activities at higher temperatures (60 °C) when incubated with their target RNA than when incubated at 37 °C (Fig. 2*A*).

Next, we attempted to determine whether HheCas13a and TccCas13a retained nonspecific *trans* degradation activity of ssRNA reporter molecules in the presence of ssRNA target at elevated temperatures. Such collateral activity is critical for diagnostics, as it is the basis for nucleic acid detection and other applications of CRISPR-Cas13 systems. Different Cas13 variants trigger collateral RNase activity with distinct cleavage preferences depending on the ssRNA sequences (18). Therefore, we screened several ssRNA reporters consisting of 6 mers of A, U, C, or G homopolymers; reporters with dinucleotide motifs including UG, CG, or AC; or a 6-mer probe with a mix of U, G, A, and C nucleotides. This reporter-screening assay showed a preference of TccCas13a collateral cleavage activity for reporters containing GH dinucleotide motifs, but not G homopolymers. However, TccCas13a exhibited robust *trans* cleavage and a strong preference for the mixed nucleotide reporter (Fig. 2*B*). HheCas13a, however, displayed robust *trans* cleavage using the poly U reporter, consistent with a recent study (*SI Appendix*, Fig. S3) (36).

We next demonstrated the thermostability of HheCas13a and TccCas13a by testing the *trans* cleavage activity of HheCas13a and TccCas13a in comparison to LwaCas13a at different temperatures. We incubated LwaCas13a, HheCas13a, and TccCas13a proteins with their cognate crRNAs at different temperatures for 25 min before the addition of the target RNA and their preferred ssRNA reporter molecules. Although LwaCas13a exhibited a very robust and fast signal when incubated at 37 °C, no activity was observed at high temperatures (Fig. 2*C*). In contrast, both HheCas13a and TccCas13a maintained robust *trans* cleavage activity at 56 and 60 °C (Fig. 2*C*). Moreover, we compared the *trans* cleavage activities of HheCas13a and TccCas13a on ssRNA reporters at 37 and 60 °C with matching crRNA spacer sequences. Both enzymes performed well at both temperatures, but TccCas13a showed faster and stronger *trans* cleavage activity at high temperatures and lower target RNA concentration than HheCas13a (Fig. 2*D*). Subsequently, we conducted RNA detection assays over a range of temperatures from 37 to 72 °C to determine the optimal temperature for TccCas13a and HheCas13a. TccCas13a was active over a wider range of temperatures (37 to 70 °C), while HheCas13a exhibited robust activity over the more limited temperature range of 37 to 60 °C (Fig. 2*E*). These data provide compelling evidence of the thermostability and robust catalytic activities of TccCas13a and HheCas13a proteins and their usefulness in applications requiring *cis* and *trans* catalytic activities.

### Biochemical Characterization of Thermostable TccCas13a and HheCas13a.

**Fig. 3.**
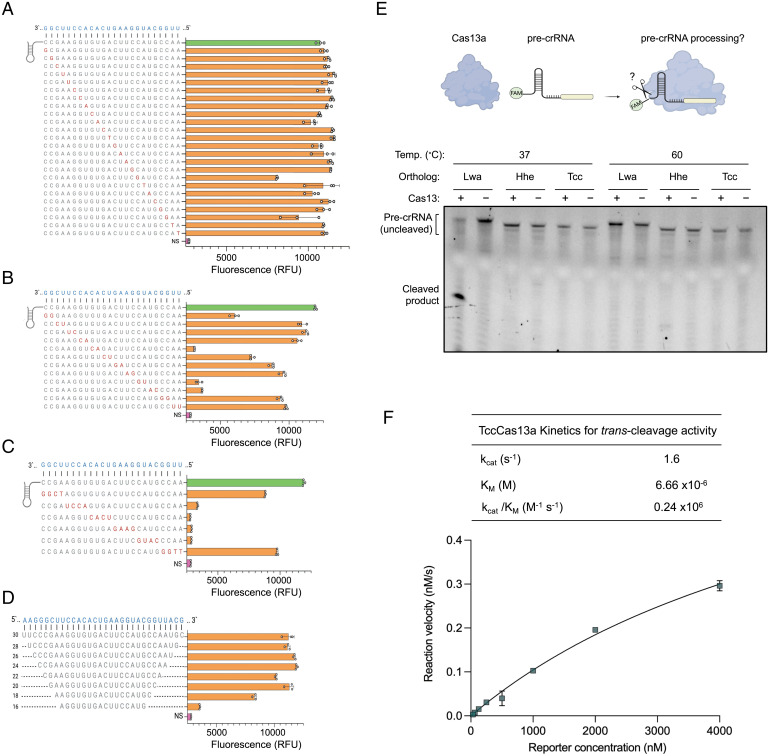
In vitro characterization of thermostable Cas13a crRNA sequence requirements. Evaluating the effect of single (*A*), double (*B*), and stretches of four mismatches (*C*) between crRNA and target RNA on TccCas13a activity. (*Left*) crRNA nucleotide sequence with the positions of mismatches (red) on the crRNA spacer. (*Right*) The fluorescence intensity, relative to the nonspecific crRNA control (NS) (pink) or crRNA with no mismatches (green), resulting from TccCas13a collateral cleavage activity on each tested crRNA. Reactions were incubated at 56 °C, and the end-point fluorescence signal was measured after 30 min. (*D*) TccCas13a RNA detection activity with different crRNA spacer lengths. (*Left*) crRNA spacer nucleotide sequences with the length of each spacer shown on the left of the sequence. (*Right*) The fluorescence intensity, relative to the NS (pink), resulting from TccCas13a collateral cleavage activity on each tested crRNA. Reactions were incubated at 56 °C, and the end-point fluorescence signal was measured after 30 min. (*E*) Representative denaturing gels showing Cas13a-mediated processing of their cognate precrRNAs; 100 nM each Cas13a ortholog was incubated with 200 nM cognate 5′-FAM–labeled precrRNA for 1 h at different temperatures. See *SI Appendix*, Fig. S5*A* for uncropped gel picture. (*F*) Representative Michaelis-Menten plot for TccCas13a-catalyzed ssRNA *trans* cleavage activity. Enzyme kinetic data and the measured K_cat_, K_m_, and K_cat/_K_m_ are shown on the top of the plot. Values are shown as mean ± SD (*n* = 3). RFU, relative fluorescence unit.

To further characterize the activity of the identified thermostable Cas13 proteins, we sought to test the crRNA requirements by introducing various modifications to the crRNA spacer sequences. We first tested the effect of single mismatches between crRNA and target RNA on HheCas13a and TccCas13a RNA detection activities. We mutated single bases across the crRNA spacer sequence to the respective complementary bases. We found that both HheCas13a and TccCas13a were tolerant to single mismatches across the spacer, as such mismatched spacers enabled RNA detection with similar efficiency as fully matched spacers (Fig. 3*A* and *SI Appendix*, Fig. S4*A*). However, double mismatches resulted in different tolerances for different regions of the spacer sequence with both HheCas13a and TccCas13a proteins (Fig. 3*B* and *SI Appendix*, Fig. S4*B*). In addition, when we introduced stretches of four mismatches in the spacer, we found a significant reduction in the activity of both Cas13 enzymes for mismatch stretches in the center of the spacer, while four consecutive mismatches at the extreme 5 or 3′ ends of the crRNA spacer had less of an effect, suggesting the presence of a seed region in the center of the spacer sequence, similar to previous observations with other Cas13 effectors (Fig. 3*C* and *SI Appendix*, Fig. S4*C*) (13). Next, we sought to determine the minimal spacer length required for efficient Cas13-mediated RNA detection. Therefore, we used a series of spacer truncations ranging in length from 30 to 16 nt. While HheCas13a required spacers 24 nt or longer to maintain efficient RNA detection activity (*SI Appendix*, Fig. S4*D*), TccCas13a exhibited robust activity with spacer sequences as short as 20 nt (Fig. 3*D*).

Notably, previous studies have shown the ability of CRISPR-Cas13 systems to process precrRNAs and generate mature crRNAs capable of guiding Cas13 enzymes to the target RNA (12). Interestingly, HheCas13a is the only known Cas13 ortholog that is incapable of processing precrRNAs, at least in vitro (36). Considering that both HheCas13a and TccCas13a are thermostable proteins and evolutionarily closely related (Fig. 1*A*), we wondered if TccCas13a is capable of processing precrRNAs in vitro. Therefore, using 5′-FAM–labeled precrRNA sequences, we tested the precrRNA processing activity of TccCas13a in comparison to HheCas13a and LwaCas13a proteins. LwaCas13a exhibited robust precrRNA processing activity, but HheCas13a did not process the cognate precrRNA when it was incubated at 37 or 60 °C, consistent with previously reported results (Fig. 3*E* and *SI Appendix*, Fig. S5) (36). To our surprise, we also did not detect any precrRNA processing activity with TccCas13a at the tested temperatures (Fig. 3*E* and *SI Appendix*, Fig. S5). These results, together with previous findings (36), indicate that the thermostable HheCas13a and TccCas13a enzymes are the only precrRNA processing–defective Cas13 homologs known to date, pointing to a possible relationship between the thermostability of these Cas13 variants and the lack of precrRNA processing activities.

The robust activity of TccCas13a observed in the previous experiments led us to further study the enzyme kinetics. Therefore, we performed Michaelis-Menten kinetic measurements of TccCas13a ssRNA *trans* cleavage activity at elevated temperatures and found that when TccCas13a was activated with ssRNA targets, it catalyzed *trans* ssRNA cleavage with catalytic efficiency (k_cat_/K_m_) of 0.24 × 10^6^ M^−1^ s^−1^ (Fig. 3*F* and *SI Appendix*, Fig. S6). The observed high value of K_m_ indicates the low affinity of the Cas13 enzyme with the nonspecific, in trans RNA target. Similar observations of high K_m_ values were recently reported with other Cas13 variants (37).

### RT-LAMP–Coupled Thermophilic CRISPR-Cas13a Enzymes for SARS-CoV-2 Detection.

**Fig. 4.**
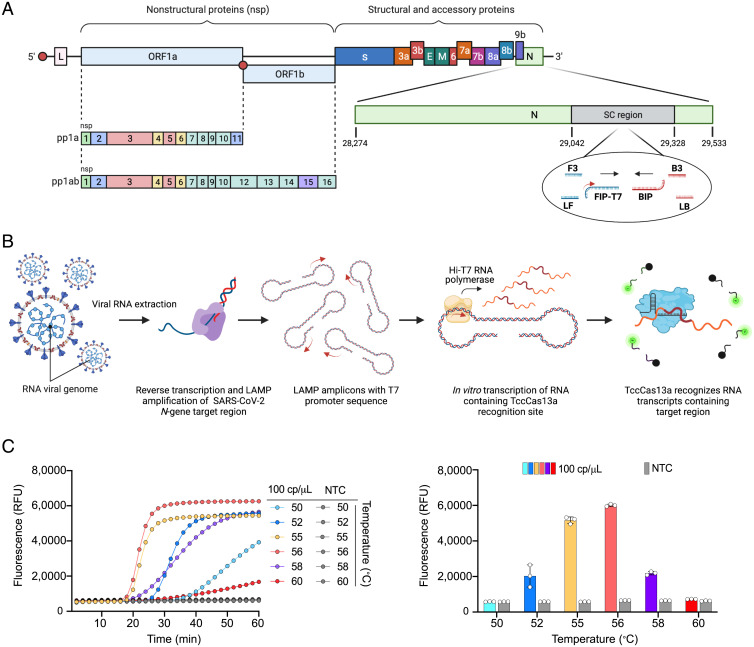
Establishment and optimization of one-pot SARS-CoV-2 detection using the thermophilic TccCas13a protein. (*A*) Schematic representation of the SARS-CoV-2 genome showing the region targeted by RT-LAMP amplification and the crRNA target sequence. The small red arrow on the T7-FIP primer indicates the T7 promoter sequence. SC region: genomic region of SARS-CoV-2 *N* gene targeted with STOPCovid primers. (*B*) Overview of the assay workflow. The detection protocol consists of three distinct steps, all carried out in the same tube and at the same temperature (56 °C). Following the extraction of viral RNA, specific target sequences within the viral RNA undergo RT into complementary DNA and are amplified with RT-LAMP isothermal amplification using LAMP primers containing the T7 promoter sequence (small red arrow on the T7-FIP primer). The resulting RT-LAMP amplicons are used for in vitro transcription using the thermostable Hi-T7 RNA polymerase, producing RNA transcripts that are recognized and targeted simultaneously by the thermophilic TccCas13a protein. Recognition of the RNA transcripts by TccCas13a triggers Cas13 collateral cleavage activity, resulting in trans cleavage of the reporter probe conjugated to the HEX or FAM fluorophores. (*C*) Performance of one-pot detection assay at different temperatures. (*Left*) As determined by real-time fluorescence at a given target RNA concentration (100 cp/μL); data are shown as mean (*n* = 3). (*Right*) End-point fluorescence signal measured after 30 min; values are shown as mean ± SD. The best performance was achieved at 56 °C. NTC, no template control; RFU, relative fluorescence unit.

Based on the results above, and considering the wide and increasingly growing applications of CRISPR-Cas13 for diagnostics, we recognized the possibility of developing a sensitive assay by coupling RT-LAMP isothermal amplification with in vitro transcription and subsequent Cas13-based detection in one step at the same temperature by using HheCas13a and/or TccCas13a. Two rounds of amplification of the target virus or genome can be conducted: the first round via RT-LAMP and the second via the in vitro transcription of the RT-LAMP products with T7 RNA polymerase. We first tested the applicability of this modality using TccCas13a and HheCas13a in a two-pot RT-LAMP assay. To ensure sensitive detection, preamplifying the RNA target of interest is a necessary step (16). We chose isothermal amplification with RT-LAMP and used well-established primer sets from previous reports to target and amplify conserved regions in the SARS-CoV-2 *N* gene, referred to here as STOPCovid primers (34). However, because Cas13 proteins target RNA, we modified these primers by appending a T7 promoter sequence to the 5′ end of the first half of either the forward inner primer (FIP) or the backward inner primer (BIP) (Fig. 4*A*). Therefore, during LAMP, the T7 promoter sequence should be incorporated into the amplified DNA products, providing a suitable template for the T7 RNA polymerase to transcribe the amplified LAMP product in vitro and generate RNA targets for Cas13 detection (Fig. 4*B*).

We designed several crRNAs for both TccCas13a and HheCas13a, targeting a highly conserved region in the SARS-CoV-2 *N* gene. We first showed that both HheCas13a and TccCas13a exhibit robust activity in RT-LAMP buffer (isothermal buffer) (*SI Appendix*, Fig. S2). We then performed an initial screening of these crRNAs and primers in a two-pot setting, as described above. Most of the tested crRNAs targeting the RNA transcript produced from LAMP products harboring the T7 promoter sequence from FIP-T7 or BIP-T7 primers showed robust performance and a high detection signal. However, crRNAs targeting regions of LAMP amplicons that are not transcribed showed no activity (*SI Appendix*, Fig. S7). These results support the strong detection of RT-LAMP products when using crRNAs, confirming that 1) the amplification of the synthetic SARS-CoV-2 genome was successful with the modified primers, 2) the T7 promoter was successfully integrated into the amplified products, and 3) the T7 RNA polymerase could use the amplified amplicons to generate Cas13a substrates that activate Cas13a enzymes to degrade ssRNA reporters and generate signal output (Fig. 4*B* and *SI Appendix*, Fig. S7).

### Establishment and Optimization of a One-Pot RT-LAMP with TccCas13a for SARS-CoV-2 Detection.

Both thermophilic Cas13a enzymes exhibited a practical and robust catalytic activity at higher temperatures in a two-pot assay, which motivated us to capitalize on their thermostability to establish a one-pot RT-LAMP–coupled CRISPR assay for the detection of SARS-CoV-2. We first investigated if target detection in a one-pot assay would be feasible at a single temperature. To this end, we rescreened all crRNAs and primer sets in one-pot settings, leading to the identification of a combination of crRNA and primer set with the most specific and efficient detection of SARS-CoV-2 RNA, namely TccCas13a crRNA#13, when used with the T7-FIP modified primer set (*SI Appendix*, Fig. S8). Interestingly, although HheCas13a exhibited a strong detection signal in two-pot settings, we observed no significant detection signal in one-pot settings with any crRNA. By contrast, TccCas13a was consistent in specifically and efficiently detecting SARS-CoV-2 target in one pot with the optimized combination of primers and crRNA (*SI Appendix*, Fig. S9). Therefore, we selected TccCas13a for further optimization and development of one-pot SARS-CoV-2 detection.

We set out to further optimize all reagents to improve the performance, sensitivity, and specificity of the assay for SARS-CoV-2 detection. We first proceeded to optimize the reaction chemistry by testing several *Bst* DNA polymerases. We noticed that optimal sensitivity and efficiency of our one-pot assay could only be achieved with certain commercially available *Bst* DNA polymerases (*SI Appendix*, Fig. S10), which might be a reflection of different buffer compositions and particular salt concentrations that would negatively affect Hi-T7 RNA polymerase activity. We further improved the reaction performance by titrating the *Bst* DNA polymerase, Hi-T7 RNA polymerase, Mg^2+^, and TccCas13a RNP concentrations in the reaction (*SI Appendix*, Fig. S11).

Because different biochemical reactions perform optimally at different temperatures in our one-pot assay, we tested the performance of the one-pot assay at different temperatures. The optimal temperature for the one-pot detection assay was 56 °C, with diminished performance at higher or lower temperatures, probably due to the reduced performance of LAMP at lower temperatures and of the Hi-T7 RNA polymerase at higher temperatures (Fig. 4*C*).

### Evaluation and Clinical Validation of OPTIMA-dx Assay for SARS-CoV-2 Visual Detection.

**Fig. 5.**
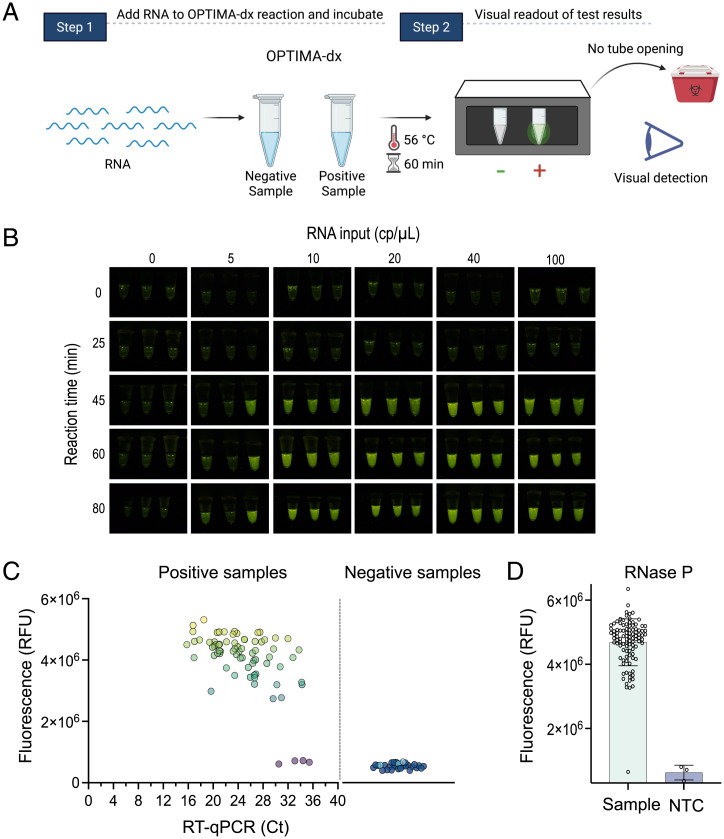
Evaluation of OPTIMA-dx for the detection of SARS-CoV-2. (*A*) Schematic representation of SARS-CoV-2 RNA detection in one-pot assays and visual detection using the P51 Molecular Fluorescence Viewer. As the test is performed in a single pot, there is no need to open the reaction tube, so it can be discarded without opening, thus avoiding the possibility of contamination at the POC site. (*B*) Assessment of the sensitivity of OPTIMA-dx and the effect of reaction incubation time on performance using fluorescence-based visual detection. Fluorescence rises above background after 45 min, with little improvement as time increases. Three replicates were performed for each treatment. (*C*) SARS-CoV-2 detection from 100 clinical COVID-19 samples with one-pot RT-LAMP TccCas13a detection assay. qRT-PCR Ct plotted against fluorescence readout from the detection of SARS-CoV-2–positive samples (*n* = 73) and SARS-CoV-2–negative samples (*n* = 27). Detection reactions were incubated at 56 °C, and the end-point fluorescence signal was measured after 1 h. Light-blue data points in negative samples represent no template controls (NTC). (*D*) Detection of RNase P internal control with one-pot RT-LAMP TccCas13a detection assay. All 100 clinical samples in *A* were tested for the detection of the RNase P gene. Detection reactions were incubated at 56 °C, and the end-point fluorescence signal was measured after 1 h. RFU, relative fluorescence unit.

To enable large-scale screening during a pandemic, performing diagnostic assays at POC or outside of laboratory settings is critical. Since the use of sophisticated fluorescence detection instruments such as qPCR machines or plate readers complicates the achievement of this goal, CRISPR diagnostic approaches have adapted lateral flow detection in an effort to develop a simple visual readout that can expedite accurate diagnostics in POC settings (18). However, despite the efficiency and simplicity of this approach, the reaction tubes need to be opened for lateral flow detection readouts, thus increasing the chance of aerosols and cross-contamination. As an alternative, we sought to couple our assay with a portable device. Using RNA reporter molecules conjugated to 5′ HEX or FAM fluorophores at the appropriate concentration, TccCas13a collateral cleavage produced a bright signal visible with a hand-held, inexpensive fluorescence visualizer (P51 Molecular Fluorescence Viewer), allowing simple visualization and interpretation of the results (Fig. 5*A*). We termed this one-pot assay with visual detection OPTIMA-dx (one-pot thermophilic Cas13 and isothermal amplification module for nucleic acid detection).

With these optimized reaction conditions, we next evaluated the analytical limit of detection (LoD) of OPTIMA-dx using synthetic SARS-CoV-2 RNA as input. We estimated the LoD of OPTIMA-dx assay to be 10 copies (cp)/μL, which can be achieved within 45 to 60 min of reaction time (Fig. 5*B*). To test specificity and absence of cross-reactivity, we challenged OPTIMA-dx with other common human viruses, including SARS-CoV-1, MERS-CoV, H1N1, HCoV-OC43, HCoV-229E, and HCoV-NL63. OPTIMA-dx showed high specificity to SARS-CoV-2, with no cross-reactivity against any of the other tested viruses (*SI Appendix*, Fig. S12*A*). We next assessed how storage at common storage temperatures influenced the performance of a preassembled OPTIMA-dx master mix. Although the OPTIMA-dx reaction did lose activity after storage for 48 h at 4 °C, the detection reaction remained active when stored at −20 °C for at least 10 d and after multiple freeze-thaw cycles (*SI Appendix*, Fig. S12*B*).

To ensure reliability of SARS-CoV-2 detection kits, the US Food and Drug Administration (FDA) guidelines (catalog #2019-nCoVEUA-01; Centers for Disease Control and Prevention [CDC], 2019) emphasize the importance of including a positive sample, or internal control, as an indicator of proper sample handling, RNA extraction, template quality and integrity, and validity of reagents. In particular, a negative SARS-CoV-2 readout should be considered invalid if the internal control is negative as well. We thus tested human RNase P transcripts as an internal control for the OPTIMA-dx SARS-CoV-2 detection assay, whereby each sample can be evaluated by two OPTIMA-dx reactions for the detection of SARS-CoV-2 and the RNase P internal control (*SI Appendix*, Fig. S12*C*). Accordingly, we designed two crRNAs targeting a region of RNase P amplified with RT-LAMP primers developed in previous reports that we modified with the T7 promoter sequence appended to the FIP primer (38). Both crRNAs showed efficient and specific detection, with crRNA 1 showing a faster detection signal compared with crRNA 2, prompting us to select crRNA 1 for the OPTIMA-dx RNase P assay (*SI Appendix*, Fig. S12*D*).

Next, we sought to validate the performance of our one-pot detection assay using RNA isolated from patient samples. We performed SARS-CoV-2 detection at King Faisal Specialist Hospital and Research Centre using 73 qRT-PCR–positive samples with a broad range of cycle threshold (Ct) values and different strains of SARS-CoV-2 and 27 qRT-PCR–negative samples extracted following the protocol approved by the CDC Emergency Use Authorization. Our one-pot SARS-CoV-2 detection assay demonstrated 94.5% sensitivity and 100% specificity, showing high concordance with the qRT-PCR data (Fig. 5*C*). Interestingly, although all four samples showing false-negative results had Ct values above 30, the detection assay was able to detect other samples with Ct values as high as 34, indicating the high sensitivity of our assay (Fig. 5*C*). We also tested all clinical samples for the *RNase P* gene and found that OPTIMA-dx detected the RNase P internal control in all tested samples, except one of the negative samples (Fig. 5*D*).

In addition, we validated OPTIMA-dx visual detection of SARS-CoV-2 with total RNA extracted from another set of swab samples collected from patients suspected to have SARS-CoV-2. We conducted our validation assays of OPTIMA-dx using RNA extracted from 45 randomized samples (different from samples used in Fig. 5*C*): 40 positive samples with Ct values ranging between 14 and 34, and five negative samples. We detected a positive OPTIMA-dx signal with all tested samples, with the exception of the negative samples, and no template control within 1 h (*SI Appendix*, Fig. S13). However, we noted that samples with Ct values above 30 showed a weaker signal compared with samples with Ct values below 30 (*SI Appendix*, Fig. S13). These results indicate that OPTIMA-dx can reliably detect SARS-CoV-2 in patient samples within 1 h, with a simple visual readout, for Ct values up to 34.

### Development and Assessment of Simple Extraction Method for POC Sample Processing.

An important consideration for rapid and simple POC diagnostics is to avoid complicated nucleic acid extraction procedures and kits that are labor intensive and costly and require specialized laboratory equipment and personnel training. Therefore, the development of an extraction-free sample processing protocol is critical to simplify CRISPR-based diagnostics and increase their user friendliness for POC applications. Several extraction-free sample processing protocols have been developed and applied with CRISPR-based diagnostics, including HUDSON and proteinase K–based treatments (32, 39, 40). These sample processing methods usually require heating steps for efficient lysis of viral particles and inactivation of nucleases. In addition, unlike conventional extraction methods that usually concentrate the extracted RNA, direct application of the treated sample to detection reactions introduces an upper bound and limited amount of inactivated sample input, resulting in decreased detection sensitivity (34). Therefore, we sought to develop a simple extraction protocol that can be performed at ambient temperature, thus avoiding the need for heating steps, allowing the processing of large sample volumes, and increasing input via sample concentration. To this end, we employed a viral RNA extraction buffer (Sigma Aldrich) that provides rapid sample lysis and coupled it with sample concentration using magnetic beads. This RNA extraction buffer has several advantages, including short processing time (5 min), room temperature incubation, stabilization of the released RNA, and compatibility with different sample types such as viral transport medium (VTM) and saliva. To streamline the protocol and allow the processing and RNA concentration from a large sample volume, we combined the sample lysis step with binding and concentration of released RNA using magnetic beads in a single step. This rapid protocol allows both steps (sample lysis and RNA binding to beads) to occur at room temperature in a short period of time (5 min) (*SI Appendix*, Fig. S14*A*). We first investigated the ability of this method to lyse cells and capture the released RNA by detecting the RNase P template using oropharyngeal swabs collected from healthy donors and stored in VTM. RNA was released and concentrated from 200 μL VTM into 30 μL final volume. To maximize the amount of sample input, we doubled the OPTIMA-dx reaction volume (50 instead of 25 μL), which allowed us to use up to 15 μL concentrated RNA sample. We found that the extraction protocol could efficiently lyse cells and capture target templates, which enabled efficient detection of RNase P internal control with OPTIMA-dx (*SI Appendix*, Fig. S14*B*). Next, we tested the performance of the extraction protocol and OPTIMA-dx with oropharyngeal swabs collected from healthy donors and stored in VTM that was spiked with different concentrations of noninfectious SARS-CoV-2 virus particles. OPTIMA-dx was able to detect viral load as low as 5,000 cp/sample (∼50 cp/μL of reaction) and RNase P in all tested reactions (*SI Appendix*, Fig. S14*C*).

Given the encouraging performance of the developed extraction protocol with OPTIMA-dx detection, we next validated this assay on clinical samples. We obtained 22 individual oropharyngeal swabs collected from patients with COVID-19 and two oropharyngeal swabs collected from healthy donors stored in VTM. Some of these samples had previously undergone RNA extraction and been tested with qRT-PCR in a diagnostic laboratory, allowing us to compare our test results with the obtained qRT-PCR Ct values. We processed these samples with the developed extraction procedure and performed OPTIMA-dx. Our assay correctly detected all positive clinical samples with Ct values <34, indicating that the performance of OPTIMA-dx with the developed extraction protocol is equivalent to the performance observed with extracted RNA (*SI Appendix*, Fig. S14*D*).

### One-Pot Multiplexed Nucleic Acid Detection with Thermostable AapCas12b and TccCas13a Enzymes.

**Fig. 6.**
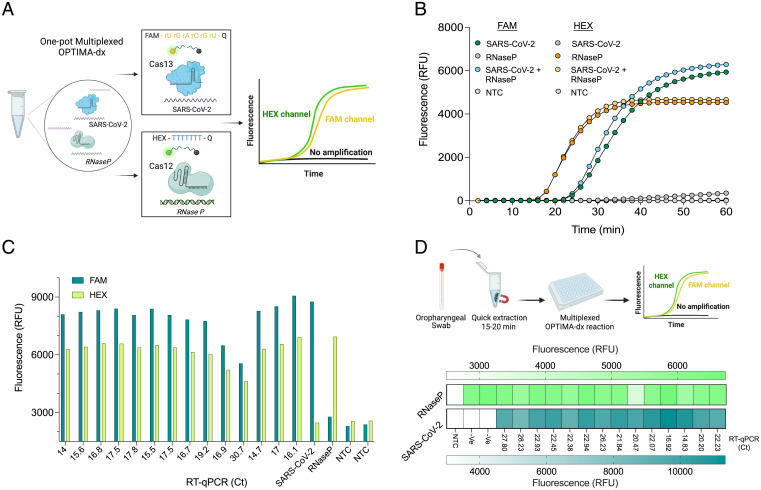
Multiplexed OPTIMA-dx detection with TccCas13a and AapCas12b thermostable Cas enzymes. (*A*) Schematic representation of one-pot multiplexed OPTIMA-dx detection reaction. The unique collateral activity of Cas12 and Cas13 orthologs enables the use of different reporter molecules with different fluorophores (RNA reporter with FAM fluorophore for Cas13 and DNA reporter with HEX fluorophore for Cas12) and multichannel detection. (*B*) Performance of multiplexed detection of SARS-CoV-2 (at 400 cp/μL) and isolated human RNA (for RNase P detection) as measured by real-time fluorescence. Data are shown as means ± SD (*n* = 3). (*C*) Multiplexed detection of SARS-CoV-2 and the human internal control (RNase P) in RNA extracted from 14 clinical COVID-19 samples. Detection reactions were incubated at 56 °C, and the end-point fluorescence signal was measured with FAM and HEX channels after 1 h. SARS-CoV-2, synthetic SARS-CoV-2 RNA used at 400 cp/μL; RNase P, isolated total human RNA; NTC, no template control. (*D*) Multiplexed detection of SARS-CoV-2 and the human internal control (RNase P) from 16 clinical oropharyngeal swabs processed with the quick extraction method. Detection reactions were incubated at 56 °C, and the end-point fluorescence signal was measured with FAM and HEX channels after 1 h. -Ve, SARS-CoV-2–negative samples as determined with qRT-PCR. RFU, relative fluorescence unit.

An ideal diagnostic platform should allow multiplexed detection of more than one target in a single reaction (41). Most of the developed CRISPR-based detection platforms detect only one pathogen or target in a given reaction. This can be attributed mainly to the nonspecific collateral cleavage activity of CRISPR-Cas systems that complicates the integration of more than one reporter in a single reaction. Therefore, in many cases, several separate and independent reactions are developed to detect different targets or internal controls (29, 42, 43). The development of a one-pot multiplexed detection reaction is of great importance to improve the detection efficiency, accuracy, and clinical applicability of CRISPR-based diagnostics. Recently, a Cas12b enzyme from *Alicyclobacillus acidiphilus* (AapCas12b) was shown to be thermostable and can be coupled with RT-LAMP for the detection of SARS-CoV-2 (34). Different from Cas13, Cas12 collateral activity cleaves ssDNA molecules (44). Therefore, we reasoned that since both TccCas13a and AapCas12b are thermostable enzymes and have distinct *cis* and *trans* cleavage activities, we could utilize the specific cleavage preference of these two enzymes to develop a one-pot RT-LAMP–coupled multiplexed detection assay. Therefore, we sought to develop a one-pot multiplexed reaction to detect SARS-CoV-2 and the internal control RNase P in the same reaction. We decided to use the already established and optimized TccCas13a reaction for the detection of SARS-CoV-2 using FAM-labeled RNA reporters and develop AapCas12b-based detection of RNase P with the use of HEX-labeled ssDNA reporters, which would allow differentiation between the two fluorescent signals using different detection channels (Fig. 6*A*). To this end, we designed three different AapCas12b single-guide RNAs (sgRNAs) targeting the LAMP amplicon amplified by the primers used for RNase P detection, but with the FIP primer lacking the T7 promoter. We screened the three different sgRNAs in a one-pot RT-LAMP–coupled AapCas12b RNase P detection reaction using the same OPTIMA-dx reaction components and conditions and found that all tested sgRNAs showed comparable performance and mediated robust detection as measured from the HEX fluorescence signal (*SI Appendix*, Fig. S15*A*). However, sgRNAs 1 and 3 showed faster and more specific signals compared with sgRNA 2. Therefore, we selected sgRNA 1 to develop the one-pot multiplexed OPTIMA-dx reaction. Next, we tested if a multiplexed RT-LAMP reaction is possible and if the Cas12- and Cas13-mediated fluorescent signals can be produced and distinguished from each other. The reaction mix contained both RT-LAMP primer sets for the detection of SARS-CoV-2 and RNase P targets and both FAM and HEX reporters. We found that we could simultaneously detect both SARS-CoV-2 and RNase P in the same reaction using HEX and FAM channels without any fluorescence signal interference from each Cas enzyme’s collateral activity (Fig. 6*B* and *SI Appendix*, Fig. S15*B*).

Next, we evaluated the performance of the multiplexed OPTIMA-dx reaction for the detection of SARS-CoV-2 and the internal control RNase P on RNA extracted from 14 clinical COVID-19 samples. The OPTIMA-dx multiplexed reaction showed an unambiguous positive result for both SARS-CoV-2 and RNase P in all tested clinical samples (Fig. 6*C*). We next sought to evaluate our multiplexed detection assay on clinical swabs using the simple and quick crude sample extraction method developed before (*SI Appendix*, Fig. S14). To this end, we obtained 14 oropharyngeal swabs from patients with COVID-19 infection and two COVID-19–negative swabs. We processed these samples with the extraction procedure and performed OPTIMA-dx multiplexed detection of both SARS-CoV-2 and RNase P. The multiplexed OPTIMA-dx reaction reliably detected both SARS-CoV-2 and RNase P in all SARS-CoV-2–positive samples in 60 min (Fig. 6*D*).

Although the OPTIMA-dx master mix showed strong stability when stored at −20 °C (*SI Appendix*, Fig. S12*B*), we sought to test whether the OPTIMA-dx reaction components can be freeze dried, which would further simplify storage and distribution for POC applications. Therefore, we freeze dried the OPTIMA-dx reaction for multiplexed detection and tested the reaction with the same samples processed with the quick extraction protocol in Fig. 6*D*. We found that the OPTIMA-dx reaction remained functional and was able to detect SARS-CoV-2 and RNase P in all tested samples (*SI Appendix*, Fig. S16). However, a reduction in the reaction speed and performance was observed, and further optimizations are needed to enhance the freeze-drying process.

### Development of a Mobile Phone Application to Collect and Share SARS-CoV-2 Test Results.

Fluorescence detection devices like any plate reader or real-time PCR machine are choice devices to measure any end-point or real-time signal in a sample. However, in POC settings with fewer resources and no specialized training, smartphone-based imaging is becoming popular in biomedical applications for easy data accessibility and sharing. To facilitate data collection and sharing of SARS-CoV-2 test results as well as interpretation of the OPTIMA-dx readout, we developed a deep learning–based approach to design and develop a mobile phone application capable of collecting and reading OPTIMA-dx results from the low-cost P51 Molecular Fluorescence Viewer in POC settings (*SI Appendix*, Fig. S17).

We validated the ability of the OPTIMA-dx smartphone application to identify and call positive and negative readouts from OPTIMA-dx results; the application correctly determined the fluorescence status of each sample with good accuracy (*SI Appendix*, Fig. S18). We next evaluated the application on the 45 clinical samples tested with OPTIMA-dx in *SI Appendix*, Fig. S13; the application identified 38 of the 45 samples as positive. Notably, the two SARS-CoV-2–positive samples deemed negative by the application had the highest Ct values (31, 34) and therefore the lowest intense fluorescence signal of all samples (*SI Appendix*, Fig. S19). We also ran OPTIMA-dx for RNase P in the same samples, resulting in 43 samples testing positive for RNase P of the 45 samples tested, using the OPTIMA-dx one-pot assay and application (*SI Appendix*, Fig. S20). We conclude that OPTIMA-dx can reach a performance of 95% sensitivity and 100% specificity in patient samples when combined with the mobile application, exhibiting high concordance with qRT-PCR data.

### Versatility of OPTIMA-dx for Pathogen Diagnostics.

The one-pot detection assay of OPTIMA-dx can be adapted for the detection of other pathogens. To demonstrate the versatility of OPTIMA-dx, we also employed the system for the detection of the human RNA virus hepatitis C virus (HCV) and the plant ssDNA virus tomato yellow leaf curl virus (TYLCV). Using in vitro–transcribed RNAs of two common HCV genotypes, we showed that OPTIMA-dx can efficiently detect the two viruses within 1 h (*SI Appendix*, Fig. S21*A*). In addition, we used OPTIMA-dx to detect TYLCV ssDNA virus isolated from plants infected with the virus. After DNA isolation from infected, as well as noninfected (healthy), plants, we diluted the extracted DNA by 1:10 and 1:100 and used the diluted DNA as a template for OPTIMA-dx reactions. OPTIMA-dx detected the virus only in the DNA extracted from infected plants in both DNA dilutions within 1 h, indicating the high sensitivity and specificity of the OPTIMA-dx platform for efficient detection of plant DNA viruses (*SI Appendix*, Fig. S21*B*). Having established multiplexed detection of SARS-CoV-2 and RNase P with OPTIMA-dx, we also showed the versatility of OPTIMA-dx for multiplexed detection of HCV and RNase P internal control, further demonstrating the capability of OPTIMA-dx for multiplexed detection (*SI Appendix*, Fig. S21*C*).

## Discussion

In this study, we provide 1) the identification and characterization of thermophilic Cas13a enzymes, thereby expanding the molecular engineering toolbox of CRISPR systems for RNA substrates; 2) a report of a one-pot assay using RT-LAMP coupled to Cas13 for specific and sensitive detection of SARS-CoV-2 to facilitate POC applications and limit cross-contamination; 3) the utilization of the identified thermostable Cas13 with the previously characterized thermostable Cas12b enzyme to develop a one-pot multiplexed detection reaction; 4) the development and use of a mobile phone application coupled with portable, affordable P51 detection to collect and share testing data with central facilities.

Since their discovery, type VI CRISPR Cas13 systems have provided efficient and versatile tools for RNA manipulation (7, 45). However, all Cas13 work to date has been restricted to temperatures around or below 42 °C. Mining data sets from diverse natural contexts, including thermophiles, helped us uncover thermostable variants that evolved naturally to provide immunity to their hosts. These thermophilic Cas13a enzymes will open diverse biotechnological applications for RNA-guided Cas13a RNases at a broad temperature range and under harsh experimental or environmental conditions, especially if complexed with their corresponding crRNAs. For example, interest in Cas13 has recently been growing for therapeutics and disease research, including cancer gene therapy and antiviral therapeutics (46–49). However, in vivo protein stability is critical for successful applications (50), and some proteins, such as LwaCas13a, mediate efficient knockdown only when fused to a stabilizing domain (51). Notably, thermostabilization of proteins results in better stability in vivo (52, 53). Therefore, the robust activity and thermostability of TccCas13a make this protein a promising candidate for further structural studies and potential in vivo RNA targeting applications. Moreover, thermostable enzymes (including thermostable Cas9) have enabled important genome editing applications in thermophiles (54); specific RNA targeting at elevated temperatures beyond the range of previously reported Cas13 proteins is key to creating new tools for use in industrially important thermophiles, for which no CRISPR-Cas13 system has been reported.

Our phylogenetic analysis showed that HheCas13a and TccCas13a are evolutionarily closely related (Fig. 1*A*). Despite the strong conservation of precrRNA processing activity among Cas13 orthologs, HheCas13a has been shown to be the only known precrRNA processing–defective Cas13 (36). Interestingly, in addition to both proteins being thermostable Cas13s, our data show that both HheCas13a and TccCas13a are incapable of processing precrRNA. These observations could indicate a possible relationship between thermostability and the lack of precrRNA processing activity in these Cas13 variants, and future structural studies could explore the mechanism behind the lack of precrRNA processing activity and the thermostability of these proteins.

Besides the wide use of CRISPR-Cas13 enzymes for in vivo applications (55), Cas13 proteins have been increasingly used to develop various diagnostic platforms (41), and their usefulness for the development of diagnostics has become apparent during the COVID-19 pandemic (30–32, 56–58). LAMP isothermal amplification was adapted to develop sensitive CRISPR diagnostics for SARS-CoV-2 detection, including DETECTR (29), iSCAN (59), DISCoVER (Cas13-based module) (30), and an FDA-authorized CRISPR-Cas13–based diagnostics test (SHERLOCK CRISPR SARS-CoV-2 Kit; Integrated DNA Technologies). However, all the above diagnostic methods are performed in two-pot settings, as their Cas enzymes function at ∼37 °C and cannot tolerate the high temperatures needed for LAMP (∼55 to 65 °C). Such two-pot settings are not ideal for POC settings due to the increased chance of cross-contamination, which can be overcome with the implementation of thermostable DNA-targeting Cas12 proteins (34, 60). Therefore, our thermophilic Cas13a protein offers a great advance in diagnostics and will enable the development of other applications.

We showed here that by coupling the activity of both thermostable Cas12 and Cas13 enzymes and the specific recognition of correct amplicons by each CRISPR-Cas system, OPTIMA-dx can be used for multiplexed detection of more than one target in a single reaction. The multiplexed detection capability of OPTIMA-dx could enable additional nucleic acid detection applications, including detecting different virus variants or different pathogens, such as other common respiratory viruses or bacteria in the same reaction. Future developments will include a visual readout of multiplex detection reactions for simple applications at POC.

During this work, we characterized two different thermostable Cas13 enzymes and aimed to find a thermostable Cas13 that could be adapted for the development of a one-pot RT-LAMP–coupled Cas13 detection reaction. We found that although HheCas13a showed good thermostability and activity in a two-pot system, we could not observe good performance in a one-pot reaction. However, this does not exclude the compatibility of HheCas13a for one-pot detection reactions, and further work could optimize HheCas13a activity for use in one-pot detection reactions. The establishment of HheCas13a for one-pot RT-LAMP–based detection reactions would help advance Cas13-based diagnostics and allow the development of different multiplexed one-pot detection assays with TccCas13a and AapCas12b enzymes, which can be achieved by using additional fluorophores and additional detection channels.

Our OPTIMA-dx detection module has several other advantages that make it suitable for POC applications. OPTIMA-dx demonstrated excellent sensitivity, with an LoD of 10 cp/μL synthetic SARS-CoV-2 RNA, and can detect samples with Ct values up to 34. Therefore, OPTIMA-dx exhibited robust sensitivity that would enable its use for reliable SARS-CoV-2 detection in clinical samples. In addition, besides the strong stability of the OPTIMA-dx reaction master mix at −20 °C and tolerance of multiple freeze-thaw cycles, we also demonstrated that OPTIMA-dx reagents can be lyophilized, which would facilitate preassembly of OPTIMA-dx reactions for transportation or long-term storage for large-scale screening in POC settings or remote areas. Moreover, our OPTIMA-dx detection module does not require RNA extraction and is compatible with simple lysis and extraction methods. The ambient temperature sample lysis and concentration method further increases the simplicity of our assay for POC applications. However, considering that this field is rapidly advancing, with the state of the art continuing to evolve, future work would include the development of simple extraction methods that could allow sample processing and OPTIMA-dx–based detection in the same step, avoiding the need for additional liquid-handling steps. Finally, we integrated our detection module with the portable P51 Molecular Fluorescence Viewer to facilitate sample readout and developed a machine-learning module for efficient data collection and sharing of the test results. Overall, the software provides an additional diagnosis validation and enables fast data sharing, making the entire diagnostic process affordable and accessible to a larger section of society.

In conclusion, we characterized a thermophilic Cas13a variant and developed a one-pot RT-LAMP–coupled CRISPR-Cas13a assay for sensitive and specific SARS-CoV-2 detection. Our work provides a viable platform for COVID-19 detection in limited-resource settings. We envision that our current detection module will be used to build a device for at-home or POC testing for COVID-19 and other pathogens. Moreover, the thermophilic Cas13a variants reported in this work will have other applications beyond diagnostics, including in RNA knockdown, editing, imaging, and virus interference. This work thus expands the applications of CRISPR-Cas13 systems and offers possibilities for transcriptome engineering and diagnostics at higher temperatures.

## Materials and Methods

For further details, refer to *SI Appendix*, *Materials and Methods*.

### OPTIMA-dx Reaction.

A detailed protocol for the OPTIMA-dx reaction setup is provided in *SI Appendix*, note 1.

### One-Pot Multiplexed OPTIMA-dx Reaction.

A detailed protocol for the multiplexed OPTIMA-dx reaction setup is provided in *SI Appendix*, note 2.

### Development of OPTIMA-dx Mobile Application.

See *SI Appendix*, note 3 for a detailed description.

## Supplementary Material

Supplementary File

## Data Availability

All study data are included in the article and/or *SI Appendix*.
