# Supplementary material for: Characterization of a thermostable Cas13 enzyme for one-pot detection of SARS-CoV-2
Source: Proc Natl Acad Sci U S A. 2022 Jun 28;119(28):e2118260119. doi: 10.1073/pnas.2118260119 (PMC9282225; doi:10.1073/pnas.2118260119)
Supplement: Supplementary File [file pnas.2118260119.sapp.pdf]

PNAS
www.pnas.org

**Supplementary Information for**

# Characterization of a thermostable Cas13 enzyme for one-pot detection of SARS-CoV-2

Ahmed Mahas[1], Tin Marsic[1], Mauricio Lopez-Portillo Masson[1], Qiaochu Wang[1], Rashid Aman[1], Cheng Zheng[2], Zahir Ali[1], Madain Alsanea[3], Ahmed Al-Qahtani[3], Bernard Ghanem[2], Fatimah Alhamlan[3], and Magdy Mahfouz[1, *]

[1]*Laboratory for Genome Engineering and Synthetic Biology, Division of Biological Sciences, 4700 King Abdullah University of Science and Technology, Thuwal 23955-6900, Saudi Arabia.*
[2] *Image and Video Understanding laboratory, Computer, electrical, mathematical sciences and engineering, 4700 King Abdullah University of Science and Technology, Thuwal 23955-6900, Saudi Arabia.*
[3]Department of Infection and Immunity, King Faisal Specialist Hospital and Research Centre. Riyadh 11564, Saudi Arabia

***Correspondence**: Magdy M. Mahfouz (magdy.mahfouz@kaust.edu.sa)

**This PDF file includes:**

Supplementary material and methods
Figures S1 to S21
Tables S1 to S9
Supplementary notes 1 to 3
SI References

## Supplementary material and methods

### Computational Identification of a Thermophilic CRISPR-Cas13a.

We manually interrogated various existing Cas13 enzymes and their bacterial hosts to identify potential thermophilic Cas13s originating from thermophilic organisms. After the identification of HheCas13a as a potential thermophilic Cas13 protein, we used its protein sequence as a query for BLAST analysis against the National Center for Biotechnology Information nonredundant protein database using default settings. Only sequences with query coverage above 80% were considered for a second round of host interrogation (focused on growth conditions using the BacDive database [https://bacdive.dsmz.de/] and other resources). We identified TccCas13a (accession #WP_149678719.1) from *T. caenicola* (strain DSM 19027) as another potential thermophilic Cas13 protein. A phylogenetic tree was constructed using protein sequences of different Cas13 proteins belonging to different families/subtypes of class II/type VI CRISPR-Cas systems. All protein sequences were organized in a single .txt file and aligned using MUSCLE in MEGA X software with default settings. The phylogenetic reconstruction was based on the maximum-likelihood method with the WAG+G+F model and 1,000 bootstrap samplings. The generated output file (.nwk) was visualized using TreeGraph_2. The *T. caenicola* genome (GenBank #NZ_FQZP01000023.1) was submitted to the CRISPRCasFinder program [1] to identify the associated CRISPR array. CRISPRDetect [2] was then used to predict the orientation of the direct repeat in the TccCas13a CRISPR array.

### Cas proteins production and purification

The expression vector pC013-Twinstrep-SUMO-huLwCas13a_WT for the production of LwaCas13a was obtained from Addgene (plasmid #90097); the purification of recombinant

LwaCas13a was performed following a previously published protocol [3] .The expression vector p2CT-His-MBP-Hhe_Cas13a_WT for the production of HheCas13a was obtained from Addgene (plasmid #91871) and the purification of the recombinant HheCas13a was performed following a previously published protocol [4]. To generate the plasmid for TccCas13a production and purification, the *TccCas13a* coding sequence codon-optimized for *E. coli* was synthesized (GenScript) *de novo* and subcloned in-frame downstream of the sequences encoding the His and SUMO tags into the His6-TwinStrep-SUMO bacterial expression vector (Addgene #90097) by replacing the LwaCas13a encoding sequence with TccCas13a sequence using the BamHI and NotI restriction sites. Purification of the TccCas13a protein was performed following the protocol of Kellner *et al.* (2019) [3] with a few modifications. Briefly, the TccCas13a expression vector was transformed into the *E. coli* strain BL21. Starter cultures were prepared by growing single colonies in LB broth containing 100 µg/mL ampicillin for about 12 h at 37°C. Next, 25 mL of starter culture was used to inoculate 1 L of Terrific Broth (IBI Scientific) containing 100 µg/mL ampicillin, and the 1-L cultures (4 L total) were incubated at 37°C until an $OD_{600}$ of ~0.5. Cells were then placed at 4°C for 30 min, and protein production was induced with the addition of 0.5 mM IPTG (isopropyl ß-D-1-thiogalactopyranoside). Cultures were then incubated overnight at 16°C with shaking at 180 rpm. Next, cells were harvested by centrifugation for 20 min at 4°C at 4,000 rpm. Cell pellets were resuspended in lysis buffer (50 mM Tris-HCl pH 7.5, 300 mM NaCl, 5% glycerol, 1 mM TCEP [tris(2-carboxyethyl)phosphine], 4.5 mM $MgCl_2$, 1 mM PMSF, EDTA-free protease inhibitor [Roche]) and with 1 mg/mL lysozyme (L6876, Sigma). Cells were lysed by sonication and clarified by centrifugation at 12,000 rpm for 60 min at 4 °C. The soluble 6xHis-SUMO-TccCas13a protein was then purified from cleared lysate with an affinity chromatography column (HisTrap HP, 5 mL GE Healthcare) (AKTA PURE, GE Healthcare) followed by

concurrent removal of the 6xHis-SUMO tag by digestion with the SUMO protease and overnight dialysis in dialysis buffer (50 mM Tris-HCl pH 7.5, 200 mM KCl, 5% glycerol, 1 mM TCEP). The cleaved protein was concentrated to 1.5 mL by Amicon Ultra-15 Centrifugal Filter Units (100 kDa NMWL, UFC905024, Millipore) and further purified via size-exclusion chromatography on a S200 column (GE Healthcare) in gel filtration buffer (50 mM Tris-HCl, 200 mM KCl, 10% glycerol, 1 mM TCEP, pH 7.5). The protein-containing fractions resulting from the gel filtration were pooled, snap-frozen, and stored at –80°C.

The expression vector pAG001- His6-TwinStrep-SUMO-AapCas12b for the production of AapCas12b was obtained from Addgene (plasmid #153162); and the purification of recombinant AapCas12b was performed by Genscript.

**Differential scanning fluorimetry (DSF)**

DSF was performed using 5 to 15 μM of purified Cas13 protein in gel filtration buffer (with 5% glycerol) containing 10X SYPRO Orange fluorescent dye (S6650, ThermoFisher) in a final reaction volume of 35 μL. Proteins were tested in triplicates and the fluorescence was monitored using a 96-well Real-Time PCR detection system (CFX96 qPCR machine, Bio-Rad), from 25°C to 95°C, with a gradual temperature increase of 1°C every 10 s.

**Protein thermostability assay**

LwaCas13a, HheCas13a and TccCas13a proteins were diluted to approximately 0.2 mg/mL in protein storage buffer (50 mM Tris-HCl pH 7.5, 600 mM NaCl, 5% glycerol, 2 mM DTT) and incubated at a range of temperatures (37, 60, 70, and 90°C) for 30 min. Samples were centrifuged in a microcentrifuge at 14,200 rpm for 25 min. A total of 5 μL of the supernatant was mixed with

the same volume of protein sample loading buffer and heated at 95°C for 10 min. The samples were allowed to cool down on ice for 3 min and run on a 10% NuPAGE Bis-Tris polyacrylamide gel (NP0301BOX, ThermoFisher). Protein thermostability assays for HheCas13a and TccCas13a ribonucleoproteins (RNPs) was performed similarly after an initial incubation of the proteins with 1 μM of their cognate crRNAs for 5 min at 37°C in order to assemble the RNP before subjecting them to a range of temperatures. BenchMark™ Pre-stained Protein Ladder was used as a marker (10748010, Invitrogen).

**Nucleic acid preparation**

A short region of the SARS-CoV-2 *N* gene sequence was used as the target sequence in all preliminary characterization and optimization experiments of thermophilic Cas13 to screen reporters and assess Cas13 protein thermostability. The *N* gene target RNA sequence was prepared by *in vitro* transcription of PCR amplicons containing the T7 promoter sequence using the 2019-nCoV_N_Positive Control plasmid (10006625, IDT) as PCR template. For LwaCas13a targeting, short region of SARS-CoV-2 *ORF1* was synthesized as gBlock for subsequent PCR amplification and *in vitro* transcription. In addition, HCV genotypes were also synthesized as gBlock and were PCR amplified for subsequent *in vitro* transcription (PCR primers and gBlocks are listed in Table S4 and S5). PCR amplicons were purified (QIAquick PCR Purification Kit, QIAGEN) and transcribed *in vitro* using the HiScribe T7 Quick High Yield RNA Synthesis Kit (E2050, NEB). The transcripts were then purified with Direct-zol RNA Miniprep Kits (R2050, Zymo Research) following the manufacturer's instructions, and the purified RNA was stored at –80°C.

For production of LwaCas13a, HheCas13a, and TccCas13a crRNAs, templates for *in vitro* transcription were generated using single-stranded DNA oligos (IDT) containing a T7 promoter, scaffold, and spacer in reverse complement orientation, and were then annealed to the T7 forward

primer in Taq DNA polymerase buffer (Invitrogen). The annealed oligos were then used as templates for subsequent *in vitro* transcription as described above.

For production of AapCas12b sgRNAs, the AapCas12b 91 nt-long sgRNA scaffold was synthesized and ordered as sense ssDNA ultramer containing the T7 promoter at the 5` end (IDT). The scaffold was used as a template for PCR with T7 forward primer and reverse primers containing 20 nt-long spacer sequences. (All crRNAs oligos are listed in Table S2).

To establish the thermophilic Cas13-based one-pot assay, control synthetic SARS-CoV-2 viral genomic sequences were ordered as synthetic RNA (Twist Bioscience, 102024), diluted to 10,000 RNA copies/µL and used at the indicated concentrations.

For RT-LAMP amplification (described below), previously published LAMP primers designed to amplify the SARS-CoV-2 *N* gene [5] were used, with the following modifications. The FIP or BIP primers were designed with the T7 promoter sequence appended at the 5' end of the first half of the primers. However, for RNase P detection in multiplexed reactions, regular LAMP primers lacking T7 promoter sequence were used.

### *In vitro cis* cleavage assays

HheCas13a and TccCas13a cleavage reactions were performed at 37°C and 60°C with synthetic, *in vitro*-transcribed RNA targets. Briefly, for both HheCas13a or TccCas13a cleavage assays, cleavage reactions were carried out in 20-µL reaction volume with 50 nM of either Cas13a protein, 50 nM of their cognate crRNAs, and 100 nM of target RNA in 1x isothermal buffer (20 mM Tris-

HCl pH 8.8, 50 mM KCl, 10 mM $(NH_4)_2SO_4$, 2 mM $MgSO_4$, 0.1% Tween 20 (B0537, NEB)) supplemented with an additional 6 mM $MgSO_4$ (final of 8 mM $MgSO_4$); the reactions were then incubated at the indicated temperatures for 1 h (no pre-assembly of Cas13a protein and crRNA to form RNP was performed). The samples were then boiled at 70°C for 3 min in 2X RNA Loading Dye (B0363S, NEB) and cooled down on ice for 3 min before loading onto a 6% polyacrylamide-urea denaturing gel. Electrophoresis was conducted for 45 min at 25 W. The gels were stained with SYBR Gold Nucleic Acid Gel Stain (S11494, ThermoFisher) for 10 min, briefly washed with 1X Tris-borate EDTA buffer and visualized using a Bio-Rad Molecular Imager Gel Doc system.

**Fluorescent ssRNA cleavage assays**

For reporter screening and other fluorescence-based assays, 50 nM of Cas13a recombinant proteins was incubated with 50 nM of their respective crRNAs, 250 nM of ssRNA reporter in 1X isothermal buffer (B0537, NEB) supplemented with an additional 6 mM $MgSO_4$ (final 8 mM $MgSO_4$), 0.8 U/µL RNaseOUT (10777019, Invitrogen) or RNase inhibitor, Murine (M0314, NEB) and 2 µL of (1-100 nM) target RNA in a 20-µL reaction volume. No pre-assembly of Cas13-crRNA RNP was performed except in Fig 2C, where 200 nM of Cas13 protein was incubated with 200 nM of their respective crRNAs in 1x isothermal buffer for 25 mins, and 5 µL of the 200 nM RNP complex was added to the rest (15 µL) of the reaction components. These reactions were incubated in a 96-well plate at different temperatures for 1 h in a 96-well Real-Time PCR detection system (CFX96 qPCR machine, Bio-Rad), with fluorescence measurements taken every 2 min using the FAM channel.

**Pre-crRNA processing assays**

RNA oligos of 5′ FAM-labelled pre-crRNAs were custom-synthesized (IDT). Pre-crRNA processing assays were performed in 1x isothermal buffer (B0537, NEB) supplemented with an additional 6 mM $MgSO_4$ (final of 8 mM $MgSO_4$) in a 20-μL reaction volume. In all assays, 100 nM of each Cas13a orthologue was incubated with 200 nM of their cognate 5′-FAM labeled pre-crRNAs for 1 hour at different temperatures. The reactions were then heated at 70°C for 3 min in 1X RNA Loading Dye (B0363S, NEB) and cooled down on ice for 3 min before loading onto a 15% polyacrylamide-urea denaturing gel. Electrophoresis was conducted for 80 min at 25 W. The gels were visualized using fluorescein channel in Bio-Rad Molecular Imager Gel Doc system.

**Two-pot detection reactions**

Reverse transcription and LAMP isothermal amplification of target nucleic acids were conducted using the previously reported RT-LAMP primers [5]. Reactions were performed using 1.6 μM FIP/BIP primers (with the T7 promoter sequence added to either the FIP or BIP primer), 0.2 μM F3/B3 primers, and 0.4 μM LF/LB primers in 1X Isothermal Amplification Buffer (20 mM Tris-HCl pH 8.8, 50 mM KCl, 10 mM $(NH_4)_2SO_4$, 2 mM $MgSO_4$, 0.1% Tween 20) (B0537, NEB), 1.4 mM dNTPs, 8 U of Bst 2.0 WarmStart DNA Polymerase (M0538, NEB), 7.5 U of WarmStart RTx Reverse Transcriptase (M0380, NEB) and 6 mM $MgSO_4$ (B1003, NEB) in 25-μL reactions containing 100 cp/μL of SARS-CoV-2 control standards. The reactions were incubated at 62°C for 40 min in a PCR machine (C1000 touch thermal cycler, BioRad).

For subsequent Cas13a-based detection, 50 nM of recombinant HheCas13a or TccCas13a protein was incubated with 50 nM of the respective crRNA, 250 nM of ssRNA reporter (Poly(U) ssRNA reporter for HheCas13a or mix ssRNA reporter for TccCas13a), 0.8 U/μL RNaseOUT, 2 U/μL Hi-T7 RNA polymerase (M0658S, NEB), 1 mM NTPs, and 2 μL of the RT-LAMP reaction product.

Reactions were run in a 96-well plate (BioRad) at 55°C for 1 h in a 96-well Real-Time PCR detection system (CFX96 qPCR machine, Bio-Rad), with fluorescence measurements taken every 2 min using the FAM channel.

**Michaelis-Menten enzyme kinetic parameters calculation**

The trans cleavage activity of TccCas13a was investigated by measuring Michaelis-Menten enzyme kinetic parameters following the protocol introduced by Ramachandran *et al.*[6]. The Michaelis-Menten equation represents the relationship between reaction velocity and substrate concentration, which can be obtained from experimental data:

$$v = \frac{d[P]}{dt} = k_{cat} E_0 \frac{[S]}{K_M + [S]}$$

Where v is reaction velocity, [P] is the concentration of reaction product, $E_0$ is the initial enzyme concentration, and [S] is the substrate concentration. The reporter cleaved by TccCas13a is the reaction product in this assay. To estimate the kinetic parameters of TccCas13a, 0.5 nM of activated RNP was treated with different concentrations of FAM Mix reporters. In detail, 100 nM RNP was first prepared by incubating 100 nM TccCas13a protein, 125 nM crRNA (# 1172), and 1 U of RNase inhibitor (NEB, M0314L) in 1X isothermal amplification buffer (B0537, NEB) supplemented with 6 mM $MgSO_4$ at 56°C for 10 minutes. Next, the *trans* cleavage activity of RNP was activated by mixing 20 nM of *N* gene target with 2 nM RNP in 1 x isothermal amplification buffer supplemented with 6 mM $MgSO_4$ and 1 U of RNase inhibitor and incubated at 56°C for 15 min. For the *trans* cleavage assay, FAM Mix reporter at concentrations of 31.25 nM, 62.5 nM, 125 nM, 250 nM, 500 nM, 1 μM, 2 μM and 4 μM was added into 0.5 nM of target-activated RNP together with 6 mM $MgSO_4$ and 1 U of RNase inhibitor in 1x isothermal amplification buffer in 20 μL of the final volume. The fluorescence readout was measured every 30 s at 56°C (CFX96

qPCR machine, Bio-Rad). The same reactions described above were also carried out in parallel without the addition of crRNA, which were used as controls to subtract the fluorescence background signal. The data were analyzed by GraphPad Prism software (GraphPad, CA, USA) to calculate $K_M$ and $k_{cat}$. First, the data obtained from reactions without crRNA were subtracted from those reactions with crRNA to obtain the true fluorescence generated by enzyme-cleaved reporters. The real-time data from the first 600 s were fitted using linear regression to obtain the initial reaction velocity for different reporter concentrations represented by the increase of fluorescence over time, which can be represented as dF/dt. To convert the fluorescence readout into the concentration of the cleaved product, FAM Mix at 31.25 nM, 62.5 nM, 125 nM, 250 nM, 500 nM, 1 µM, and 2 µM was incubated with 40 µg of RNase A (Invitrogen, cat: 12091-039) in 20 µL reaction at 37°C for more than 3 hours to ensure complete cleavage. By plotting the end-point data and subtracting it from the background using water-only samples over reporter concentration, the relationship between fluorescence readout and reporter concentration can be obtained: $F_P$= a[P]. Where $F_P$ is the fluorescence produced by the cleaved reporter, and a is a constant.

The reaction can then be calculated as follows:

$$v = \frac{dP}{dt} = \frac{1}{a} \times \frac{dF}{dt}$$

The curve for reaction velocity dP/dt over reporter concentration was fitted to the Michaelis−Menten equation to calculate the value of $K_M$ and $V_{max}$. The $k_{cat}$ can be calculated as $V_{max}$ equals the value of $k_{cat}E_0$. To test the validation of calculated kinetic parameters, the back-of-the-envelope test introduced in Ramachandran *et al.* [6] was conducted. For all the tests, an initial linear time portion $t_{lin}$ of 600 s was used to calculate the α, β, and γ values (Table S8).

**Optimization of one-pot detection reactions**

For *Bst* DNA polymerase screening and other optimization reactions, reverse transcription and LAMP isothermal amplification of the target nucleic acids, coupled with T7-mediated *in vitro* transcription and Cas13-based detection of the amplified and *in vitro*-transcribed target RNA, were carried out in the same tube. Reactions were performed using RT-LAMP primers at a final concentration of 1.6 μM for FIP/BIP primers (with the T7 promoter sequence added to either the FIP or BIP primer), 0.2 μM F3/B3 primers, and 0.4 μM LF/LB primers, in 1X Isothermal Amplification Buffer (from a different vendor from the *Bst* DNA polymerase screening reactions) or from Lucigen (30027, Lucigen) in other optimization experiments, 1.4 mM dNTPs, 0.32 U/μL Bst DNA Polymerase (from a different vendor from the Bst DNA polymerase screening reactions) or 2.4 U/μL from Lucigen (30027, Lucigen), 0.3 U/μL of WarmStart RTx Reverse Transcriptase (M0380, NEB), 6 mM $MgSO_4$, 0.8 U/μL RNasin plus (N2611, Promega), 0.5 mM NTPS, 2 U/μL Hi-T7 RNA polymerase (M0658S, NEB), 0.4 U/μL thermostable inorganic pyrophosphatase (M0296, NEB), 250 nM RNA reporter, 50 nM Cas13, 50 nM crRNA, and 2 μL of template RNA in 25-μL reactions. These reactions were incubated in a 96-well plate (BioRad) at 56°C (or as otherwise indicated) for 1–2 h in a 96-well Real-Time PCR detection system (CFX96 qPCR machine, Bio-Rad), with fluorescence measurements taken every 2 min using the FAM channel.

For the detection of HCV, the one-pot detection reactions were performed as described above with the use of 500 pM *in vitro* transcribed RNA template. For the detection of TYLCV, the reactions were performed as described above, but without the addition of RTx reverse transcriptase and with use of 2 μL of 1:10 or 1:100 diluted extracted DNA as template. 1 ng of TYLCV plasmid was used as positive control. All RT-LAMP primers are listed in Table S1.

**OPTIMA-dx Reaction.**

The reaction was performed using RT-LAMP primers at a final concentration of 1.6 µM FIP/BIP primers (with the T7 promoter sequence added to the FIP primer), 0.2 µM F3/B3 primers, and 0.4 µM Loop forward (LF) and loop backward (LB) primers, in 1X isothermal amplification buffer from Lucigen (30027), 1.4 mM dNTPs, 2.4 U/µL Bst DNA polymerase (30027; Lucigen), 0.3 U/µL WarmStart RTx Reverse Transcriptase (M0380; New England BioLabs), 6 mM MgSO , 0.8 U/µL RNasin plus (N2611; Promega), 0.5 mM NTPs, 4 U/µL 4 Hi-T7 RNA polymerase (M0658S; New England BioLabs), 0.4 U/µL thermostable inorganic pyrophosphatase (M0296; New England BioLabs), 1 µM ssRNA FAM reporter or 750 nM ssRNA HEX reporter, 50 nM Cas13, 50 nM crRNA, and 4.5 µL template RNA in 25-µL reactions.

**Screening of AapCas12b sgRNAs in one-pot reaction**

Reactions were performed using RT-LAMP primers at a final concentration of 1.6 µM for FIP/BIP primers, 0.2 µM F3/B3 primers, and 0.2 µM LF/LB primers, in 1X Isothermal Amplification Buffer (30027, Lucigen), 1.4 mM dNTPs, 2.4 U/µL Bst DNA Polymerase (30027, Lucigen), 0.3 U/µL of WarmStart RTx Reverse Transcriptase (M0380, NEB), 6 mM $MgSO_4$, 0.8 U/µL RNasin plus (N2611, Promega), 0.5 mM NTPS, 4 U/µL Hi-T7 RNA polymerase (M0658S, NEB), 0.4 U/µL thermostable inorganic pyrophosphatase (M0296, NEB), 250 nM ssDNA HEX reporter, 50 nM AapCas12b, 50 nM sgRNAs, and 1 µL of total human RNA template in 25-µL reactions. These reactions were incubated in a 96-well plate (BioRad) at 56°C for 1 h in a 96-well Real-Time PCR detection system (CFX96 qPCR machine, Bio-Rad), with fluorescence measurements taken every 2 min using the HEX channel. T7 RNA polymerase and NTPs were included here to test the

compatibility of Cas12b activity with these reagents for the subsequent multiplex detection reactions.

**One-Pot Multiplexed OPTIMA-dx Reaction.**

The multiplexed reaction was performed as described above (OPTIMA-dx reaction) with the following modifica- tions: 50 nM AapCas12b protein, 50 nM AapCas12b sgRNAs-1 for RNase P detection, 250 nM HEX ssDNA reporter (the FAM reporter used at 250 nM instead of 1 μM in multiplexed detection), and RT-LAMP primers for RNase P detection (LF and LB were used at 0.2 μM final concentration) were added to OPTIMA-dx SARS-CoV-2 or HCV detection components. The final reaction volume was 50 μL. These reactions were incubated in a 96-well plate (Bio-Rad Laborato- ries) at 56 °C for 1 to 2 h in a 96-well real-time PCR detection system (CFX96 qPCR machine; Bio-Rad Laboratories), with fluorescence measurements taken every 2 min using both FAM and HEX channels.

**Agroinfiltration inoculation of plants with TYLCV and DNA extraction**

Plant infection with TYLCV infectious clones and subsequent DNA extraction was done following the previous protocol (Mahas *et al. [7]*).

**Clinical sample collection and RNA extraction**.

We obtained the necessary ethical approval (institutional review board: King Faisal Specialist Hospital and Research Centre Research Advisory Council #2200021) for collection of the samples.

Oropharyngeal and nasopharyngeal swabs were collected from suspected COVID-19 patients by physicians in Ministry of Health hospitals in Saudi Arabia and placed in 2-mL screw-capped cryotubes containing 1 mL of TRIZOL for inactivation and transport. Each sample tube was sprayed with 70% ethanol, enveloped with absorbent tissues, and then placed and sealed in individually labeled biohazard bags. The bags were then placed in leak-proof boxes and sprayed with 70% ethanol before placement in a dry ice container for transfer to the lab. Total RNA was extracted from the samples following instructions as described in the CDC EUA-approved protocol and using the Direct-zol kit (Direct-zol RNA Miniprep, Zymo Research; catalog #R2070) following the manufacturer's instructions.

**Extraction-free sample processing and concentration**

A beads-based extraction mixture was prepared as follows. First, beads were prepared by washing 1 mL of Sera-Mag SpeedBeads Carboxyl Magnetic Beads Hydrophobic (GE Healthcare 65152105050250) with 1 mL of UltraPure DNase/RNase-free distilled water (1097705, Invitrogen) twice and then resuspended in 50 mL of beads binding buffer (10 mM Tris-HCl pH 8.0, 1M KCl, 18 % PEG-8000, and 1mM EDTA). Next, 50 mL of extraction mixture was prepared by mixing 32.5 mL of beads (resuspended in binding buffer), 12.5 ml of 4x Viral RNA Extraction Buffer (VRE100, Sigma-Aldrich), and 5 mL of UltraPure DNase/RNase-free distilled water. The extraction mixture was aliquoted in 1.5 mL tubes, 400 µL each.

To process clinical samples, 200 µL of VTM of oropharyngeal swabs were transferred into 400 µL of extraction mixture, vortexed vigorously and incubated at room temperature for 5 min. The mixture was then placed on a magnetic rack (Invitrogen DYNAL bead Separator) for 2-3 mins until the solution gets clear. Next, the supernatant is removed, and beads is resuspended and

washed in 750 uL of 70% ethanol (v/v). Samples are again placed on the magnetic rack for ~ 2 mins until solution gets clear. The supernatant is removed completely and the tubes are left open for 5-10 min to dry. Beads are then resuspended in 30 µL of H2O and vortexed vigorously for 5 seconds and incubated at room temperate for 3 mins. The samples are then placed on the magnetic rack to collect beads, and 15 µL of H2O is transferred into 35 µL of OPTIMA-dx master mix.

For experiment with VTM spiked with non-infectious virus particles, 200 µL of VTM of oropharyngeal swabs collected from healthy donors were spiked with the indicated concentration of non-infectious virus particles (NATSARS(CoV2)-ERC, ZeptoMetrix) or with 200 µL of (NATSARS(CoV2)-NEG, ZeptoMetrix) for negative controls, and the spiked VTM were processed as described above.

**Real-time reverse transcription PCR (RT-PCR) for detecting positive SARS-CoV-2 RNA samples.**

RT-PCR was conducted on extracted RNA samples using the oligonucleotide primer/probe (Integrated DNA Technologies, 641 catalog #10006606) and Superscript III one-step RT-PCR system with Platinum Taq Polymerase (catalog #12574-026) following the manufacturer's protocol.

**Freeze-drying of detection reactions**

Multiplexed OPTIMA-dx detection reactions were assembled as described above in a final volume of 50 µL in 1.5 mL tubes.  Reactions were snap-frozen in liquid nitrogen and transferred to a LABCONCO Acid-Resistant CentriVap Concentrator (supplemented with LABCONCO CentriVap -105°C Cold Trap and Vacuubrand CVC 3000 Vacuum pump) Freeze Dry System for 2–3 hours of freeze-drying at a minimal temperature under the pressure of 1 to 10 millibar until

the water was completely removed. Rehydration of freeze-dried reactions was accomplished with the RNA isolated from clinical samples (20 μL), 25 $H_2O$, and 5 μL of 10X Isothermal Amplification Buffer from Lucigen (30027, Lucigen).

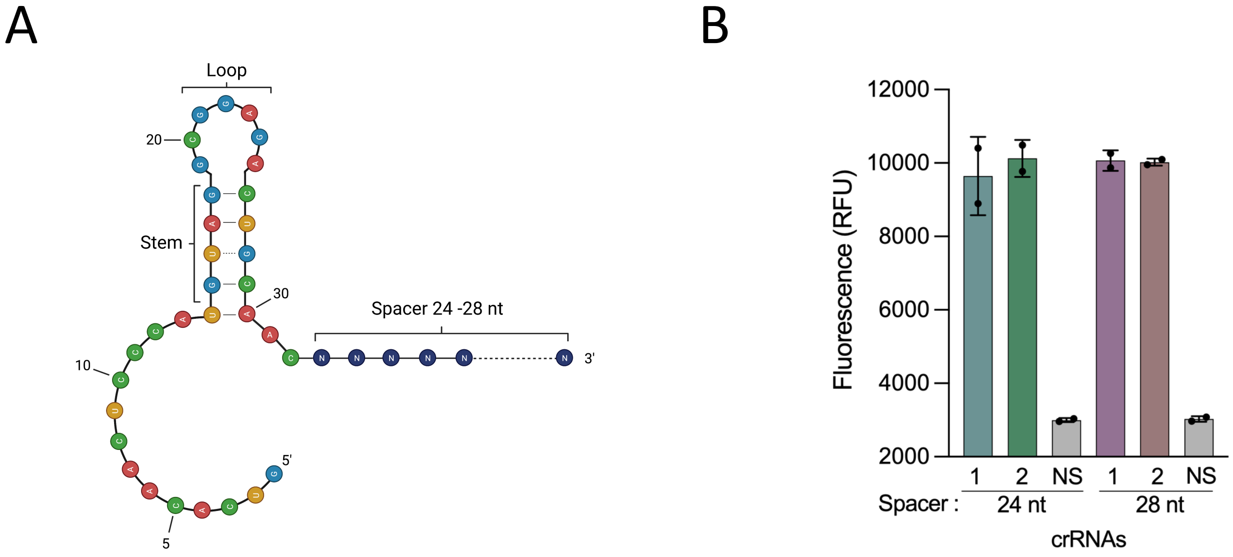


**Supplementary Figure 1: TccCas13a crRNA.**

**A-** Predicted secondary RNA structure of the direct repeat sequence of TccCas13a crRNA. RNAfold (http://rna.tbi.univie.ac.at/) was used to predict the crRNA secondary structure.

**B-** End-point detection of *trans* cleavage activity of TccCas13a after 1 hour incubation using crRNAs with 24 or 28 nt-long spacer sequences. Data shown as mean ± SD ($n = 2$).

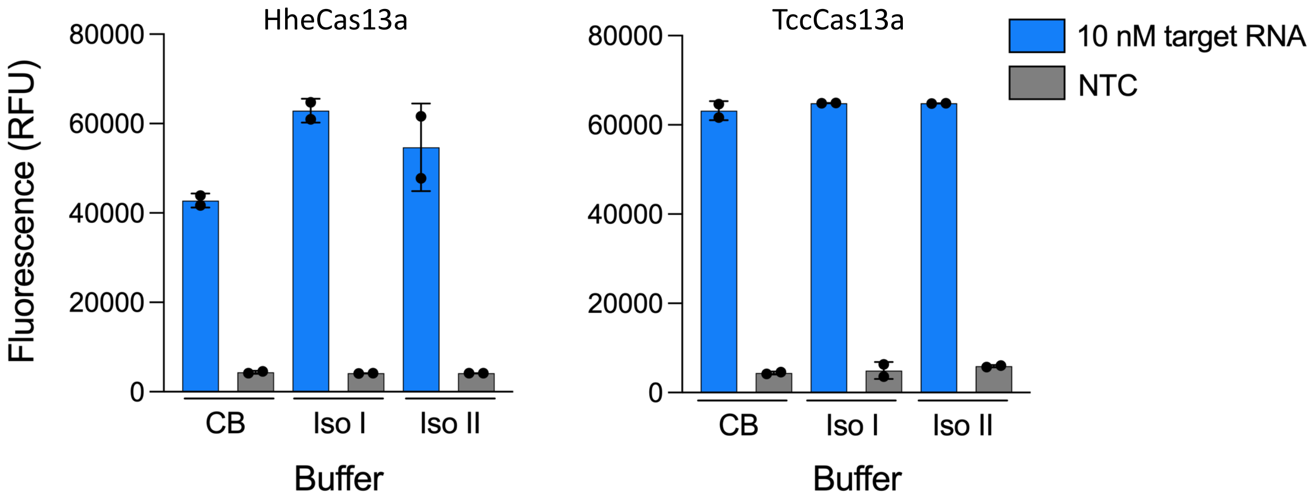


**Supplementary Figure 2: HheCas13a and TccCas13a *trans* cleavage activity in different buffers.**

Effect of different buffers on the *trans* cleavage activity of HheCas13a and TccCas13a. Endpoint fluorescence signal detection was measured after 1 hour. CB: cleavage buffer (20 mM HEPES-Na pH 6.8, 50 mM KCl, 5 mM MgCl2, and 5% glycerol). Iso I: isothermal buffer I (NEB, B0537S). Iso II: isothermal buffer II (NEB, B0374S). Additional 6 mM $MgSO_4$ was added to reactions with Iso I or Iso II buffer. Reactions were incubated at 55 °C.

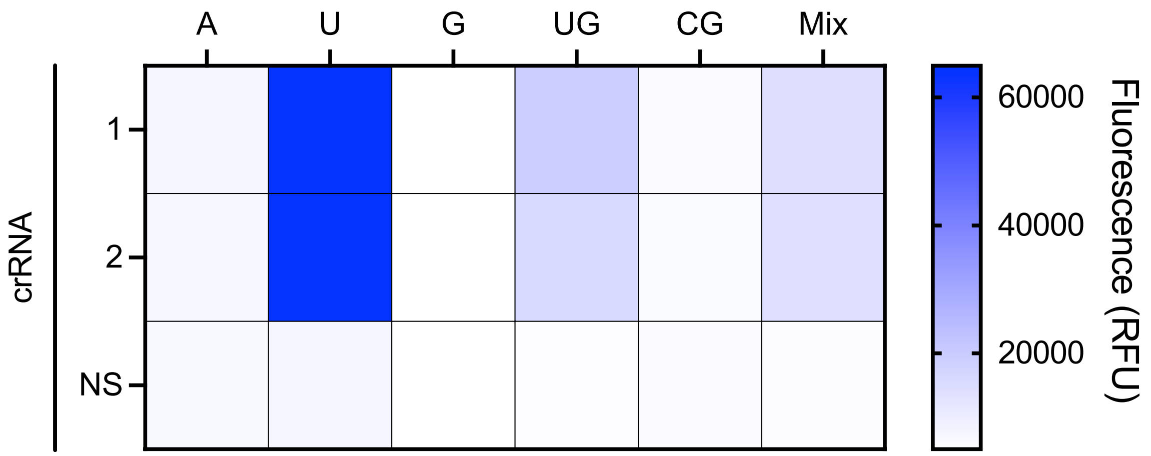


**Supplementary Figure 3: HheCas13a collateral cleavage preference for the ssRNA reporter.**

Reactions consisting of HheCas13a and its respective cognate crRNAs or non-specific crRNA (NS) control were performed in the presence of ssRNA target and one of six ssRNA reporters. NS: non-specific crRNA. Data are shown as mean ($n = 3$). Reactions were incubated at 56 °C and endpoint fluorescence signal detection was measured after 30 min. ssRNA reporter sequences are shown on top of the panel, A: Poly A reporter, U: Poly U reporter, G: Poly 6G reporter, UG: 3(UG) reporter, CG: 3(CG) reporter, Mix: Mix reporter. See Supplementary table 6.

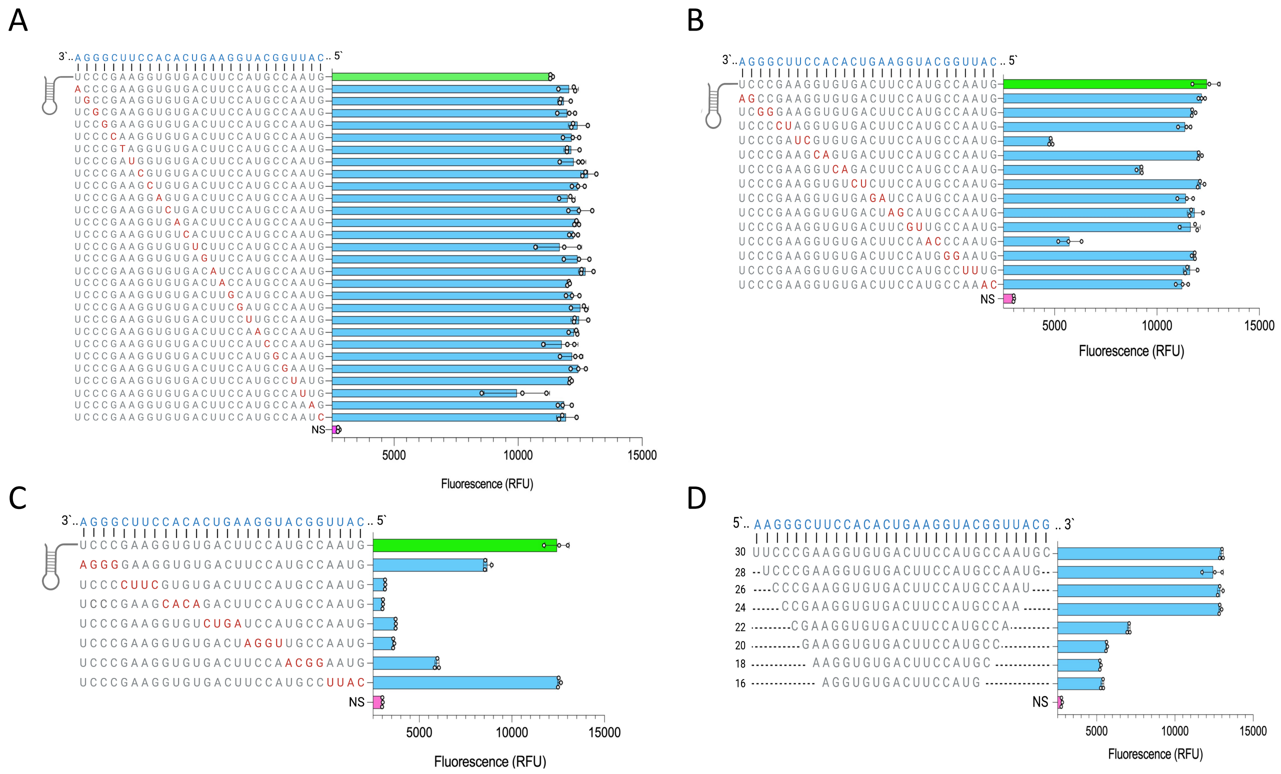


**Supplementary Figure 4: Evaluating the effect of different mismatches between crRNA and target RNA and spacer truncations on HheCas13a activity.**

In each panel, Left: crRNA nucleotide sequence with the positions of mismatches (red) on the crRNA spacer. Right: the fluorescence intensity, relative to the non-specific crRNA control (NS, pink) or crRNA with no mismatches (green), resulting from HheCas13a collateral cleavage activity on each tested crRNA. Reactions were incubated at 56 °C and endpoint fluorescence signal detection was measured after 30 min. Values are shown as mean ± S.D. A) single mismatches. B) double mismatches. C) 4 mismatches. D) crRNA spacer truncations.

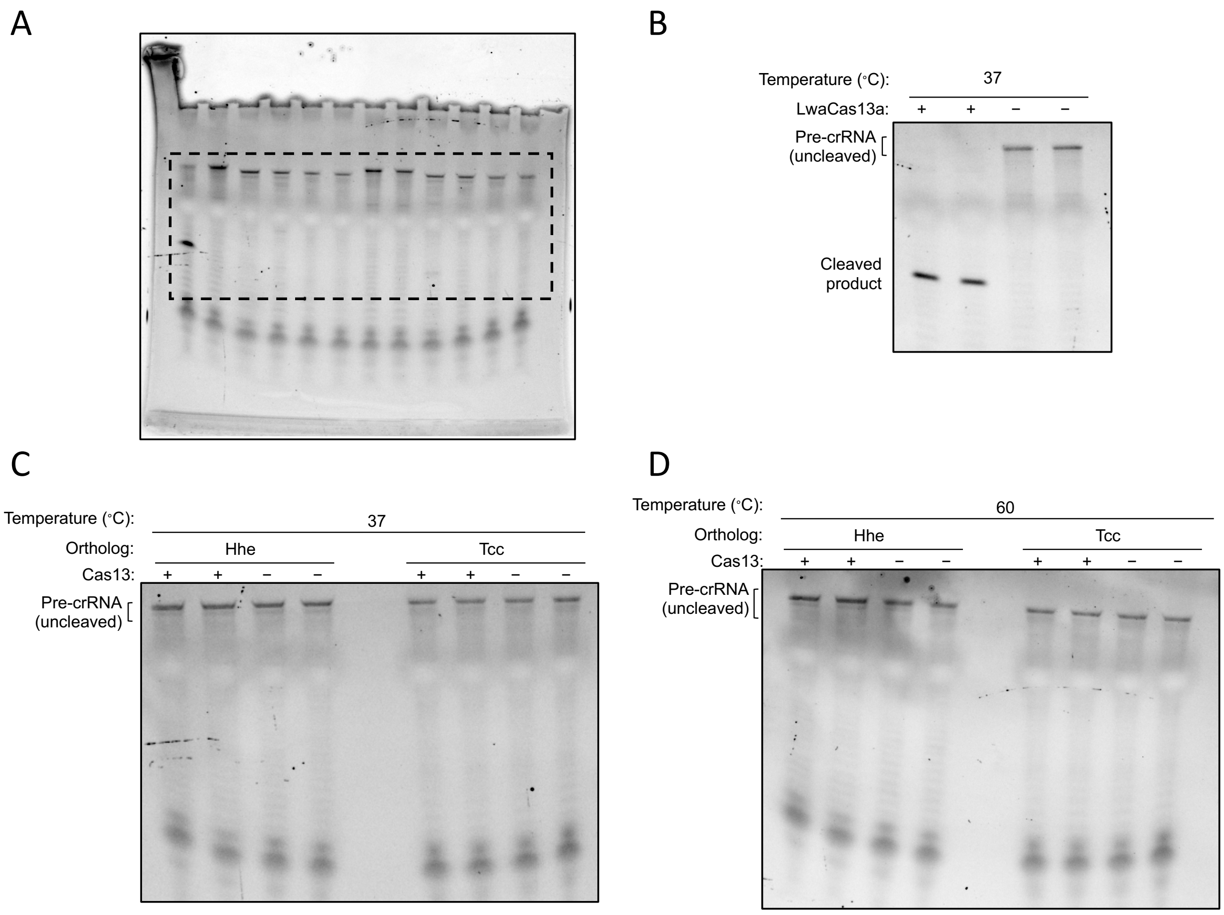


**Supplementary Figure 5: pre-crRNA processing with Cas13a enzymes.**

**A-** Uncropped gel picture of pre-crRNA processing with LwaCas13a, HheCas13a, and TccCas13a proteins related to Figure S3e.

**B-** Representative denaturing gel of 5′-FAM labeled pre-crRNA processing with LwaCas13a.

**C-** Representative denaturing gels of 5′-FAM labeled pre-crRNA processing with HheCas13a and TccCas13a proteins. Reactions were run at 37 °C for 1 hour.

**D-** Representative denaturing gels of 5′-FAM labeled pre-crRNA processing with HheCas13a and TccCas13a proteins. Reactions were run at 60 °C for 1 hour. In all reactions, 200 nM of pre-crRNAs was incubated with 100 nM of Cas13a protein.

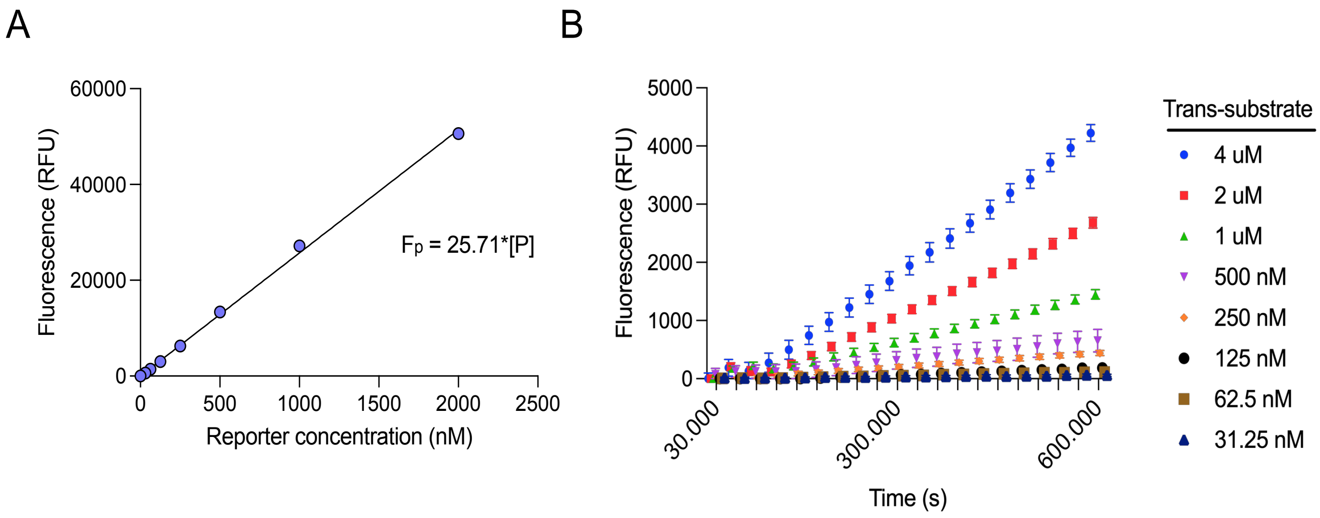


**Supplementary Figure 6: Michaelis-Menten enzyme kinetics assay for TccCas13a.**

**A.** Background-subtracted fluorescence of completely cleaved reporters (Mix reporter-FAM) at concentrations of 31.25 nM, 62.5 nM, 125 nM, 250 nM, 500 nM, 1 µM, and 2 µM was incubated for a prolonged time to ensure complete cleavage. When the reporter is completely cleaved, the concentration of cleaved reporter equals to initial reporter concentration. The data were subtracted with water-only replicates. The solid line is the linear regression that fits experimental data, with equation $F_p = 25.71*[P]$, where $F_p$ is the fluorescence generated by cleaved reporters, and [P] is the concentration of cleaved reporter. Three replicates were done for each reporter concentration.

**B.** Background-subtracted real-time fluorescence signal for *trans*-cleavage kinetics assay. For kinetics assay, 0.5 nM of target-activated RNP was treated with FAM Mix from 31.25 nM to 4 µM (shown detailed on the right side), and the data were subtracted with the data from reactions without adding crRNA. Measurements were carried out every 30 s, and the real-time data from the first 600 s are shown here to generate close-to-linear curves of fluorescence signal over time, whose slopes can be obtained by simple linear regression. The values are shown as mean ± SD (n = 3).

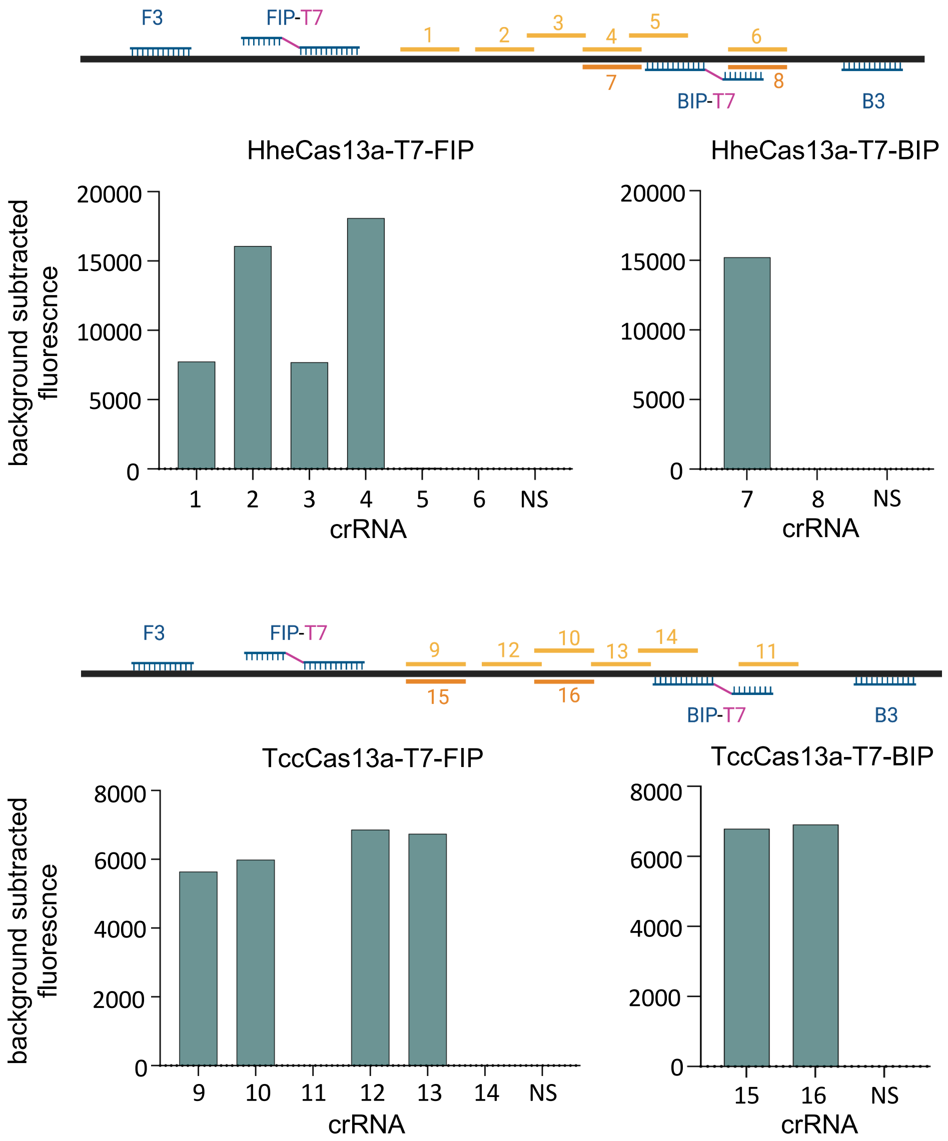


**Supplementary Figure 7: HheCas13a and TccCas13a crRNA screening in two-pot detection reaction.**

Trans cleavage activity of HheCas13a and TccCas13a using different crRNAs when incubated with RT-LAMP product of amplified SARS-CoV-2 genomic standards. Assay was performed as described in material and methods section. Endpoint fluorescence signal detection was measured after 1 hour. NS: non-specific crRNA. T7-FIP: RT-LAMP primers with modified FIP primer carrying T7 promoter sequence. T7-BIP: RT-LAMP primers with modified BIP primer carrying T7 promoter sequence. The location of the targeted sequence of each crRNA (orange) relative to the RT-LAMP primers (F3, FIP-T7, BIP-T7, B3) is depicted on top of each graph. FIP/BIP-T7: primers containing T7 promoter sequence.

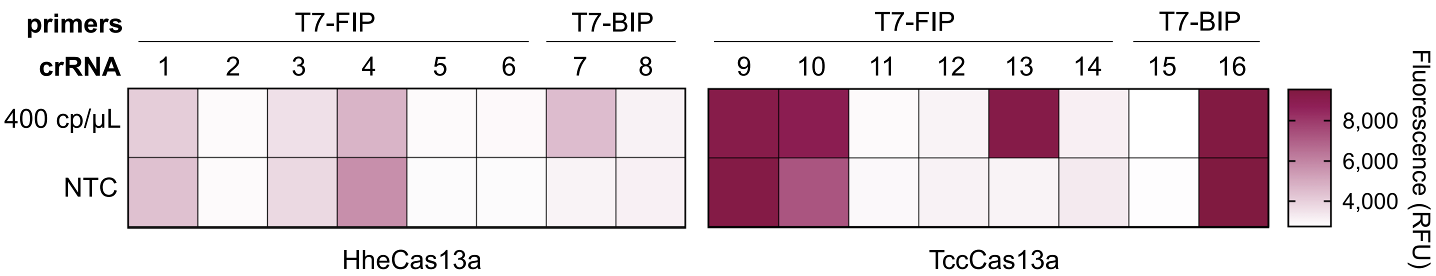


**Supplementary Figure 8: Establishment of one-pot SARS-CoV-2 detection using the thermophilic Cas13 proteins.** *Trans* cleavage activity of HheCas13a and TccCas13a using different crRNAs in one-pot reactions using SARS-CoV-2 genomic standards as input. The assay was performed as described in material and methods. End-point fluorescence signal detection was carried out after 80 min. NTC: No template control. T7-FIP: modified RT-LAMP FIP primer with T7 promoter sequence. T7-BIP: modified RT-LAMP BIP primer with T7 promoter sequence. Data are shown as mean ($n = 3$).

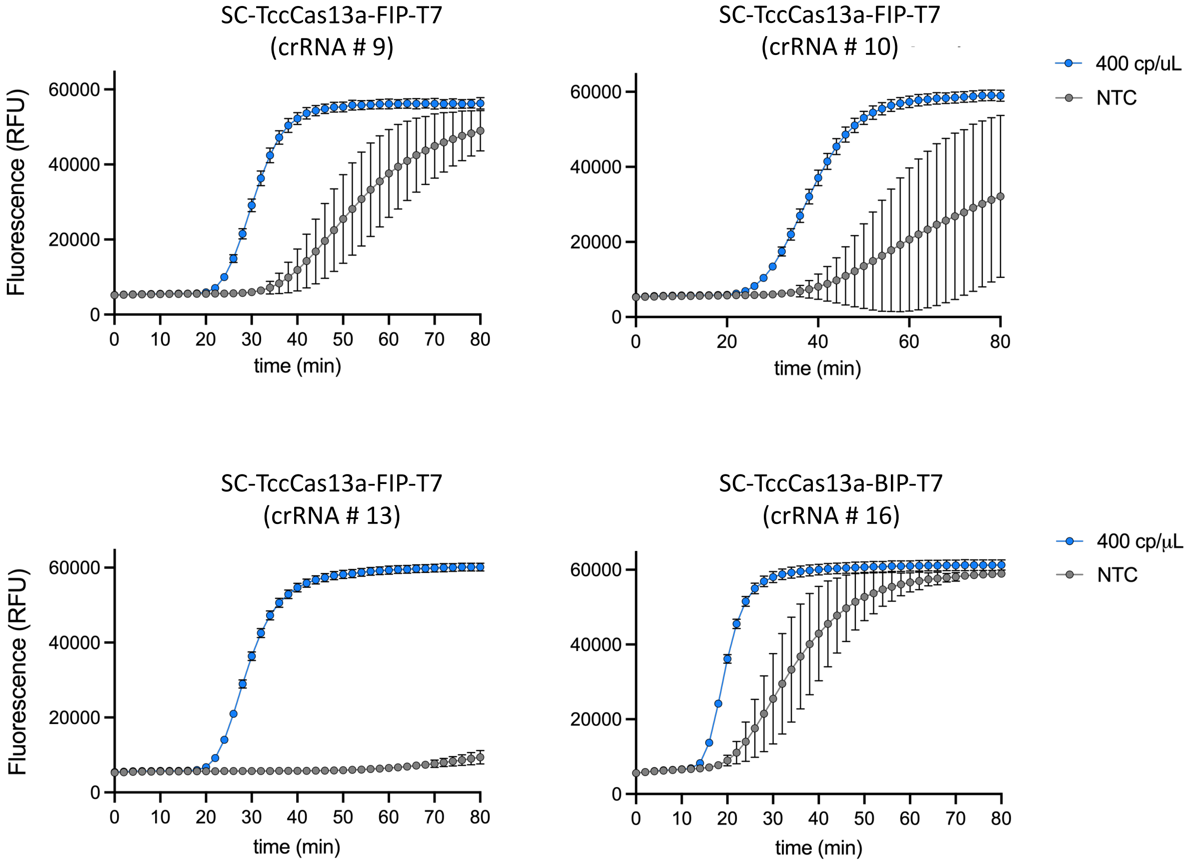


**Supplementary Figure 9: Repeated screening and confirmation of the performance of promising TccCas13a crRNAs for one-pot detection reaction.**

Real time measurements of *trans* cleavage activity of selected TccCas13a crRNAs with the SC primer sets in one-pot reaction using SARS-CoV-2 genomic standards as an input. Assay was performed as described in material and methods section. NTC: no template controls. SC: STOPCovid RT-LAMP primers. T7-FIP: RT-LAMP primers with modified FIP primer with T7 promoter sequence. Data shown as mean ± SD ($n = 3$).

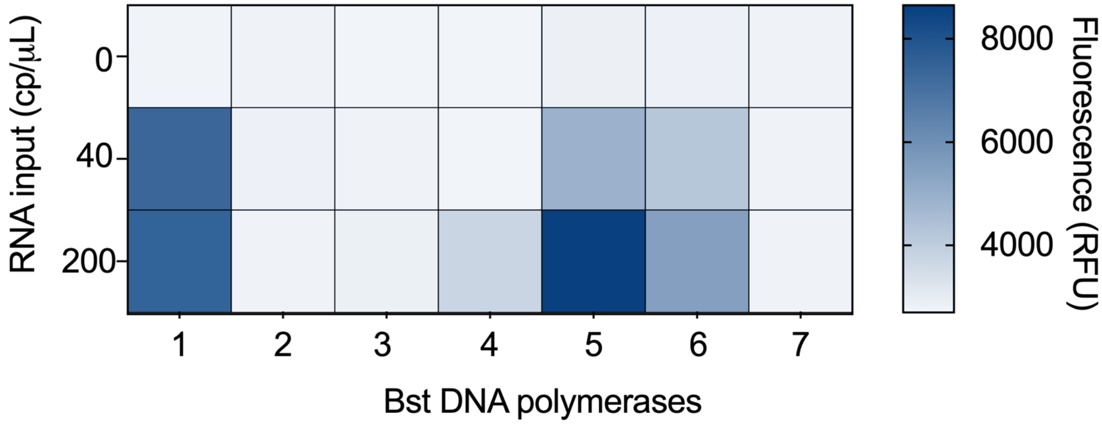


**Supplementary Figure 10: Activity screening of different commercially available *Bst* DNA polymerases for suitability in one-pot assays**. 1- *Bst* DNA polymerase, exonuclease minus (Lucigen, 30028), 2- *Bst* DNA polymerase (web SCIENTIIFIC, S600), 3- *Bst* 2.0 WarmStart DNA polymerase (NEB, M0538), 4- *Bst* 3.0 DNA polymerase (NEB, M0374), 5- *Bsm* DNA polymerase, large fragment (ThermoFisher Scientific, EP0691), 6- *Bst* X DNA polymerase (enzymatics, P7390), 7- WarmStart LAMP kit (DNA&RNA) (NEB, E1700). Data are shown as means ($n = 3$) and represent end-point fluorescence values after 80 min.

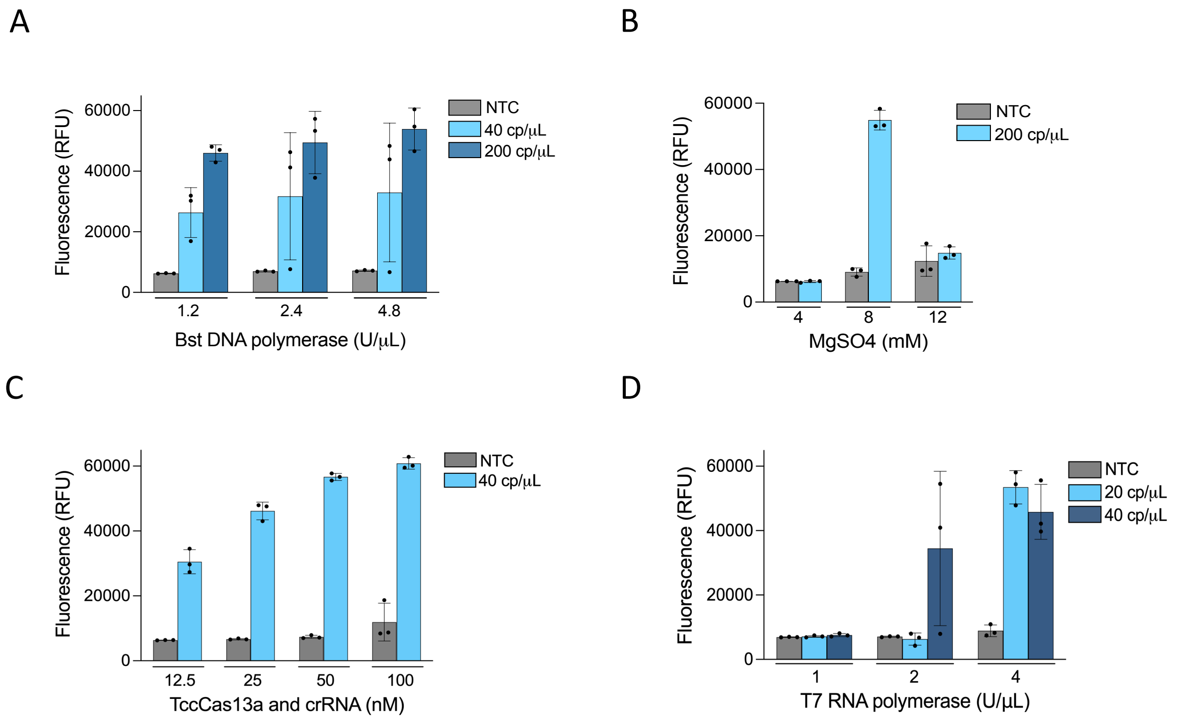


**Supplementary Figure 11: Optimization of one-pot TccCas13a detection reaction.**

A- Optimization of Bst DNA polymerase concentration of the chosen *Bst* DNA polymerase. The 0.38 U/µL concentration of the Bst DNA polymerase was selected. Values are shown as mean ± S.D and represent endpoint fluorescence at 80 mins.

B- Determining the activity of the TccCas13a one-pot detection assay at three different $MgSO_4$ concentrations. A concentration of 8mM showed the best results in the assay. Values are shown as mean ± S.D and represent endpoint fluorescence at 80 mins.

C- Titration of TccCas13a and crRNA concentrations for effects on one-pot detection performance. Values are shown as mean ± S.D and represent endpoint fluorescence at 80 min.

D- Effect of different Hi-T7 RNA polymerase concentrations on the performance of the one-pot detection assay. An evident increase in the performance was observed with 4 U/µL. Values are shown as mean ± S.D and represent endpoint fluorescence at 80 min.

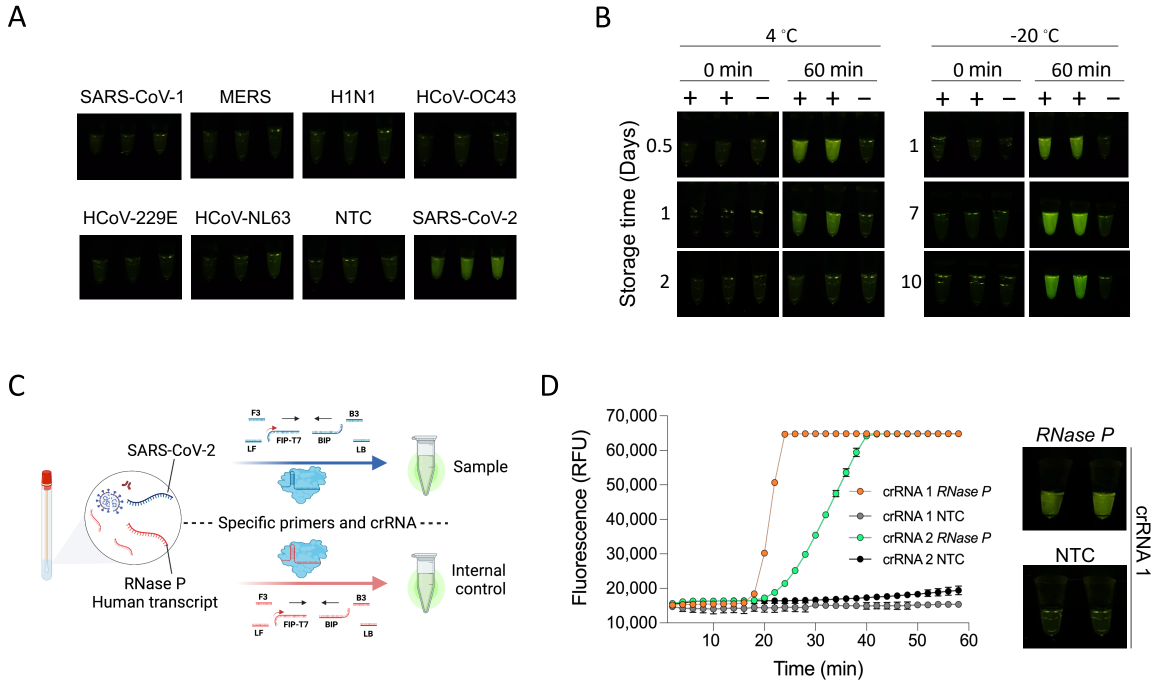


**Supplementary Figure 12: Evaluation of OPTIMA-dx for the detection of SARS-CoV-2.**

**A-** Evaluation of specificity and cross-reactivity of OPTIMA-dx for SARS-CoV-2 RNA visual detection. Three replicates were performed for each treatment. Different synthetic viral genomes were used in this assay including SARS-CoV Control (SARS-CoV-1) (IDT, cat#: 10006624), MERS-CoV Control (IDT, cat#: 10006623), H1N1 (cat#: 103016, Twist Bioscience), HCoV-OC43 (cat#: 103013, Twist Bioscience), and HCoV-229E (cat#: 103011, Twist Bioscience), and HCoV-NL63 (cat#: 103012, Twist Bioscience), NTC: no template control. All synthetic viral genomes were used at concentration of 2000 cp/µL, except SARS-COv-2 that was used at 100 cp/µL.

**B-** Effect of storage time of the OPTIMA-dx master mix at two temperatures on SARS-CoV-2 visual detection. Two replicates and one negative control (NTC: no template control) were tested for each treatment.

**C-** Schematic representation of dual detection of co-isolated and highly abundant human RNase P transcripts with OPTIMA-dx as an internal control for isolated RNA quality and integrity.

**D-** Development and establishment of a human internal control for the OPTIMA-dx assay. Performance of the OPTIMA-dx assay with RNase P-specific LAMP-primers and two crRNAs, as measured by real-time fluorescence (left panel). Data are shown as means ± SD ($n = 3$). The selected crRNA 1 was evaluated for visual detection (right panel) after 60 min incubation. NTC: no template control.

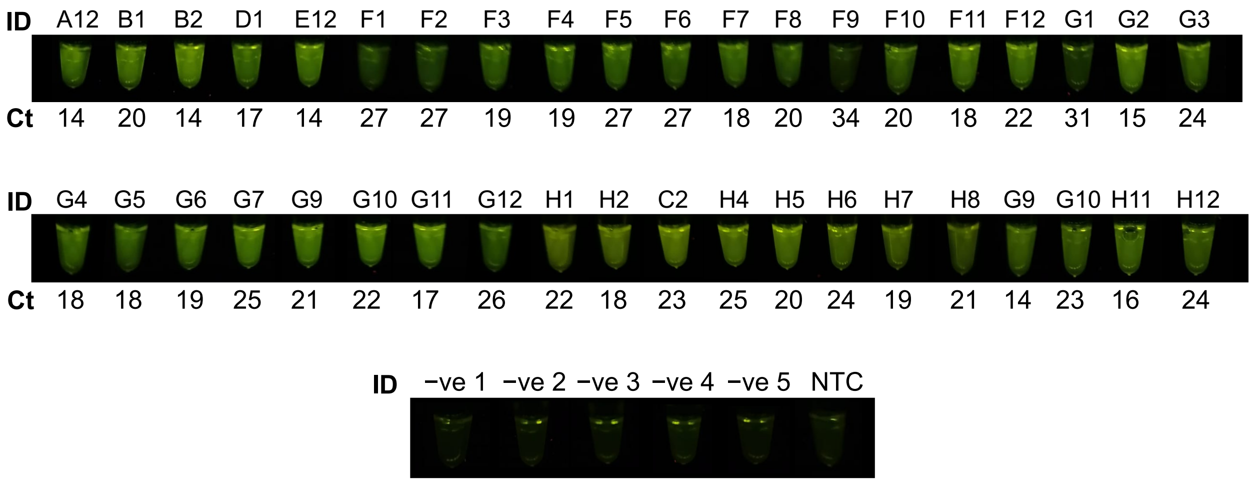


**Supplementary Figure 13: Visual detection of SARS-CoV-2 RNA from samples collected from 45 patients by OPTIMA-dx.**

OPTIMA-dx detection reactions were incubated at 56 °C and endpoint fluorescence signal detection was taken after 1 hour. RT-qPCR Ct values are shown below each sample. -ve: clinical samples negative for SARS-CoV-2. NTC: no template control.

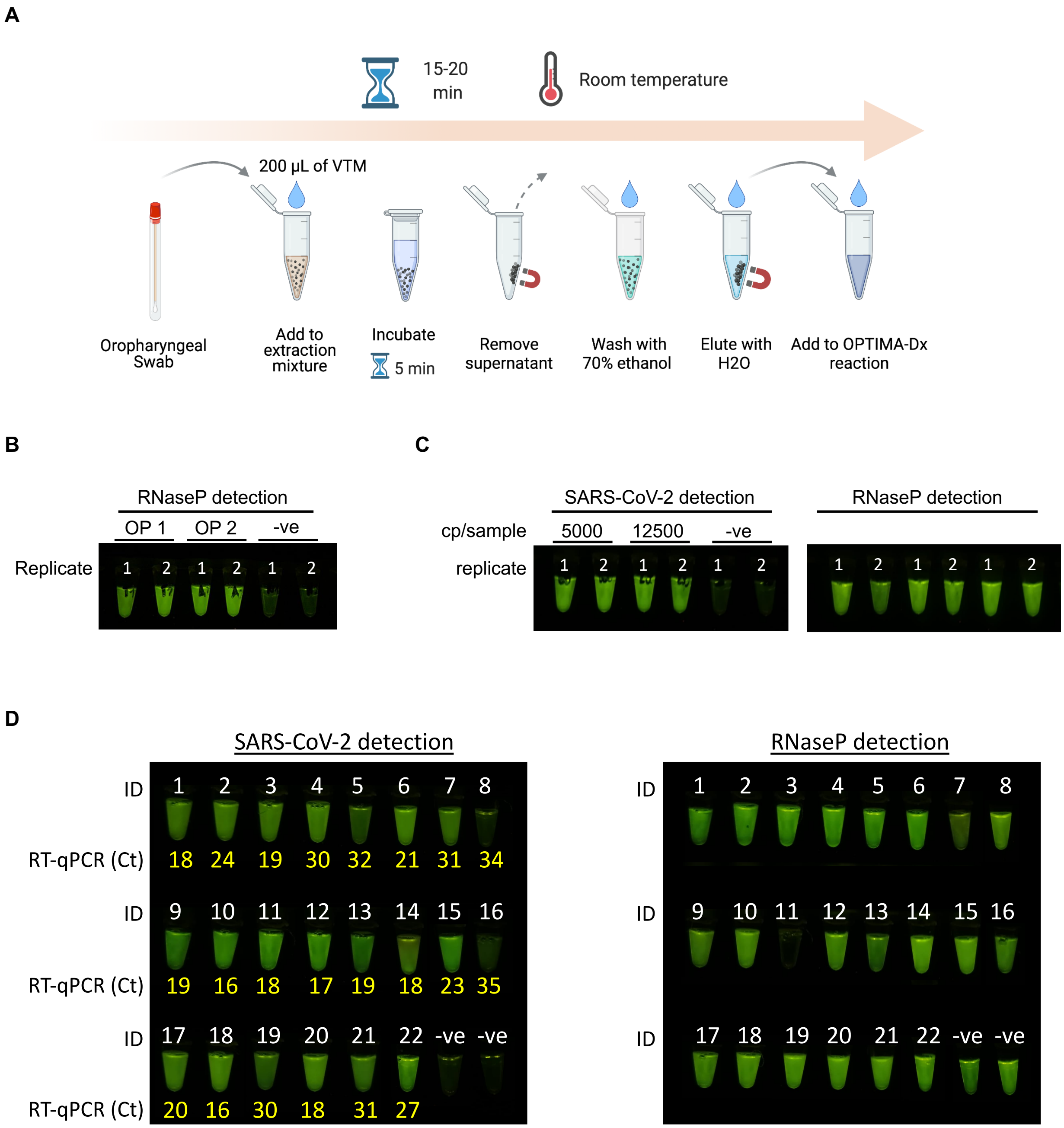


**Supplementary Figure 14: Development and assessment of simple extraction protocol for SARS-CoV-2 detection in unextracted clinical samples.**

A. Schematic outline of the quick extraction protocol employed to process COVID-19 orophyrangial swabs prior to OPTIMA-dx reaction.

B. Validation of quick extraction protocol performance on orophyrangial swabs collected from healthy donors with OPTIMA-dx detection of RNase P. OP: orophyrangial swabs.

C. Validation of quick extraction protocol on orophyrangial swabs collected from healthy donors and spiked with inactivated virus particals with OPTIMA-dx detection of SARS-CoV-2 and RNase P.

D. Detection of SARS-CoV-2 (left panel) and RNase P (right panel) from COVID-19 clinical samples processed with the quick extraction protocol. The eluted RNA from each sample was splitted into two different reactions for the detection of SARS-CoV-2 and RNAseP. OPTIMA-dx detection reactions were incubated at 56 °C and endpoint fluorescence signal detection was taken after 1 hour. In figures B, C, and D, FAM reporter was used at 1 μM final concentration instead of HEX reporters.

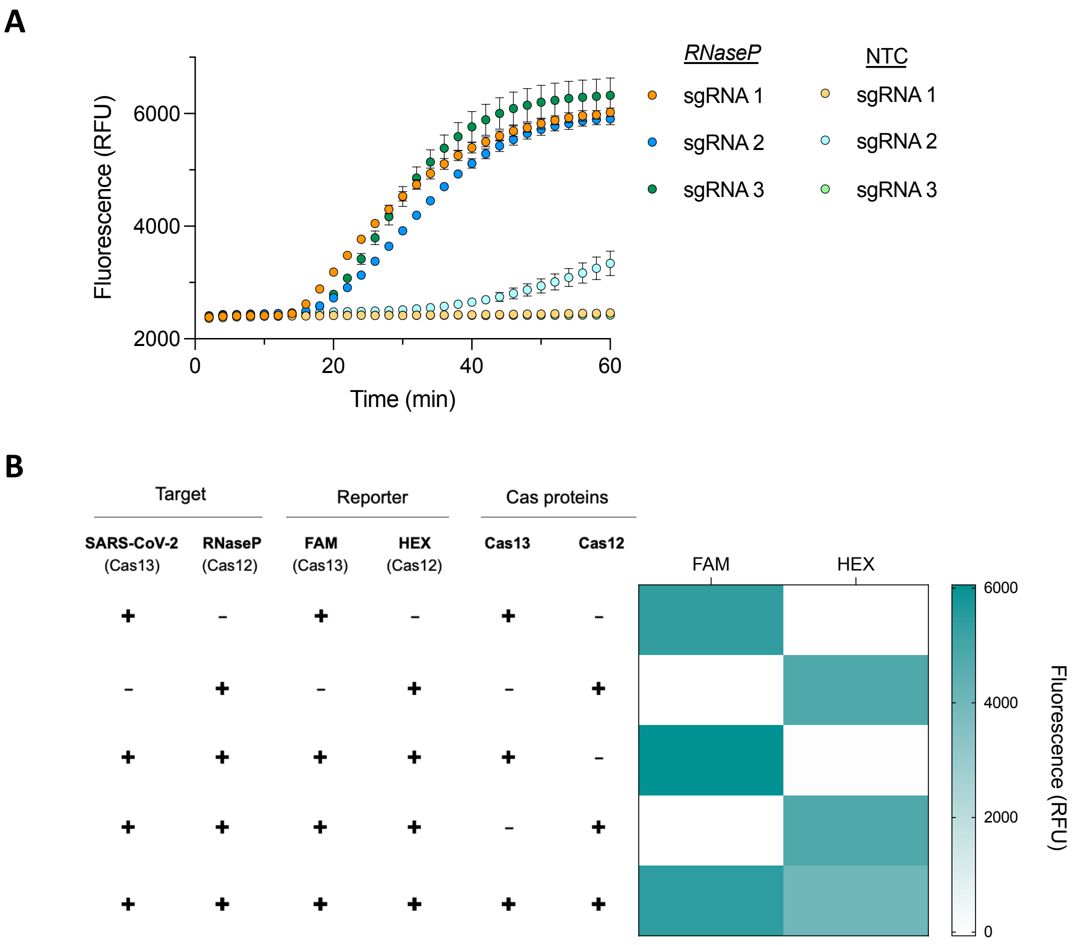


**Supplementary Figure 15: One-pot OPTIMA-dx multiplexed detection**

A. Activity screening of three different AapCas12b sgRNAs in One-pot RT-LAMP Cas12b detection of RNase P template as measured by real-time fluorescence signal produced from HEX reporter cleavage with AapCas12b collateral activity. Data are shown as means ± SD ($n$ = 3). RNase P: total human RNA. NTC: No template control.

B. Analysis of the activity of Cas12 and Cas13 with different reporter molecules and different targets for one-pot OPTIMA-dx multiplex detection. Endpoint fluorescent signal measured after 60 min, values are shown as mean ($n$=3).

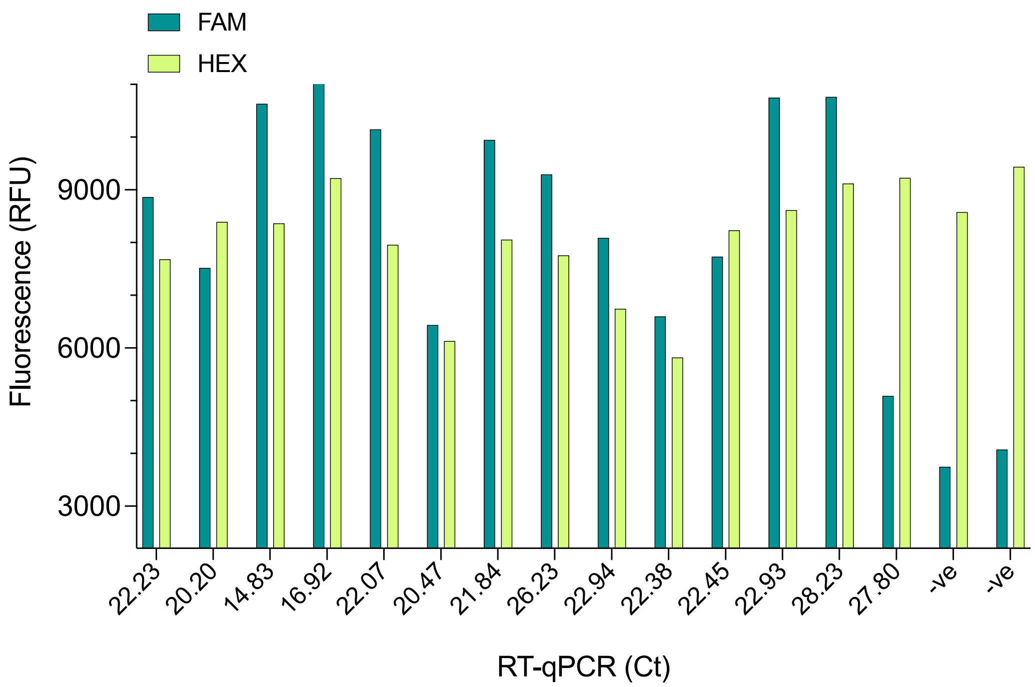


**Supplementary Figure 16: Lyophilization of OPTIMA-dx components.**

Multiplexed detection of SARS-CoV-2 and the human internal control (RNase P) from 16 clinical oropharyngeal swabs processed with the quick extraction method. Detection reactions were incubated at 56°C and the endpoint fluorescent signal was measured with FAM and HEX channels after 2 hours. -Ve: SARS-CoV-2 negative samples as determined with RT-qPCR.

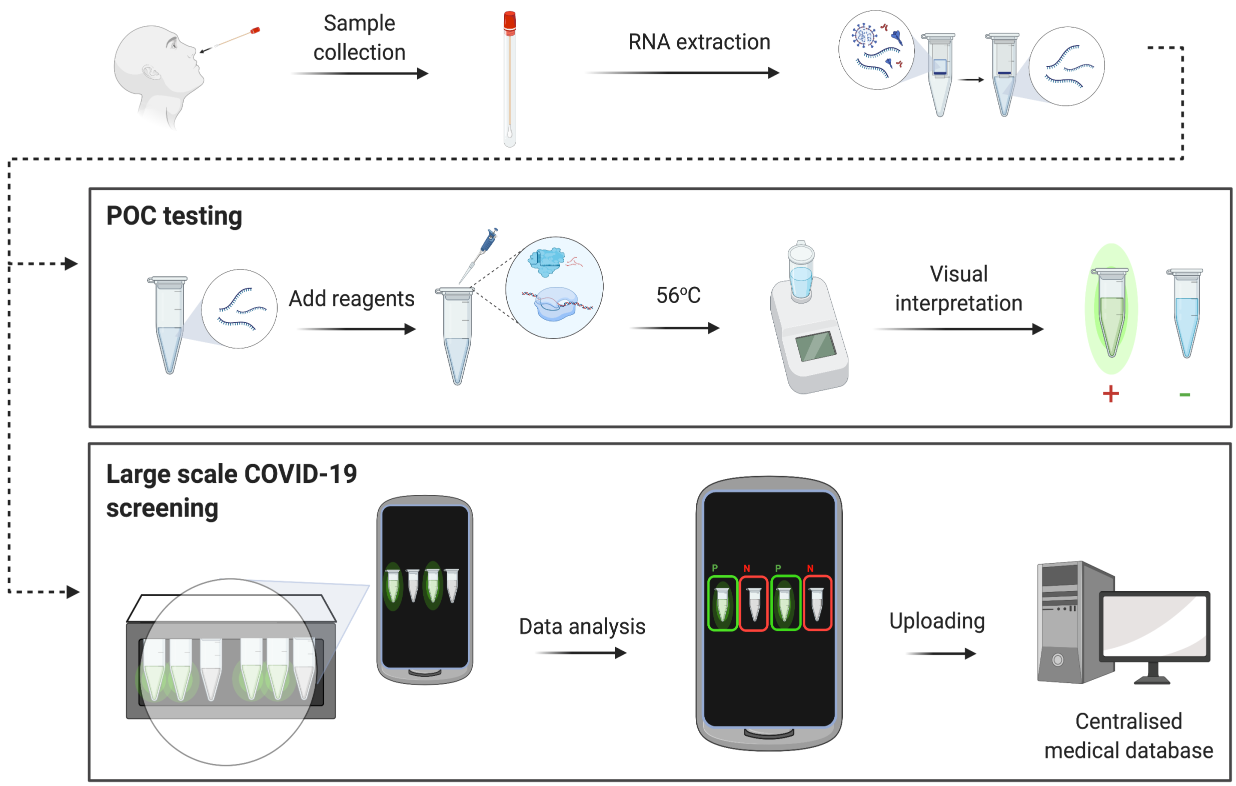


**Supplementary Figure 17: Overview of OPTIMA-dx workflow with mobile phone application for interpreting the OPTIMA-dx fluorescence readouts.** The workflow shows the OPTIMA-dx protocol using RNA extracted from patient sample that is added to the preassembled one-pot reaction. The reaction is incubated at 56°C for 1 h. To interpret the results, the OPTIMA-dx fluorescence readouts are visualized using p51 Molecular Fluorescence Viewer, and the results can be captured using mobile phone camera. The captured picture of the fluorescence readouts is processed with the app and interpreted as positive (P, green) or negative (N, red). The OPTIMA-dx results can be uploaded to or shared with a centralized database.

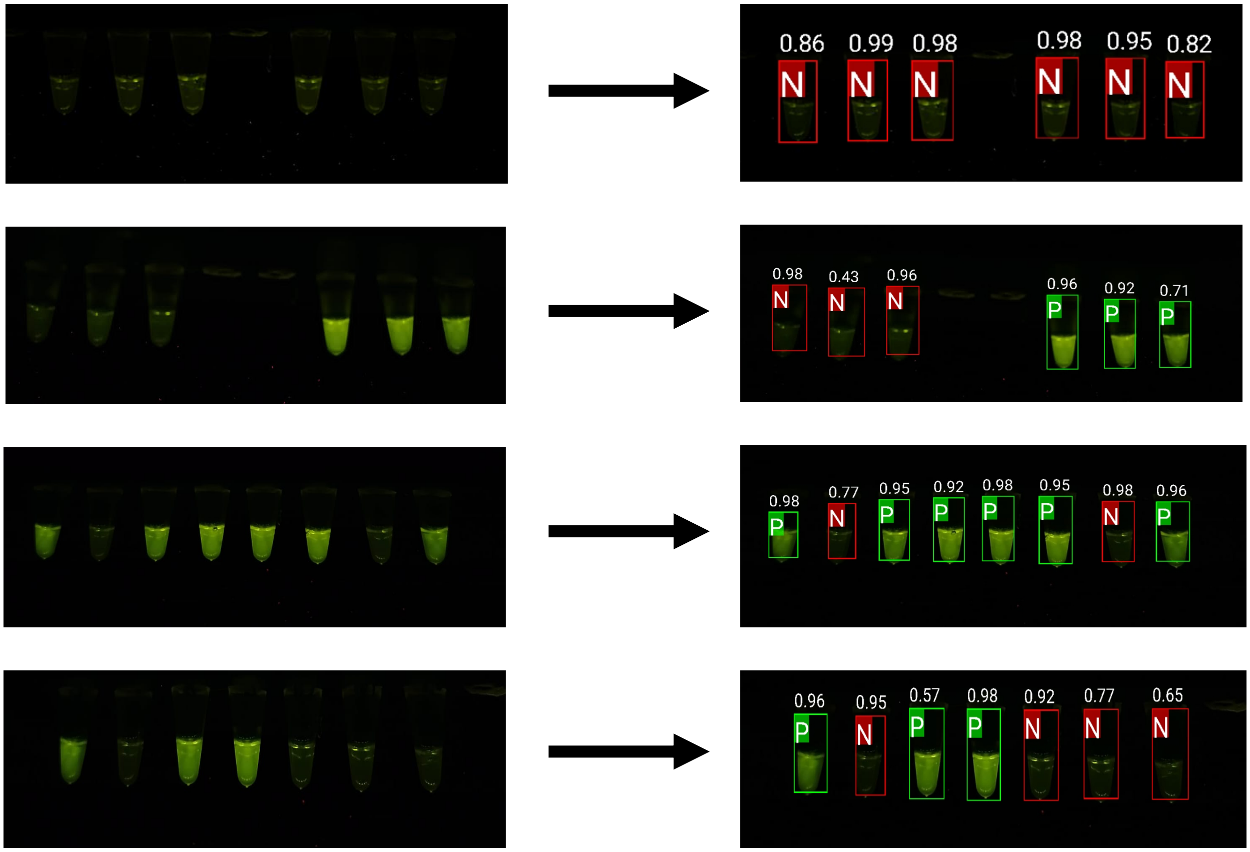


**Supplementary Figure 18: Validation of OPTIMA-dx mobile application for interpretation of fluorescence-based readout results.**

Representative images showing the validation of the OPTIMA-dx application to interpret the visual fluorescent-based readouts of images captured using a smartphone. Images on the left panel represent fluorescence-based visual readout results before OPTIMA-dx app processing. Images on the right panel represent results after OPTIMA-dx app processing. Green squares with "P" indicate positive result. Red squares with "N" indicate negative result.  Confidence scores are shown above the green or red squares.

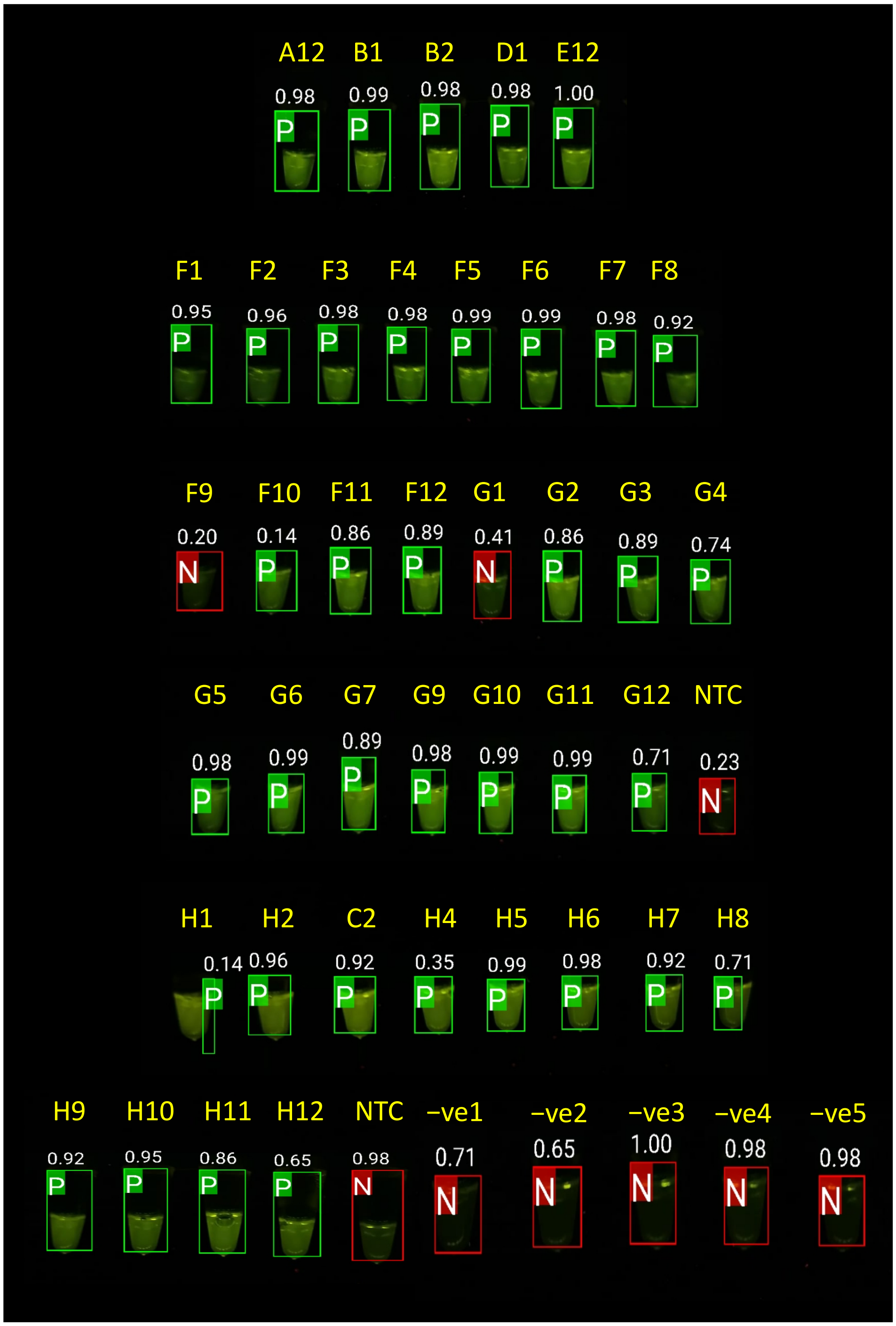


**Supplementary Figure 19: OPTIMA-dx performance on patient samples for detection of SARS-CoV-2.** OPTIMA-dx mobile application tested on patient samples in supplementary figure 13. Images were captured using a smartphone camera with ISO option set to 320 after 60 min of OPTIMA-dx reaction.

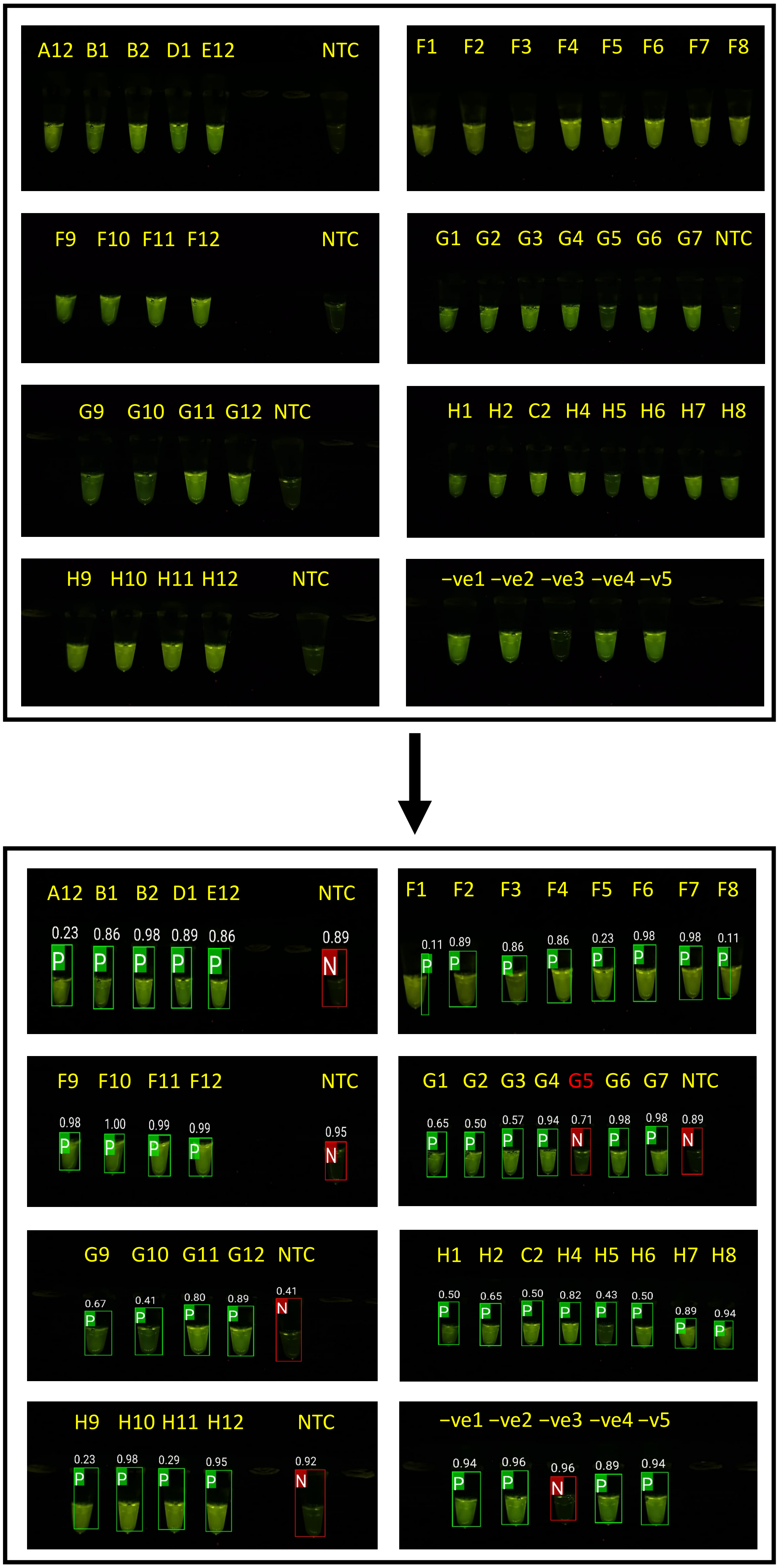
A12 B1 B2 D1 E12 NTC
F1 F2 F3 F4 F5 F6 F7 F8
F9 F10 F11 F12 NTC
G1 G2 G3 G4 G5 G6 G7 NTC
G9 G10 G11 G12 NTC
H1 H2 C2 H4 H5 H6 H7 H8
H9 H10 H11 H12 NTC
−ve1 −ve2 −ve3 −ve4 −v5
A12 B1 B2 D1 E12 NTC
0.23 0.86 0.98 0.89 0.86 0.89
P P P P P N
F1 F2 F3 F4 F5 F6 F7 F8
0.11 0.89 0.86 0.86 0.23 0.98 0.98 0.11
P P P P P P P P
F9 F10 F11 F12 NTC
0.98 1.00 0.99 0.99 0.95
P P P P N
G1 G2 G3 G4 G5 G6 G7 NTC
0.65 0.50 0.57 0.94 0.71 0.98 0.98 0.89
P P P P N P P N
G9 G10 G11 G12 NTC
0.67 0.41 0.80 0.89 0.41
P P P P N
H1 H2 C2 H4 H5 H6 H7 H8
0.50 0.65 0.50 0.82 0.43 0.50 0.89 0.94
P P P P P P P P
H9 H10 H11 H12 NTC
0.23 0.98 0.29 0.95 0.92
P P P P N
−ve1 −ve2 −ve3 −ve4 −v5
0.94 0.96 0.96 0.89 0.94
P P N P P

**Supplementary Figure 20: OPTIMA-dx on patient samples for detection of RNase P human internal control.**

Upper panel, raw images of fluorescent-based visual readouts after 60 min of OPTIMA-dx reaction for the detection of human RNase P transcript from patient samples in in supplementary figure 13. Lower panel, OPTIMA-dx app readouts on samples in upper panel.

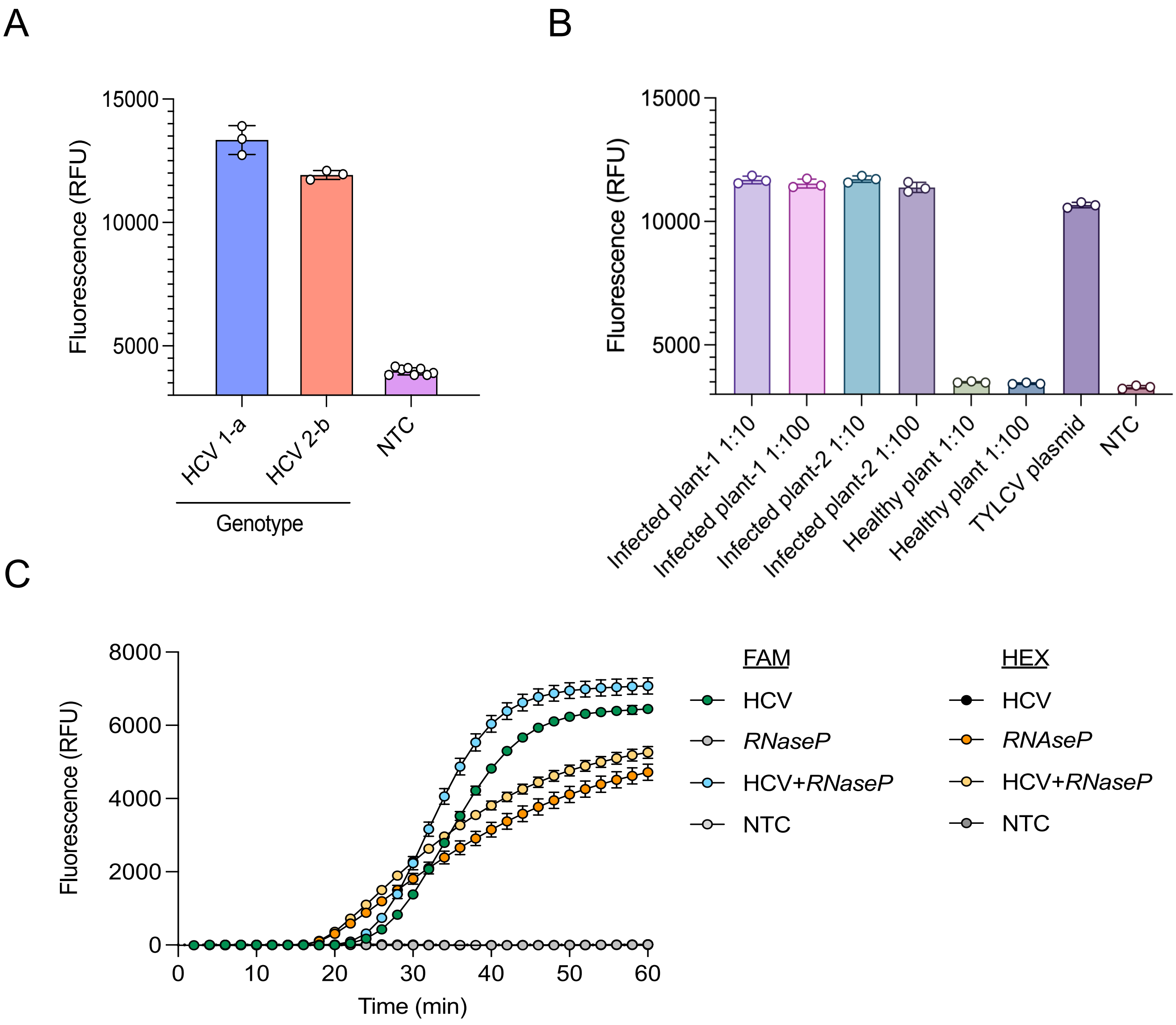


**Supplementary Figure 21: Adaptability of OPTIMA-dx for specific detection of different pathogens.**

A- Detection of major HCV genotypes with OPTIMA-dx. *In vitro* transcribed RNA was used as RNA template in the OPTIMA-dx detection reactions at concentrations of 500 pM. Values are shown as mean ± S.D. and represent endpoint fluorescence at 60 min.

B- Detection of TYLCV DNA virus with OPTIMA-dx. DNA isolated from two different TYLCV infected plants and one healthy (not infected) plant was diluted 1:10 or 1:100 in

water and used as template in the OPTIMA-dx detection reactions. A plasmid containing TYLCV genome was used as a control at concentrations of 1 ng/reaction. NTC: no template control. Values are shown as mean ± S.D and represent endpoint fluorescence at 60 min.

C- Performance of multiplexed detection of HCV at concentrations of 500 pM and isolated human RNA (for *RNAseP* detection) as measured by real-time fluorescence. Data are shown as means ± SD ($n$ = 3).

**Table S1: RT-LAMP primers used in this study.**

| Name | Sequence (5′→3′) | Note |
|---|---|---|
| SC-F3 | GCTGCTGAGGCTTCTAAG | Original and modified STOPCovid primers. Modified part (T7 promoter sequence) is underlined. |
| SC-B3 | GCGTCAATATGCTTATTCAGC | |
| SC-FIP | GCGGCCAATGTTTGTAATCAGTAGACGTGGTCCAGAACAA | |
| SC-BIP | TCAGCGTTCTTCGGAATGTCGCTGTGTAGGTCAACCACG | |
| SC-LF | CCTTGTCTGATTAGTTCCTGGT | |
| SC-LB | TGGCATGGAAGTCACACC | |
| SC-T7-FIP | GCGGCCAATGTTTGTAATCAGTTAATACGACTCACTATAGGGAGACGTGGTCCAGAACAA | |
| SC-T7-BIP | TCAGCGTTCTTCGGAATGTCGCTAATACGACTCACTATAGGGTGTGTAGGTCAACCACG | |
| RNase P-F3 | TTGATGAGCTGGAGCCA | Original and modified RNase P POP primers. Modified part (T7 promoter sequence) is underlined. |
| RNase P-B3 | CACCCTCAATGCAGAGTC | |
| RNase P-FIP | GTGTGACCCTGAAGACTCGGTTTTAGCCACTGACTCGGATC | |
| RNase P-BIP | CCTCCGTGATATGGCTCTTCGTTTTTTTCTTACATGGCTCTGGTC | |
| RNase P-LF | ATGTGGATGGCTGAGTTGTT | |
| RNase P-LB | CATGCTGAGTACTGGACCTC | |
| RNase P-T7-FIP | GTGTGACCCTGAAGACTCGGTTTTTAATACGACTCACTATAGGGAGCCACTGACTCGGATC | |

| | | |
|---|---|---|
| HCV-F3 | TGTCTTCACGCAGAAAGCG | HCV RT-LAMP primers. Modified part (T7 promoter sequence) is underlined. |
| HCV-B3 | TACCACAAGGCCTTTCGC | |
| HCV-T7-FIP | TCCGCAGACCACTATGGCTCTCTAATACGACTCACTATAGGGCCATGGCGTTAGTATGAGT | |
| HCV-BIP | AGGACGACCGGGTCCTTTCTACTACTCGGCTAGCAGTCTT | |
| HCV-LF | GGTCCTGGAGGCTGCACGAC | |
| HCV-LB | GAGATTTGGGCGTGCCCCCGC | |
| TYLCV-F3 | GGTAAAGTCTGGATGGATGA | TYLCV LAMP primers. Modified part (T7 promoter sequence) is underlined.<br>-Original primers [7]. |
| TYLCV-B3 | TGTTCCTTCATTCCAGAGG | |
| TYLCV-T7-FIP | ACCTGTCCAAAATCCATTGTAATACGACTCACTATAGGGCAGAATCACACTAATCAGGTC | |
| TYLCV-BIP | CAGTACCGCAACCGTGAAGACAATAACTGTAGCATGAAATTTCCT | |
| TYLCV-LF | CTATCACGGACCAAGAAGAAC | |
| TYLCV-LB | GATTTGCGGGATAGGTTTCAAG | |

**Table S2: crRNA sequences used in this study.**

| crRNA # | crRNA Name | **crRNA sequence**<br>crRNA sequences shown as 5`→3` reverse complement to be annealed with **T7 oligo** for *in vitro* transcription | crRNA ID in the figures (#) |
|---|---|---|---|
| 1101 | Hhe-Sense-28-1 | ATCAGACAAGGAACTGATTACAAACATTG TTGCAGTTCCCCTGTCTACGGGGATTGTTA CCCTATAGTGAGTCGTATTAATTTC | Supp. Figure 7, 8 (#1) |
| 1102 | Hhe-Sense-28-2 | CCGCAAATTGCACAATTTGCCCCCAGCGG TTGCAGTTCCCCTGTCTACGGGGATTGTTA CCCTATAGTGAGTCGTATTAATTTC | -Figure 2A, C, D, E (#1)<br>-Supp. Figure 3 (#1)<br>-Supp. Figure 7, 8 (#2) |
| 1103 | Hhe-Sense-28-3 | CGCTTCAGCGTTCTTCGGAATGTCGCGCGT TGCAGTTCCCCTGTCTACGGGGATTGTTAC CCTATAGTGAGTCGTATTAATTTC | Supp. Figure 7, 8 (#3) |
| 1104 | Hhe-Sense-28-4 | CATTGGCATGGAAGTCACACCTTCGGGAG TTGCAGTTCCCCTGTCTACGGGGATTGTTA CCCTATAGTGAGTCGTATTAATTTC | -Figure 2A, E (#2)<br>-Supp. Figure 3 (#2)<br>-Supp. Figure 7, 8 (#4) |
| 1105 | Hhe-Sense-28-5 | GAACGTGGTTGACCTACACAGGTGCCATG TTGCAGTTCCCCTGTCTACGGGGATTGTTA CCCTATAGTGAGTCGTATTAATTTC | Supp. Figure 7, 8 (#5) |
| 1106 | Hhe-Sense-28-6 | ATCAAATTGGATGACAAAGATCCAAATTG TTGCAGTTCCCCTGTCTACGGGGATTGTTA CCCTATAGTGAGTCGTATTAATTTC | Supp. Figure 7, 8 (#6) |
| NS | Hhe-NS | ctccgtgatatggctcttcgcatgctgaGTTGCAGTTCCCC TGTCTACGGGGATTGTTACCCTATAGTGAG TCGTATTAATTTC | -Figure 2A, C, E (#NS)<br>-Supp. Figure 3 (#NS)<br>-Supp. Figure 7, 8 (#NS) |
| 1087 | Hhe-AntiSense-28-4 | TCCCGAAGGTGTGACTTCCATGCCAATGGT TGCAGTTCCCCTGTCTACGGGGATTGTTAC CCTATAGTGAGTCGTATTAATTTC | Supp. Figure 7, 8 (#7) |

| | | | |
|---|---|---|---|
| 1088 | Hhe-AntiSense-28-6 | AATTTGGATCTTTGTCATCCAATTTGATGTTGCAGTTCCCCTGTCTACGGGGATTGTTACCCTATAGTGAGTCGTATTAATTTC | Supp. Figure 7, 8 (#8) |
| 1125 | Tcc-Sense-24-1 | CAGACAAGGAACTGATTACAAACAGTTGCAGTCTCCGCCTACATGGGAGTTGTGACCCTATAGTGAGTCGTATTAATTTC | Supp. Figure 7, 8 (#9) |
| 1126 | Tcc-Sense-24-3 | CTTCAGCGTTCTTCGGAATGTCGCGTTGCAGTCTCCGCCTACATGGGAGTTGTGACCCTATAGTGAGTCGTATTAATTTC | Supp. Figure 7, 8 (#10) |
| 1127 | Tcc-Sense-24-6 | CAAATTGGATGACAAAGATCCAAAGTTGCAGTCTCCGCCTACATGGGAGTTGTGACCCTATAGTGAGTCGTATTAATTTC | Supp. Figure 7, 8 (#11) |
| 1129 | Tcc-Sense-28-3 | CGCTTCAGCGTTCTTCGGAATGTCGCGCGTTGCAGTCTCCGCCTACATGGGAGTTGTGACCCTATAGTGAGTCGTATTAATTTC | Supp. Figure 1 (#1) |
| 1130 | Tcc-Sense-28-6 | ATCAAATTGGATGACAAAGATCCAAATTGTTGCAGTCTCCGCCTACATGGGAGTTGTGACCCTATAGTGAGTCGTATTAATTTC | Supp. Figure 1 (#2) |
| 1171 | Tcc-Sense-24-2 | GCAAATTGCACAATTTGCCCCCAGGTTGCAGTCTCCGCCTACATGGGAGTTGTGACCCTATAGTGAGTCGTATTAATTTC | -Figure 2A, B, C, D, E (#1)<br>-Supp. Figure 7, 8 (#12) |
| 1172 | Tcc-Sense-24-4 | TTGGCATGGAAGTCACACCTTCGGGTTGCAGTCTCCGCCTACATGGGAGTTGTGACCCTATAGTGAGTCGTATTAATTTC | -Figure 2A, B, E (#2)<br>- Figure 3F<br>- Supp. Figure 7, 8 (#13)<br>**The selected crRNA for SARS-CoV-2 detection** |
| 1173 | Tcc-Sense-24-5 | ACGTGGTTGACCTACACAGGTGCCGTTGCAGTCTCCGCCTACATGGGAGTTGTGACCCTATAGTGAGTCGTATTAATTTC | Supp. Figure 7, 8 (#14) |
| NS | Tcc-NS | actcagccatccacatccgagtcttcagGTTGCAGTCTCCGCCTACATGGGAGTTGTGACCCTATAGTGAGTCGTATTAATTTC | -Figure 2A, B, C, E (#NS)<br>- Supp. Figure 7, 8 (#NS) |

| | | | |
|---|---|---|---|
| | | | |
| 1174 | Tcc-AntiSense-24-1 | TGTTTGTAATCAGTTCCTTGTCTGGTTGCAGTCTCCGCCTACATGGGAGTTGTGACCCTATAGTGAGTCGTATTAATTTC | Supp. Figure 7, 8 (#15) |
| 1175 | Tcc-AntiSense-24-3 | GCGACATTCCGAAGAACGCTGAAGGTTGCAGTCTCCGCCTACATGGGAGTTGTGACCCTATAGTGAGTCGTATTAATTTC | Supp. Figure 7, 8 (#16) |
| 1243 | Tcc-RNase P-24-1 | TCAGCCATCCACATCCGAGTCTTCGTTGCAGTCTCCGCCTACATGGGAGTTGTGACCCTATAGTGAGTCGTATTAATTTC | Figure 5F (crRNA1) **Selected crRNA for RNase P detection** |
| 1244 | Tcc-RNase P-24-2 | CCGTGATATGGCTCTTCGCATGCTGTTGCAGTCTCCGCCTACATGGGAGTTGTGACCCTATAGTGAGTCGTATTAATTTC | Figure 5F (crRNA2) |
| 1246 | 1104-Mis 1-1 | GATTGGCATGGAAGTCACACCTTCGGGAGTTGCAGTTCCCCTGTCTACGGGGATTGTTACCCTATAGTGAGTCGTATTAATTTC | Supp. Figure 4 |
| 1247 | 1104-Mis 1-2 | CTTTGGCATGGAAGTCACACCTTCGGGAGTTGCAGTTCCCCTGTCTACGGGGATTGTTACCCTATAGTGAGTCGTATTAATTTC | Supp. Figure 4 |
| 1248 | 1104-Mis 1-3 | CAATGGCATGGAAGTCACACCTTCGGGAGTTGCAGTTCCCCTGTCTACGGGGATTGTTACCCTATAGTGAGTCGTATTAATTTC | Supp. Figure 4 |
| 1249 | 1104-Mis 1-4 | CATAGGCATGGAAGTCACACCTTCGGGAGTTGCAGTTCCCCTGTCTACGGGGATTGTTACCCTATAGTGAGTCGTATTAATTTC | Supp. Figure 4 |
| 1250 | 1104-Mis 1-5 | CATTCGCATGGAAGTCACACCTTCGGGAGTTGCAGTTCCCCTGTCTACGGGGATTGTTACCCTATAGTGAGTCGTATTAATTTC | Supp. Figure 4 |
| 1251 | 1104-Mis 1-6 | CATTGCCATGGAAGTCACACCTTCGGGAGTTGCAGTTCCCCTGTCTACGGGGATTGTTACCCTATAGTGAGTCGTATTAATTTC | Supp. Figure 4 |
| 1252 | 1104-Mis 1-7 | CATTGGGATGGAAGTCACACCTTCGGGAGTTGCAGTTCCCCTGTCTACGGGGATTGTTACCCTATAGTGAGTCGTATTAATTTC | Supp. Figure 4 |
| 1253 | 1104-Mis 1-8 | CATTGGCTTGGAAGTCACACCTTCGGGAGTTGCAGTTCCCCTGTCTACGGGGATTGTTACCCTATAGTGAGTCGTATTAATTTC | Supp. Figure 4 |

| | | | |
|---|---|---|---|
| 1254 | 1104-Mis 1-9 | CATTGGCAAGGAAGTCACACCTTCGGGAG TTGCAGTTCCCCTGTCTACGGGGATTGTTA CCCTATAGTGAGTCGTATTAATTTC | Supp. Figure 4 |
| 1255 | 1104-Mis 1-10 | CATTGGCATCGAAGTCACACCTTCGGGAG TTGCAGTTCCCCTGTCTACGGGGATTGTTA CCCTATAGTGAGTCGTATTAATTTC | Supp. Figure 4 |
| 1256 | 1104-Mis 1-11 | CATTGGCATGCAAGTCACACCTTCGGGAG TTGCAGTTCCCCTGTCTACGGGGATTGTTA CCCTATAGTGAGTCGTATTAATTTC | Supp. Figure 4 |
| 1257 | 1104-Mis 1-12 | CATTGGCATGGTAGTCACACCTTCGGGAG TTGCAGTTCCCCTGTCTACGGGGATTGTTA CCCTATAGTGAGTCGTATTAATTTC | Supp. Figure 4 |
| 1258 | 1104-Mis 1-13 | CATTGGCATGGATGTCACACCTTCGGGAG TTGCAGTTCCCCTGTCTACGGGGATTGTTA CCCTATAGTGAGTCGTATTAATTTC | Supp. Figure 4 |
| 1259 | 1104-Mis 1-14 | CATTGGCATGGAACTCACACCTTCGGGAG TTGCAGTTCCCCTGTCTACGGGGATTGTTA CCCTATAGTGAGTCGTATTAATTTC | Supp. Figure 4 |
| 1260 | 1104-Mis 1-15 | CATTGGCATGGAAGACACACCTTCGGGAG TTGCAGTTCCCCTGTCTACGGGGATTGTTA CCCTATAGTGAGTCGTATTAATTTC | Supp. Figure 4 |
| 1261 | 1104-Mis 1-16 | CATTGGCATGGAAGTGACACCTTCGGGAG TTGCAGTTCCCCTGTCTACGGGGATTGTTA CCCTATAGTGAGTCGTATTAATTTC | Supp. Figure 4 |
| 1262 | 1104-Mis 1-17 | CATTGGCATGGAAGTCTCACCTTCGGGAG TTGCAGTTCCCCTGTCTACGGGGATTGTTA CCCTATAGTGAGTCGTATTAATTTC | Supp. Figure 4 |
| 1263 | 1104-Mis 1-18 | CATTGGCATGGAAGTCAGACCTTCGGGAG TTGCAGTTCCCCTGTCTACGGGGATTGTTA CCCTATAGTGAGTCGTATTAATTTC | Supp. Figure 4 |
| 1264 | 1104-Mis 1-19 | CATTGGCATGGAAGTCACTCCTTCGGGAG TTGCAGTTCCCCTGTCTACGGGGATTGTTA CCCTATAGTGAGTCGTATTAATTTC | Supp. Figure 4 |
| 1265 | 1104-Mis 1-20 | CATTGGCATGGAAGTCACAGCTTCGGGAG TTGCAGTTCCCCTGTCTACGGGGATTGTTA CCCTATAGTGAGTCGTATTAATTTC | Supp. Figure 4 |
| 1266 | 1104-Mis 1-21 | CATTGGCATGGAAGTCACACGTTCGGGAG TTGCAGTTCCCCTGTCTACGGGGATTGTTA CCCTATAGTGAGTCGTATTAATTTC | Supp. Figure 4 |
| 1267 | 1104-Mis 1-22 | CATTGGCATGGAAGTCACACCATCGGGAG TTGCAGTTCCCCTGTCTACGGGGATTGTTA CCCTATAGTGAGTCGTATTAATTTC | Supp. Figure 4 |

| | | | |
|---|---|---|---|
| 1268 | 1104-Mis 1-23 | CATTGGCATGGAAGTCACACCTACGGGAG TTGCAGTTCCCCTGTCTACGGGGATTGTTA CCCTATAGTGAGTCGTATTAATTTC | Supp. Figure 4 |
| 1269 | 1104-Mis 1-24 | CATTGGCATGGAAGTCACACCTTGGGGAG TTGCAGTTCCCCTGTCTACGGGGATTGTTA CCCTATAGTGAGTCGTATTAATTTC | Supp. Figure 4 |
| 1270 | 1104-Mis 1-25 | CATTGGCATGGAAGTCACACCTTCCGGAG TTGCAGTTCCCCTGTCTACGGGGATTGTTA CCCTATAGTGAGTCGTATTAATTTC | Supp. Figure 4 |
| 1271 | 1104-Mis 1-26 | CATTGGCATGGAAGTCACACCTTCGCGAG TTGCAGTTCCCCTGTCTACGGGGATTGTTA CCCTATAGTGAGTCGTATTAATTTC | Supp. Figure 4 |
| 1272 | 1104-Mis 1-27 | CATTGGCATGGAAGTCACACCTTCGGCAG TTGCAGTTCCCCTGTCTACGGGGATTGTTA CCCTATAGTGAGTCGTATTAATTTC | Supp. Figure 4 |
| 1273 | 1104-Mis 1-28 | CATTGGCATGGAAGTCACACCTTCGGGTG TTGCAGTTCCCCTGTCTACGGGGATTGTTA CCCTATAGTGAGTCGTATTAATTTC | Supp. Figure 4 |
| 1274 | 1104-Mis-2-1 | GTTTGGCATGGAAGTCACACCTTCGGGAG TTGCAGTTCCCCTGTCTACGGGGATTGTTA CCCTATAGTGAGTCGTATTAATTTC | Supp. Figure 4 |
| 1275 | 1104-Mis-2-2 | CAAAGGCATGGAAGTCACACCTTCGGGAG TTGCAGTTCCCCTGTCTACGGGGATTGTTA CCCTATAGTGAGTCGTATTAATTTC | Supp. Figure 4 |
| 1276 | 1104-Mis-2-3 | CATTCCCATGGAAGTCACACCTTCGGGAG TTGCAGTTCCCCTGTCTACGGGGATTGTTA CCCTATAGTGAGTCGTATTAATTTC | Supp. Figure 4 |
| 1277 | 1104-Mis-2-4 | CATTGGGTTGGAAGTCACACCTTCGGGAG TTGCAGTTCCCCTGTCTACGGGGATTGTTA CCCTATAGTGAGTCGTATTAATTTC | Supp. Figure 4 |
| 1278 | 1104-Mis-2-5 | CATTGGCAACGAAGTCACACCTTCGGGAG TTGCAGTTCCCCTGTCTACGGGGATTGTTA CCCTATAGTGAGTCGTATTAATTTC | Supp. Figure 4 |
| 1279 | 1104-Mis-2-6 | CATTGGCATGCTAGTCACACCTTCGGGAGT TGCAGTTCCCCTGTCTACGGGGATTGTTAC CCTATAGTGAGTCGTATTAATTTC | Supp. Figure 4 |
| 1280 | 1104-Mis-2-7 | CATTGGCATGGATCTCACACCTTCGGGAGT TGCAGTTCCCCTGTCTACGGGGATTGTTAC CCTATAGTGAGTCGTATTAATTTC | Supp. Figure 4 |
| 1281 | 1104-Mis-2-8 | CATTGGCATGGAAGAGACACCTTCGGGAG TTGCAGTTCCCCTGTCTACGGGGATTGTTA CCCTATAGTGAGTCGTATTAATTTC | Supp. Figure 4 |

| | | | |
|---|---|---|---|
| 1282 | 1104-Mis-2-9 | CATTGGCATGGAAGTCTGACCTTCGGGAG TTGCAGTTCCCCTGTCTACGGGGATTGTTA CCCTATAGTGAGTCGTATTAATTTC | Supp. Figure 4 |
| 1283 | 1104-Mis-2-10 | CATTGGCATGGAAGTCACTGCTTCGGGAG TTGCAGTTCCCCTGTCTACGGGGATTGTTA CCCTATAGTGAGTCGTATTAATTTC | Supp. Figure 4 |
| 1284 | 1104-Mis-2-11 | CATTGGCATGGAAGTCACACGATCGGGAG TTGCAGTTCCCCTGTCTACGGGGATTGTTA CCCTATAGTGAGTCGTATTAATTTC | Supp. Figure 4 |
| 1285 | 1104-Mis-2-12 | CATTGGCATGGAAGTCACACCTAGGGGAG TTGCAGTTCCCCTGTCTACGGGGATTGTTA CCCTATAGTGAGTCGTATTAATTTC | Supp. Figure 4 |
| 1286 | 1104-Mis-2-13 | CATTGGCATGGAAGTCACACCTTCCCGAG TTGCAGTTCCCCTGTCTACGGGGATTGTTA CCCTATAGTGAGTCGTATTAATTTC | Supp. Figure 4 |
| 1287 | 1104-Mis-2-14 | CATTGGCATGGAAGTCACACCTTCGGCTGT TGCAGTTCCCCTGTCTACGGGGATTGTTAC CCTATAGTGAGTCGTATTAATTTC | Supp. Figure 4 |
| 1288 | 1104-Seed-1 | GTAAGGCATGGAAGTCACACCTTCGGGAG TTGCAGTTCCCCTGTCTACGGGGATTGTTA CCCTATAGTGAGTCGTATTAATTTC | Supp. Figure 4 |
| 1289 | 1104-Seed-2 | CATTCCGTTGGAAGTCACACCTTCGGGAGT TGCAGTTCCCCTGTCTACGGGGATTGTTAC CCTATAGTGAGTCGTATTAATTTC | Supp. Figure 4 |
| 1290 | 1104-Seed-3 | CATTGGCAACCTAGTCACACCTTCGGGAG TTGCAGTTCCCCTGTCTACGGGGATTGTTA CCCTATAGTGAGTCGTATTAATTTC | Supp. Figure 4 |
| 1291 | 1104-Seed-4 | CATTGGCATGGATCAGACACCTTCGGGAG TTGCAGTTCCCCTGTCTACGGGGATTGTTA CCCTATAGTGAGTCGTATTAATTTC | Supp. Figure 4 |
| 1292 | 1104-Seed-5 | CATTGGCATGGAAGTCTGTGCTTCGGGAG TTGCAGTTCCCCTGTCTACGGGGATTGTTA CCCTATAGTGAGTCGTATTAATTTC | Supp. Figure 4 |
| 1293 | 1104-Seed-6 | CATTGGCATGGAAGTCACACGAAGGGGAG TTGCAGTTCCCCTGTCTACGGGGATTGTTA CCCTATAGTGAGTCGTATTAATTTC | Supp. Figure 4 |
| 1294 | 1104-Seed-7 | CATTGGCATGGAAGTCACACCTTCCCCTGT TGCAGTTCCCCTGTCTACGGGGATTGTTAC CCTATAGTGAGTCGTATTAATTTC | Supp. Figure 4 |
| 1295 | 1104-Spacer-16 | CATGGAAGTCACACCTGTTGCAGTTCCCCT GTCTACGGGGATTGTTACCCTATAGTGAGT CGTATTAATTTC | Supp. Figure 4 |

| | | | |
|---|---|---|---|
| 1296 | 1104-Spacer-18 | GCATGGAAGTCACACCTTGTTGCAGTTCCCCTGTCTACGGGGATTGTTACCCTATAGTGAGTCGTATTAATTTC | Supp. Figure 4 |
| 1297 | 1104-Spacer-20 | GGCATGGAAGTCACACCTTCGTTGCAGTTCCCCTGTCTACGGGGATTGTTACCCTATAGTGAGTCGTATTAATTTC | Supp. Figure 4 |
| 1298 | 1104-Spacer-22 | TGGCATGGAAGTCACACCTTCGGTTGCAGTTCCCCTGTCTACGGGGATTGTTACCCTATAGTGAGTCGTATTAATTTC | Supp. Figure 4 |
| 1299 | 1104-Spacer-24 | TTGGCATGGAAGTCACACCTTCGGGTTGCAGTTCCCCTGTCTACGGGGATTGTTACCCTATAGTGAGTCGTATTAATTTC | Supp. Figure 4 |
| 1300 | 1104-Spacer-26 | ATTGGCATGGAAGTCACACCTTCGGGGTTGCAGTTCCCCTGTCTACGGGGATTGTTACCCTATAGTGAGTCGTATTAATTTC | Supp. Figure 4 |
| 1301 | 1104-Spacer-30 | gcattggcatggaagtcacaccttcgggaaGTTGCAGTTCCCCTGTCTACGGGGATTGTTACCCTATAGTGAGTCGTATTAATTTC | Supp. Figure 4 |
| 1303 | 1172-Mis 1-1 | ATGGCATGGAAGTCACACCTTCGGGTTGCAGTCTCCGCCTACATGGGAGTTGTGACCCTATAGTGAGTCGTATTAATTTC | Figure 3A |
| 1304 | 1172-Mis 1-2 | TAGGCATGGAAGTCACACCTTCGGGTTGCAGTCTCCGCCTACATGGGAGTTGTGACCCTATAGTGAGTCGTATTAATTTC | Figure 3A |
| 1305 | 1172-Mis 1-3 | TTCGCATGGAAGTCACACCTTCGGGTTGCAGTCTCCGCCTACATGGGAGTTGTGACCCTATAGTGAGTCGTATTAATTTC | Figure 3A |
| 1306 | 1172-Mis 1-4 | TTGCCATGGAAGTCACACCTTCGGGTTGCAGTCTCCGCCTACATGGGAGTTGTGACCCTATAGTGAGTCGTATTAATTTC | Figure 3A |
| 1307 | 1172-Mis 1-5 | TTGGGATGGAAGTCACACCTTCGGGTTGCAGTCTCCGCCTACATGGGAGTTGTGACCCTATAGTGAGTCGTATTAATTTC | Figure 3A |
| 1308 | 1172-Mis 1-6 | TTGGCTTGGAAGTCACACCTTCGGGTTGCAGTCTCCGCCTACATGGGAGTTGTGACCCTATAGTGAGTCGTATTAATTTC | Figure 3A |
| 1309 | 1172-Mis 1-7 | TTGGCAAGGAAGTCACACCTTCGGGTTGCAGTCTCCGCCTACATGGGAGTTGTGACCCTATAGTGAGTCGTATTAATTTC | Figure 3A |
| 1310 | 1172-Mis 1-8 | TTGGCATCGAAGTCACACCTTCGGGTTGCAGTCTCCGCCTACATGGGAGTTGTGACCCTATAGTGAGTCGTATTAATTTC | Figure 3A |

| | | | |
|---|---|---|---|
| 1311 | 1172-Mis 1-9 | TTGGCATGCAAGTCACACCTTCGGGTTGCAGTCTCCGCCTACATGGGAGTTGTGACCCTATAGTGAGTCGTATTAATTTC | Figure 3A |
| 1312 | 1172-Mis 1-10 | TTGGCATGGTAGTCACACCTTCGGGTTGCAGTCTCCGCCTACATGGGAGTTGTGACCCTATAGTGAGTCGTATTAATTTC | Figure 3A |
| 1313 | 1172-Mis 1-11 | TTGGCATGGATGTCACACCTTCGGGTTGCAGTCTCCGCCTACATGGGAGTTGTGACCCTATAGTGAGTCGTATTAATTTC | Figure 3A |
| 1314 | 1172-Mis 1-12 | TTGGCATGGAACTCACACCTTCGGGTTGCAGTCTCCGCCTACATGGGAGTTGTGACCCTATAGTGAGTCGTATTAATTTC | Figure 3A |
| 1315 | 1172-Mis 1-13 | TTGGCATGGAAGACACACCTTCGGGTTGCAGTCTCCGCCTACATGGGAGTTGTGACCCTATAGTGAGTCGTATTAATTTC | Figure 3A |
| 1316 | 1172-Mis 1-14 | TTGGCATGGAAGTGACACCTTCGGGTTGCAGTCTCCGCCTACATGGGAGTTGTGACCCTATAGTGAGTCGTATTAATTTC | Figure 3A |
| 1317 | 1172-Mis 1-15 | TTGGCATGGAAGTCTCACCTTCGGGTTGCAGTCTCCGCCTACATGGGAGTTGTGACCCTATAGTGAGTCGTATTAATTTC | Figure 3A |
| 1318 | 1172-Mis 1-16 | TTGGCATGGAAGTCAGACCTTCGGGTTGCAGTCTCCGCCTACATGGGAGTTGTGACCCTATAGTGAGTCGTATTAATTTC | Figure 3A |
| 1319 | 1172-Mis 1-17 | TTGGCATGGAAGTCACTCCTTCGGGTTGCAGTCTCCGCCTACATGGGAGTTGTGACCCTATAGTGAGTCGTATTAATTTC | Figure 3A |
| 1320 | 1172-Mis 1-18 | TTGGCATGGAAGTCACAGCTTCGGGTTGCAGTCTCCGCCTACATGGGAGTTGTGACCCTATAGTGAGTCGTATTAATTTC | Figure 3A |
| 1321 | 1172-Mis 1-19 | TTGGCATGGAAGTCACACGTTCGGGTTGCAGTCTCCGCCTACATGGGAGTTGTGACCCTATAGTGAGTCGTATTAATTTC | Figure 3A |
| 1322 | 1172-Mis 1-20 | TTGGCATGGAAGTCACACCATCGGGTTGCAGTCTCCGCCTACATGGGAGTTGTGACCCTATAGTGAGTCGTATTAATTTC | Figure 3A |
| 1323 | 1172-Mis 1-21 | TTGGCATGGAAGTCACACCTACGGGTTGCAGTCTCCGCCTACATGGGAGTTGTGACCCTATAGTGAGTCGTATTAATTTC | Figure 3A |
| 1324 | 1172-Mis 1-22 | TTGGCATGGAAGTCACACCTTGGGGTTGCAGTCTCCGCCTACATGGGAGTTGTGACCCTATAGTGAGTCGTATTAATTTC | Figure 3A |

| | | | |
|---|---|---|---|
| 1325 | 1172-Mis 1-23 | TTGGCATGGAAGTCACACCTTCCGGTTGCAGTCTCCGCCTACATGGGAGTTGTGACCCTATAGTGAGTCGTATTAATTTC | Figure 3A |
| 1326 | 1172-Mis 1-24 | TTGGCATGGAAGTCACACCTTCGCGTTGCAGTCTCCGCCTACATGGGAGTTGTGACCCTATAGTGAGTCGTATTAATTTC | Figure 3A |
| 1327 | 1172-Mis-2-1 | AAGGCATGGAAGTCACACCTTCGGGTTGCAGTCTCCGCCTACATGGGAGTTGTGACCCTATAGTGAGTCGTATTAATTTC | Figure 3B |
| 1328 | 1172-Mis-2-2 | TTCCCATGGAAGTCACACCTTCGGGTTGCAGTCTCCGCCTACATGGGAGTTGTGACCCTATAGTGAGTCGTATTAATTTC | Figure 3B |
| 1329 | 1172-Mis-2-3 | TTGGGTTGGAAGTCACACCTTCGGGTTGCAGTCTCCGCCTACATGGGAGTTGTGACCCTATAGTGAGTCGTATTAATTTC | Figure 3B |
| 1330 | 1172-Mis-2-4 | TTGGCAACGAAGTCACACCTTCGGGTTGCAGTCTCCGCCTACATGGGAGTTGTGACCCTATAGTGAGTCGTATTAATTTC | Figure 3B |
| 1331 | 1172-Mis-2-5 | TTGGCATGCTAGTCACACCTTCGGGTTGCAGTCTCCGCCTACATGGGAGTTGTGACCCTATAGTGAGTCGTATTAATTTC | Figure 3B |
| 1332 | 1172-Mis-2-6 | TTGGCATGGATCTCACACCTTCGGGTTGCAGTCTCCGCCTACATGGGAGTTGTGACCCTATAGTGAGTCGTATTAATTTC | Figure 3B |
| 1333 | 1172-Mis-2-7 | TTGGCATGGAAGAGACACCTTCGGGTTGCAGTCTCCGCCTACATGGGAGTTGTGACCCTATAGTGAGTCGTATTAATTTC | Figure 3B |
| 1334 | 1172-Mis-2-8 | TTGGCATGGAAGTCTGACCTTCGGGTTGCAGTCTCCGCCTACATGGGAGTTGTGACCCTATAGTGAGTCGTATTAATTTC | Figure 3B |
| 1335 | 1172-Mis-2-9 | TTGGCATGGAAGTCACTGCTTCGGGTTGCAGTCTCCGCCTACATGGGAGTTGTGACCCTATAGTGAGTCGTATTAATTTC | Figure 3B |
| 1336 | 1172-Mis-2-10 | TTGGCATGGAAGTCACACGATCGGGTTGCAGTCTCCGCCTACATGGGAGTTGTGACCCTATAGTGAGTCGTATTAATTTC | Figure 3B |
| 1337 | 1172-Mis-2-11 | TTGGCATGGAAGTCACACCTAGGGGTTGCAGTCTCCGCCTACATGGGAGTTGTGACCCTATAGTGAGTCGTATTAATTTC | Figure 3B |
| 1338 | 1172-Mis-2-12 | TTGGCATGGAAGTCACACCTTCCCGTTGCAGTCTCCGCCTACATGGGAGTTGTGACCCTATAGTGAGTCGTATTAATTTC | Figure 3B |

| | | | |
|---|---|---|---|
| 1339 | 1172-Seed-1 | AACCCATGGAAGTCACACCTTCGGGTTGCAGTCTCCGCCTACATGGGAGTTGTGACCCTATAGTGAGTCGTATTAATTTC | Figure 3C |
| 1340 | 1172-Seed-2 | TTGGGTACGAAGTCACACCTTCGGGTTGCAGTCTCCGCCTACATGGGAGTTGTGACCCTATAGTGAGTCGTATTAATTTC | Figure 3C |
| 1341 | 1172-Seed-3 | TTGGCATGCTTCTCACACCTTCGGGTTGCAGTCTCCGCCTACATGGGAGTTGTGACCCTATAGTGAGTCGTATTAATTTC | Figure 3C |
| 1342 | 1172-Seed-4 | TTGGCATGGAAGAGTGACCTTCGGGTTGCAGTCTCCGCCTACATGGGAGTTGTGACCCTATAGTGAGTCGTATTAATTTC | Figure 3C |
| 1343 | 1172-Seed-5 | TTGGCATGGAAGTCACTGGATCGGGTTGCAGTCTCCGCCTACATGGGAGTTGTGACCCTATAGTGAGTCGTATTAATTTC | Figure 3C |
| 1344 | 1172-Seed-6 | TTGGCATGGAAGTCACACCTAGCCGTTGCAGTCTCCGCCTACATGGGAGTTGTGACCCTATAGTGAGTCGTATTAATTTC | Figure 3C |
| 1345 | 1172-Spacer-16 | CATGGAAGTCACACCTGTTGCAGTCTCCGCCTACATGGGAGTTGTGACCCTATAGTGAGTCGTATTAATTTC | Figure 3D |
| 1346 | 1172-Spacer-18 | GCATGGAAGTCACACCTTGTTGCAGTCTCCGCCTACATGGGAGTTGTGACCCTATAGTGAGTCGTATTAATTTC | Figure 3D |
| 1347 | 1172-Spacer-20 | GGCATGGAAGTCACACCTTCGTTGCAGTCTCCGCCTACATGGGAGTTGTGACCCTATAGTGAGTCGTATTAATTTC | Figure 3D |
| 1348 | 1172-Spacer-22 | TGGCATGGAAGTCACACCTTCGGTTGCAGTCTCCGCCTACATGGGAGTTGTGACCCTATAGTGAGTCGTATTAATTTC | Figure 3D |
| 1349 | 1172-Spacer-26 | ATTGGCATGGAAGTCACACCTTCGGGGTTGCAGTCTCCGCCTACATGGGAGTTGTGACCCTATAGTGAGTCGTATTAATTTC | Figure 3D |
| 1350 | 1172-Spacer-28 | CATTGGCATGGAAGTCACACCTTCGGGAGTTGCAGTCTCCGCCTACATGGGAGTTGTGACCCTATAGTGAGTCGTATTAATTTC | Figure 3D |
| 1351 | 1172-Spacer-30 | GCATTGGCATGGAAGTCACACCTTCGGGAAGTTGCAGTCTCCGCCTACATGGGAGTTGTGACCCTATAGTGAGTCGTATTAATTTC | Figure 3D |
| 1215 | *ORF1* LwaCas13a crRNA | GTTAAACCAGGTGGAACCTCATCAGGAGATGTTTTAGTCCCCTTCGTTTTGGGGTAGTCTAAATCCCCTATAGTGAGTCGTATTAATTTC | Figure 2C |

| | | | |
|---|---|---|---|
| | | | |
| 1363 | Tcc-crRNA-HCV-1 | TAGTGGTCTGCGGAACCGGTGAGTGTTGC AGTCTCCGCCTACATGGGAGTTGTGACCCT ATAGTGAGTCGTATTAATTTC | Supp. Figure 21 |
| 1370 | Tcc-crRNA-TYLCV-2 | AAGGCCTTATGGAAGCAGCCCAATGTTGC AGTCTCCGCCTACATGGGAGTTGTGACCCT ATAGTGAGTCGTATTAATTTC | Supp. Figure 21 |
| CV627 | AapCas12b-RnaseP-sgRNA-1 | AGGGTCACACCCAAGTAATTGTGCCACTT CTCAGATTTGAG | Supp. Figure 15 |
| CV628 | AapCas12b-RnaseP-sgRNA-2 | CTCGGACCAGAGCCATGTAAGTGCCACTT CTCAGATTTGAG | Supp. Figure 15 |
| CV629 | AapCas12b-RnaseP-sgRNA-3 | AATTACTTGGGTGTGACCCTGTGCCACTTC TCAGATTTGAG | Supp. Figure 15 |
| CV241 | AapCas12b sgRNA scaffold-Top | GAAATTAATACGACTCACTATAGGGTCTA GAGGACAGAATTTTTCAACGGGTGTGCCA ATGGCCACTTTCCAGGTGGCAAAGCCCGT TGAGCTTCTCAAATCTGAGAAGTGGCAC | |

- crRNA sequences shown as 5`→3` reverse complement to be annealed with **T7 oligo** for *in vitro* transcription.

**Table S3: Pre-crRNAs of LwaCas13a, HheCas13a, and TccCas13a**

| crRNA # | Name | Sequence (5`→`3) | Note |
|---|---|---|---|
| 1377 | LwaCas13a -pre-crRNA-5` FAM labeled | FAM-AUCAUGCGAGGAUUUAGACUACCCCAAAAACGAAGG GGACUAAAACUUCAGAUAUAGCCUGGUGGUUCAGGC | - Figure 3E<br>- Supp. Figure 5 |
| 1378 | HheCas13a-pre-crRNA-5` FAM labeled | FAM-ACUGAGGUAUGUAACAAUCCCCGUAGACAGGGGAAC UGCAACCAGAUAUAGCCUGGUGGUUCAGGC | - Figure 3E<br>- Supp. Figure 5 |

| | | | |
|---|---|---|---|
| 1379 | TccCas13a-pre-crRNA-5` FAM labeled | GUCGACACGGGUCACAACUCCCAUGUAGGCGGAGAC UGCAACUAUUACCACUCAGGGUAAUGUCCG | - Figure 3E<br>- Supp. Figure 5 |

**Table S4: Primers to PCR amplify DNA templates for in *vitro* transcription to generate RNA targets.**

| **Name** | **Sequence (5`→`3)** | **Note** |
|---|---|---|
| N gene-1-T7-F | GAAATTAATACGACTCACTATAGGGAG GCTTCTAAGAAGCCTCGGC | To amplify targeted region in *N* gene to produce the RNA target used in figure 2 targeted with HheCas13a and TccCas13a |
| N gene-1-R_2 | CTGTCTCTGCGGTAAGGCTTGAG | |
| T7 oligo | GAAATTAATACGACTCACTATAGGG | To amplify *ORF1* gBlock to produce the RNA target used in figure 2 targeted with LwaCas13a |
| ORF1-R | GACATACTTATCGGCAATTTTGTTACC | |
| T7 oligo | GAAATTAATACGACTCACTATAGGG | To amplify HCV genotypes 1a and 2b gBlocks to produce the RNA target used in Fig. S15 |
| M13-R | CAGGAAACAGCTATGAC | |

**Table S5: gBlocks used in this study.**

| **Name** | **Sequence (5`→3)** |
|---|---|
| SARS-CoV-2 *ORF1* gBlock | GAAATTAATACGACTCACTATAGGGCCTCACTTGTTCTTGCTCGCAAACA TACAACGTGTTGTAGCTTGTCACACCGTTTCTATAGATTAGCTAATGAGT GTGCTCAAGTATTGAGTGAAATGGTCATGTGTGGCGGTTCACTATATGTT AAACCAGGTGGAACCTCATCAGGAGATGCCACAACTGCTTATGCTAATA GTGTTTTTAACATTTGTCAAGCTGTCACGGCCAATGTTAATGCACTTTTA TCTACTGATGGTAACAAAATTGCCGATAAGTATGTC |
| HCV genotype 1a | GAAATTAATACGACTCACTATAGGGTTGGGGGCGACACTCCACCATGAA TCACTCCCCTGTGAGGAACTACTGTCTTCACGCAGAAAGCGTCTAGCCAT GGCGTTAGTATGAGTGTCGTGCAGCCTCCAGGACCCCCCCTCCCGGGAG AGCCATAGTGGTCTGCGGAACCGGTGAGTACACCGGAATTGCCAGGACG ACCGGGTCCTTTCTTGGATAAACCCGCTCAATGCCTGGAGATTTGGGCGT GCCCCCGCAAGACTGCTAGCCGAGTAGTGTTGGGTCGCGAAAGGCCTTG |

| | |
|---|---|
| | TGGTACTGCCTGATAGGGTGCTTGCGAGTGCCCCGGGAGGTCTCGTAGA CCGTGCACCATGAGCACGAATCCTAAACCCCAAAGAAAAACCAAACGT AACACCAACCGTCGCCCACAGGACGTCAAGTTCCCGGGTGGCGGTCAGA TCGTTGGTGGAGTTTACTTGTTGCCGCGCAGGGGCCGTCATAGCTGTTTC CTG |
| HCV genotype 2b | GAAATTAATACGACTCACTATAGGGTTGGGGGCGACACTCCGCCATGAA TCACTCCCCTGTGAGGAACTACTGTCTTCACGCAGAAAGCGTCTAGCCAT GGCGTTAGTATGAGTGTCGTACAGCCTCCAGGCCCCCCCTCCCGGGAG AGCCATAGTGGTCTGCGGAACCGGTGAGTACACCGGAATTACCGGAAAG ACTGGGTCCTTTCTTGGATAAACCCACTCTATGTCCGGTCATTTGGGCAC GCCCCCGCAAGACTGCTAGCCGAGTAGCGTTGGGTTGCGAAAGGCCTTG TGGTACTGCCTGATAGGGTGCTTGCGAGTGCCCCGGGAGGTCTCGTAGA CCGTGCATCATGAGCACAAATCCTAAACCTCAAAGAAAAACCAAAAGA AACACAAACCGCCGCCCACAGGACGTTAAGTTCCCGGGTGGCGGTCAGA TCGTTGGCGGAGTTTACTTGCTGCCGCGCAGGGGCCGTCATAGCTGTTTC CTG |

**Table S6: RNA reporter designs and sequences used in this study.**

| | |
|---|---|
| Poly U reporter-FAM | 5'-/56-FAM/rUrUrUrUrUrU/3IABKFQ/-3' |
| Poly A reporter-FAM | 5'-/56-FAM/rArArArArArA/3IABKFQ/-3' |
| Poly G reporter-FAM | 5'-/56-FAM/rGrGrGrGrGrG/3IABkFQ/-3' |
| Poly C reporter-FAM | 5'-/56-FAM/rCrCrCrCrCrC/3IABkFQ/-3' |
| 3(AG) reporter-FAM | 5'-/56-FAM/rArGrArGrArG/3IABkFQ/-3' |
| 3(AC) reporter-FAM | 5'-/56-FAM/rArCrArCrArC/3IABkFQ/-3' |
| 3(CG) reporter-FAM | 5'-/56-FAM/rCrGrCrGrCrG/3IABkFQ/-3' |
| 3(UG) reporter-FAM | 5'-/56-FAM/rUrGrUrGrUrG/3IABkFQ/-3' |
| Mix reporter-FAM | 5'-/56-FAM/rUrGrArCrGrU/3IABKFQ/-3' |
| Mix reporter-HEX | 5'-/HEX/rUrGrArCrGrU/3IABKFQ/-3' |
| LwaCas13a reporter | 5'-/56-FAM/rUrUrArUrU/3IABKFQ/-3' |
| AapCas12b reporter-HEX | 5'-/HEX/TTTTTTT/3IABKFQ/-3' |

**Table S7: Protein sequences.**

| Name | Protein sequence |
|---|---|
| TccCas13a | MGSSHHHHHHSSGLVPRGSHMASWSHPQFEKGGGSGGGSGGSAWSHPQFEK MSDSEVNQEAKPEVKPEVKPETHINLKVSDGSSEIFFKIKKTTPLRRLMEAFAK RQGKEMDSLRFLYDGIRIQADQTPEDLDMEDNDIIEAHREQIGGSMKITKRKW GEHHPPLYFYRDEDSGRLLAQNDRKQDYTDTLFNDIAQDTFERSLRNRLLKTP EKGDKRFYSNEIVKLVEKLCQGADVAEIMKSMERNEKLRPKNEKEIKNLKKQL DGTLSEYGKRYTAPEGAMTLNDALFYLVEGNPLKQAMAKAELGKIREALIKE KENRINRVRYSIKNNKIPLRIQEDGGITPNNDRAAWLLGLMKPADPAKGITDCY PLLGELEEVFDFDKLSKTLHEKISRCQGRPRSIAMAVDEALKQYLRELWEKSPS RQQDLKYYFQAVQEYFKDNFPIRTKRMGARLRQELLKDKTSLSRLLEPKHMA NAVRRRLINQSTQMHILYGKLYAYCCGEDGRLLVNSETLQRIQVHEAVKKQA MTAVLWSISRLRYFYQFEDGDILSNKNPIKDFRDKFLRDTNKYTHEDVEACKE KLQDFFPLKELQEKIKEDAKGLQETDNKQADTTDFKAIGHIVRDDRKLCNQLL AECVSCIGELRHHIFHYKNVTLIQALKRIADKVKPEDLSVLRAIYLLDRRNLKK AFAKRISSMNLPLYYREDLLSRIFKKEGTAFFLYSAKIQMTPSFQRVYERGKNL RREFECERMKAEASNGQNGQDGDRLKWFRQLAAGDSADTHFNWAVEAYAES AADVENNVEFDTDVDAQRALRNLLLLIYRHHFLPEVQKDETLVTGKIHKVLER NRQLSEGQGPNQGKAHGYSVIEELYHEGMPLSDLMKQLQRRISETERESRELA QEKTDYAQRFILDIFAEAFNDFLEAHYGEEYLEIMSPRKDAEAAKKWVKESKT VDLKTSIDEKEPEGHLLVLYPVLRLLDERELGELQQQMIRYRTSLASWQGESNF SEEIRIAGQIEELTELVKLTEPEPQFAEEVWGKRAKEAFEDFIEGNMKNYEAFY LQSDNNTPVYRRNMSRLLRSGLMGVYQKVLASHKQALKRDYLLWSEKHWN VKDENGADISSAEQAQCLLQRLHRKYAESPSRFTEEDCKLYEKVLRRLEDYNQ AVKNLSFSSLYEICVLNLEILSRWVGFVQDWERDMYFLLLAWVRQGKLDGIKE EDVRDIFSEGNIIRNLVDTLKGENMNAFESVYFPENKGSKYLGVRNDVAHLDL MRKNGWRLEAGKTCSVMEDYINRLRFLLSYDQKRMNAVTKTLQQIFDRHKV KIRFTVEKGGMLKIEDVTADKIVHLKGSRLSGIEIPSHGERFIDTLKALMVYPRG * |

6x His affinity tag, Thrombin site, Strep-tag II, SUMO, TccCas13a protein.

**Table S8: Back-of-the-Envelope Checks for TccCas13a Michaelis-Menten enzyme kinetics.**

| $E_0$ (nM) | $t_{lin}$ (s) | $S_0$ (nM) | v (nM/s) | α | β | γ |
|---|---|---|---|---|---|---|
| 0.5 | 600 | 4000 | 0.296 | 0.044 | 0.371 | 0.072 |
| | | 2000 | 0.195 | 0.059 | 0.245 | |
| | | 1000 | 0.103 | 0.062 | 0.129 | |
| | | 500 | 0.039 | 0.047 | 0.049 | |
| | | 250 | 0.031 | 0.073 | 0.038 | |
| | | 125 | 0.015 | 0.074 | 0.019 | |
| | | 62.5 | 0.008 | 0.073 | 0.010 | |
| | | 31.25 | 0.003 | 0.060 | 0.004 | |

**Table S9: Clinical samples used in this study.**

| # | Sample ID in figures | Sample ID | Ct value (SARS-CoV-2 N gene) | SARS-CoV-2 strain |
|---|---|---|---|---|
| **Used in Fig. 5c&d** | | | | |
| 95 | | A1 | 20.20318222 | Delta |
| 96 | | A2 | 23.8019371 | Delta |
| 97 | | A3 | 20.38153458 | Delta |
| 98 | | A4 | 23.91605568 | Beta |
| 99 | | A5 | 21.15932465 | Delta |
| 100 | | A6 | 20.17287445 | Beta |
| 101 | | A7 | 20.63743782 | Delta |
| 102 | | A8 | 26.51057625 | Delta |
| 103 | | A9 | 24.15272903 | Delta |
| 104 | | A10 | 26.29830933 | Delta |
| 105 | | A11 | 22.10408401 | Delta |

| | | | | |
|---|---|---|---|---|
| 106 | | A12 | 26.71198273 | Delta |
| 107 | | B1 | 32.79143906 | Delta |
| 108 | | B2 | 30.94537354 | Delta |
| 109 | | B3 | 19.86674881 | Delta |
| 110 | | B4 | 26.80452538 | Delta |
| 111 | | B5 | 23.44918823 | Delta |
| 112 | | B6 | 23.47177696 | Delta |
| 113 | | B7 | 25.94287682 | Delta |
| 114 | | B8 | 26.83697891 | Beta |
| 115 | | B9 | 26.61860275 | Delta |
| 116 | | B10 | 29.60500526 | Beta |
| 117 | | B11 | 28.60080528 | Delta |
| 118 | | B12 | 21.05815697 | Delta |
| 119 | | C1 | 23.73565865 | Delta |
| 120 | | C2 | 20.14953613 | Delta |
| 121 | | C3 | 24.65905762 | Delta |
| 122 | | C4 | 20.69680595 | Delta |
| 123 | | C5 | 24.33411217 | Alpha |
| 124 | | C6 | 30.21217537 | Delta |
| 125 | | C7 | 30.52635384 | Beta |
| 126 | | C8 | 25.62018967 | Delta |
| 127 | | C9 | 26.6070385 | Delta |
| 128 | | C10 | 16.92820358 | Alpha |
| 129 | | C11 | 26.35331726 | Alpha |
| 130 | | C12 | 16.99542999 | Beta |
| 131 | | D1 | 15.77931976 | Other |
| 132 | | D2 | 26.65350533 | Delta |
| 133 | | D3 | 18.5001297 | Alpha |
| 134 | | D4 | 31.98776627 | Delta |
| 135 | | D5 | 16.7693367 | Delta |

| | | | | |
|---|---|---|---|---|
| 136 | | D6 | 34.24099731 | Delta |
| 137 | | D7 | 21.01351547 | Other |
| 138 | | D8 | 16.75134087 | Delta |
| 139 | | D9 | 29.01878738 | Delta |
| 140 | | D10 | 27.75723839 | Delta |
| 141 | | D11 | 29.67990685 | Other |
| 142 | | D12 | 20.9514904 | Other |
| 143 | | E1 | 24.57509232 | Delta |
| 144 | | E2 | 20.96550179 | Delta |
| 145 | | E3 | 26.76332283 | Beta |
| 146 | | E4 | 25.0199337 | Other |
| 147 | | E5 | 29.9657135 | Beta |
| 149 | | E7 | 27.49075508 | Alpha |
| 150 | | E8 | 22.13245583 | Delta |
| 151 | | E9 | 23.42307281 | Delta |
| 152 | | E10 | 24.99167442 | Delta |
| 153 | | E11 | 23.46128845 | Delta |
| 154 | | E12 | 19.64938736 | Delta |
| 155 | | F1 | 28.25497818 | Delta |
| 156 | | F2 | 20.91403008 | Beta |
| 157 | | F3 | 23.19413948 | Delta |
| 158 | | F4 | 33.09214783 | Alpha |
| 159 | | F5 | 34.40586853 | Delta |
| 160 | | F6 | 30.73725128 | Other |
| 161 | | F7 | 23.17684937 | Other |
| 162 | | F8 | 21.75904465 | Alpha |
| 163 | | F9 | 34.17617035 | Beta |
| 164 | | F10 | 27.16244316 | Alpha |
| 165 | | F11 | 19.42002296 | Beta |
| 166 | | F12 | 33.7602272 | Alpha |

| | | | | |
|---|---|---|---|---|
| 167 | | G1 | 35.41335297 | Alpha |
| 168 | | G2 | 17.82286072 | Other |
| 169 | | G3 | Undetermined | |
| 170 | | G4 | Undetermined | |
| 171 | | G5 | Undetermined | |
| 172 | | G6 | Undetermined | |
| 173 | | G7 | Undetermined | |
| 174 | | G8 | Undetermined | |
| 175 | | G9 | Undetermined | |
| 176 | | G10 | Undetermined | |
| 177 | | G11 | Undetermined | |
| 178 | | G12 | Undetermined | |
| 179 | | H1 | Undetermined | |
| 180 | | H2 | Undetermined | |
| 181 | | H3 | Undetermined | |
| 182 | | H4 | Undetermined | |
| 183 | | H5 | Undetermined | |
| 184 | | H6 | Undetermined | |
| 185 | | H7 | Undetermined | |
| 186 | | H8 | Undetermined | |
| 187 | | H9 | Undetermined | |
| 188 | | H10 | Undetermined | |
| 189 | | H1 | Undetermined | |
| 190 | | H2 | Undetermined | |
| 192 | | H4 | Undetermined | |
| 193 | | H5 | Undetermined | |
| 194 | | H6 | Undetermined | |
| 195 | | H7 | Undetermined | |
| 197 | | H9 | Undetermined | |
| **Used in Fig. S13** | | | | |

| | | | | |
|---|---|---|---|---|
| 396 | **A12** | 160943 | 14 | |
| 397 | **B1** | 160944 | 20 | |
| 398 | **B2** | 160947 | 14 | |
| 410 | **C2** | 160992 | 23 | |
| 421 | **D1** | 164191 | 17 | |
| 444 | **E12** | 164515 | 14 | |
| 445 | **F1** | 164520 | 27 | |
| 446 | **F2** | 164525 | 27 | |
| 447 | **F3** | 164527 | 19 | |
| 448 | **F4** | 164530 | 19 | |
| 449 | **F5** | 164532 | 27 | |
| 450 | **F6** | 164537 | 27 | |
| 451 | **F7** | 164539 | 18 | |
| 452 | **F8** | 164553 | 20 | |
| 453 | **F9** | 164580 | 34 | |
| 454 | **F10** | 164581 | 20 | |
| 455 | **F11** | 164583 | 18 | |
| 456 | **F12** | 164589 | 22 | |
| 457 | **G1** | 164596 | 31 | |
| 458 | **G2** | 164597 | 15 | |
| 459 | **G3** | 164598 | 24 | |
| 460 | **G4** | 164603 | 18 | |
| 461 | **G5** | 164604 | 18 | |
| 462 | **G6** | 164605 | 19 | |
| 463 | **G7** | 164624 | 25 | |
| 465 | **G9** | 164649 | 21 | |
| 466 | **G10** | 164650 | 22 | |
| 467 | **G11** | 164651 | 17 | |
| 468 | **G12** | 164654 | 26 | |
| 469 | **H1** | 164661 | 22 | |

| | | | | |
|---|---|---|---|---|
| 470 | **H2** | 164662 | 18 | |
| 472 | **H4** | 164665 | 25 | |
| 473 | **H5** | 164667 | 20 | |
| 474 | **H6** | 166758 | 24 | |
| 475 | **H7** | 166759 | 19 | |
| 476 | **H8** | 166760 | 21 | |
| 477 | **H9** | 166761 | 14 | |
| 478 | **H10** | 166762 | 23 | |
| 479 | **H11** | 166763 | 16 | |
| 480 | **H12** | 166764 | 24 | |
| K0002 | **–ve 1** | K0002 | Undetermined | |
| K0005 | **–ve 2** | K0005 | Undetermined | |
| K0006 | **–ve 3** | K0006 | Undetermined | |
| K0007 | **–ve 4** | K0007 | Undetermined | |
| K0008 | **–ve 5** | K0008 | Undetermined | |
| **Used in Fig. 6c** | | | | |
| K0244 | **14** | | 14 | |
| K0246 | **15.6** | | 15.6 | |
| K0250 | **16.8** | | 16.8 | |
| K0247 | **17.5** | | 17.5 | |
| K0249 | **17.8** | | 17.8 | |
| K0254 | **15.5** | | 15.5 | |
| K0252 | **17.5** | | 17.5 | |
| K0203 | **16.7** | | 16.7 | |
| K0209 | **19.2** | | 19.2 | |
| K0200 | **16.9** | | 16.9 | |
| K0253 | **30.7** | | 30.7 | |
| K0213 | **14.7** | | 14.7 | |
| K0068 | **17** | | 17 | |
| K0265 | **16.1** | | 16.1 | |

| | | | | |
|---|---|---|---|---|
| **Used in Fig. 6d** | | | | |
| 1 | **22.23623848** | | 22.23623848 | |
| 2 | **20.20703506** | | 20.20703506 | |
| 4 | **14.83642673** | | 14.83642673 | |
| 6 | **16.92188454** | | 16.92188454 | |
| 7 | **22.07608795** | | 22.07608795 | |
| 8 | **20.47973633** | | 20.47973633 | |
| 9 | **21.8478508** | | 21.8478508 | |
| 10 | **26.23923874** | | 26.23923874 | |
| 11 | **22.94090843** | | 22.94090843 | |
| 12 | **22.38891029** | | 22.38891029 | |
| 13 | **22.45388031** | | 22.45388031 | |
| 14 | **22.93203545** | | 22.93203545 | |
| 15 | **28.23180008** | | 28.23180008 | |
| 16 | **27.80712509** | | 27.80712509 | |
| 5 | **-Ve** | | Undetermined | |
| 18 | **-Ve** | | Undetermined | |
| **Used in Fig. S14** | | | | |
| 1 | **1** | | 18.57 | |
| 2 | **2** | | 24.62 | |
| 3 | **3** | | 19.65 | |
| 4 | **4** | | 30.72 | |
| 5 | **5** | | 32.33 | |
| 6 | **6** | | 21.14 | |
| 7 | **7** | | 31.9 | |
| 8 | **8** | | 34.9 | |
| 9 | **9** | | 19.93 | |
| 10 | **10** | | 16.59 | |
| 11 | **11** | | 18.46 | |
| 12 | **12** | | 17.81 | |

| | | | | |
|---|---|---|---|---|
| 13 | **13** | | 19.82 | |
| 14 | **14** | | 18.2 | |
| 15 | **15** | | 23.64 | |
| 16 | **16** | | 35.57 | |
| 17 | **17** | | 20.72 | |
| 18 | **18** | | 16.57 | |
| 19 | **19** | | 30 | |
| 20 | **20** | | 18.98 | |
| 21 | **21** | | 31.13 | |
| 22 | **22** | | 27.63 | |
| -Ve | **-Ve** | | Undetermined | |
| -Ve | **-Ve** | | Undetermined | |

**Supplementary Note 1: Assembly of OPTIMA-dx reaction.**

1- Dilute TccCas13a protein into 1 µM in 1x isothermal buffer (Lucigen, 30027).
2- Assemble TccCas13a RNP as follows:

| **Reagents** | **Concentration** | **Amount (µL)** | **Final concentration** |
|---|---|---|---|
| $H_20$ | | 12 | |
| Isothermal buffer | 10X | 3 | 1X |
| TccCas13a | 1 µM | 7.5 | 250 nM |
| TccCas13a crRNA#1172 | 1 µM | 7.5 | 250 nM |
| Total | | 30 (enough for 6 reactions) | |

3- Keep the assembly at room temperature while assembling the OPTIMA-dx reaction.

4- Assemble OPTIMA-dx master mix as follows:

| **Reagents** | **Concentration** | **Amount (µL)** | **Final concentration** |
|---|---|---|---|
| $H_2O$ | | 0.65 | |
| Isothermal buffer | 10X | 2.5 | 1X |
| $MgSO_4$ | 100 mM | 1.5 | 6 mM |
| dNTPs | 25 mM | 1.4 | 1.4 mM |
| SC LAMP primers | 10X | 2.5 | 1X |
| NTPs | 10 mM | 1.25 | 0.5 mM |
| *Bst* DNA polymerase | 50 U/µL | 1.2 | 2.4 U/µL |
| RTx reverse transcriptase | 15 U/µL | 0.5 | 0.3U/µL |
| RNasin Plus RNase inhibitor | 40 U/µL | 0.5 | 0.8 U/µL |
| Hi-T7 RNA polymerase | 50 U/µL | 2 | 4 U/µL |
| Inorganic pyrophosphatase | 20 U/µL | 0.5 | 0.4 U/µL |

| | | | |
|---|---|---|---|
| HEX mix reporter | 18.75 µM | 1 | 750 nM |
| RNP | 250 nM | 5 | 50 nM |
| template | | 4.5 | |
| Total | | 25 | |

5- Incubate the reaction at 56 °C for 60 mins.

6- Place the reaction tubes in p51 molecular fluorescence viewer and visualize the results.

**Supplementary Note 2: Assembly of multiplexed OPTIMA-dx reaction.**

1- Dilute TccCas13a protein into 2 µM in 1x isothermal buffer (Lucigen, 30027).
2- Dilute AapCas12b protein into 2 µM in 1x isothermal buffer (Lucigen, 30027).
3- Assemble of TccCas13a and AapCas12b RNPs as follows in 2 different tubes:
   - TccCas13a RNP assembly:

| **Reagents** | **Concentration** | **Amount (µL)** | **Final concentration** |
|---|---|---|---|
| $H_20$ | | 4.5 | |
| Isothermal buffer | 10X | 3 | 1X |
| TccCas13a | 2 µM | 7.5 | 500 nM |
| TccCas13a crRNA (#1172 for SARS-CoV2 or #1363 for HCV) | 1 µM | 15 | 500 nM |
| Total | | 30 (enough for 6 reactions) | |

- AapCas12b RNP assembly:

| Reagents | Concentration | Amount (µL) | Final concentration |
|---|---|---|---|
| $H_20$ | | 4.5 | |
| Isothermal buffer | 10X | 3 | 1X |
| AapCas12b | 2 µM | 7.5 | 500 nM |
| AapCas12b sgRNA-1 for RNase P detection. | 1 µM | 15 | 500 nM |
| Total | | 30 (enough for 6 reactions) | |

4- Keep the RNPs at room temperature while assembling the multiplexed OPTIMA-dx reaction.

5- Assemble multiplexed OPTIMA-dx master mix as follows:

| Reagents | Concentration | Amount (µL) | Final concentration |
|---|---|---|---|
| $H_2O$ | | 0 | |
| Isothermal buffer | 10X | 5 | 1X |
| $MgSO_4$ | 100 mM | 3 | 6 mM |
| dNTPs | 25 mM | 2.8 | 1.4 mM |
| SC LAMP primers (or HCV primers) | 10X | 5 | 1X |
| RNase P primers | 10X | 5 | 1X |
| NTPs | 10 mM | 2.5 | 0.5 mM |
| *Bst* DNA polymerase | 50 U/µL | 2.4 | 2.4 U/µL |
| RTx reverse transcriptase | 15 U/µL | 1 | 0.3U/µL |
| RNasin Plus RNase inhibitor | 40 U/µL | 1 | 0.8 U/µL |

| | | | |
|---|---|---|---|
| Hi-T7 RNA polymerase (High concentration (M0470T, NEB) | 1000 U/µL | 0.2 | 4 U/µL |
| Inorganic pyrophosphatase | 20 U/µL | 1 | 0.4 U/µL |
| FAM RNA mix reporter | 25 µM | 0.5 | 250 nM |
| HEX ssDNA reporter | 10 µM | 1.25 | 250 nM |
| TccCas13a RNP | 500 nM | 5 | 50 nM |
| AapCas12b RNP | 500 nM | 5 | 50 nM |
| template | | 9.35 | |
| Total | | 50.65 | |

6- Incubate the reaction at 56 °C for 60 mins in a 96-well Real-Time PCR detection system (CFX96 qPCR machine, Bio-Rad), with fluorescence measurements taken every 2 min using both FAM and HEX channels.

**Supplementary Note 3: OPTIMA-dx Software Implementation**

The dataset of fluorescent images used for training the software consisted of many random images annotated manually as positive or negative to set the proper fluorescence intensity threshold. The software was then trained and tested multiple times to reach the best mean average precision (mAP) value with this dataset. The application allows the user to easily take a picture of PCR strips or upload an already captured image of a PCR strip illuminated by a transilluminator. The software then determines the location of each tube, calculates a probability score for each target category and classifies each tube as positive (green bounding box) or negative (red bounding box) samples based on the intensity of the fluorescent signal (Fig. 6d). The entire image processing, from capturing the reaction tubes to the final app output results, takes less than 1 min. Once the image is captured, it can be uploaded and processed by the software.

- **Deep Learning Framework**

As of today, there are various deep learning frameworks for engineers and researchers to choose to train machine learning models [8-11]. These frameworks abstract the underlying hardware and software stack to expose a simple API in language such as Python. Among these frameworks, TensorFlow is one of the most popular frameworks in deep learning community [8]. Compared with TensorFlow, TensorFlow-Lite (TensorFlow Lite. https://www.tensorflow.org/lite.) is the lightweight version of TensorFlow, which is specifically designed for the mobile platform and embedded devices. In this project, we use TensorFlow framework to train our model on Linux workstation and use TensorFlow-Lite to deploy the trained model on mobile device with Android operating system.

- **Object Detection Model**

Object detection has been witnessing a rapid revolutionary change in the field of computer vision. Basically, it involves two tasks:

- Object localization: determine where objects are located in a given image. Specifically, object detection model will use rectangular bounding boxes to locate all the detected objects in the image.
- Object classification: determine which category each detected object belongs to. Specifically, for each detected object, object detection model will calculate a probability for each target category, indicating how likelihood this detected object be- longs to this specific category.

Currently, there are many popular object detection models (e.g., SSD [12], RetinaNet [13], Faster R-CNN [14], Mask R-CNN [15]) available in different deep learning frameworks. All these models have one common part called feature extractor, which focuses on calculating high quality features for object detection tasks. In order to deal with the problem of limited resources on the mobile device (e.g., small storage space and limited battery power), researchers have proposed some efficient feature extraction network architecture specifically tailored for mobile and resource constrained environments, such as MobileNets [16], MobileNetV2 [17]. In this work, we mainly focus on the SSD with MobileNetV2 (we call it SSD-Mobilenet-V2). SSD (Single Shot MultiBox Detector) is a popular algorithm in object detection. Mobilenet-V2 is a

convolution neural network used to produce high-level features which can be used as a backbone feature extractor for SSD. It is small, low-latency, low-power and parameterized to meet the resource constraints of a variety of use cases. Specifically, it can be run efficiently on mobile devices with TensorFlow-Lite. SSD-Mobilenet-V2 combines the advantages of the two models, enabling it to efficiently perform target detection tasks on mobile devices.

Transferring well-trained object detection models on one dataset to another new dataset is a common approach called transfer learning. It has several benefits, but the main advantages are saving training time, getting better performance, and not needing a lot of data. Google has trained one SSD-MobileNet-V2 object detection model using COCO dataset (Microsoft. https://cocodataset.org.), which has 90 different categories. This pre-trained model can be used as a good starting point for our OPTIMA-dx detection model to help us save time and get better performance. Specifically, since our project only has 2 categories (positive and negative), we modified some final layers to make it produce only 2 outputs corresponding to our own 2 categories.

- **Dataset**

To train our model, we created one OPTIMA-dx image dataset using P51™ Molecular Fluorescence Viewer (minipcr. https://www.minipcr.com/product/p51- molecular-glow-lab). Specifically, we used cameras on different mobile devices to take pictures of the tubes shown on this device. Currently, we have 391 pictures directly taken from mobile device cameras. When taking these pictures, due to some random lighting, angles, jitter and other issues, some pictures are distorted and blurred, which are not suitable for training and testing our model, and need to be deleted. We further augmented these images by randomly modifying the brightness, contrast, color and sharpness, which generated a new set of images. We then make each image square by cropping to make sure each tube in each image can maintain a normal aspect ratio during model training and testing. Next, we randomly divided the images that meet the requirement into a training set (540 pictures) and a test set (66 pictures). Finally, the ground-truth bounding boxes for each picture in training and test set were created by LabelImg (tzutalin. https://github.com/tzutalin/labelimg).

- **Model training**

In order to transfer visual knowledge learned from the large-scale generic dataset COCO to our model, we initialized our model using Google's pre-trained SSD- Mobilenet-V2. We then trained the model using our own training set. Specifically, we trained it on one GeForce GTX 1080 Ti GPU with batch size 10. RMSprop optimizer was used with initial learning rate as 0.004. We trained a total of 35000 steps/batches and saved one checkpoint every 10 minutes. Then we can do test using all these saved checkpoints.

- **Model testing**

The most common metric used to evaluate the performance of object detection model is the mAP (mean average precision) [16, 17]. In our testing process, we calculate mAP on the test set for each saved check point, and finally take the checkpoint with the largest mAP. Currently, the best mAP is 97.6%.

- **Android App development**

We convert the selected checkpoint into a TensorFlow Lite model using the model converter tool (Google. https://www.tensorflow.org/lite/convert) provided by TensorFlow. Then we can deploy and run this TensorFlow Lite model on mobile device. For each input image, the TensorFlow Lite model can output 3 different kind of information: 1) the location of the bounding box for each detected tube, containing four coordinate values on the image plane: left, top, right and bottom. 2) Category for each detected tube and its value is either positive or negative. 3) One confidence score indicating the probability that the model thinks one tube belongs to the category. Finally, we draw the bounding boxes, categories and confidence scores over the input image and display the final image on screen.

The code for the smart phone app and to download the app is available at: https://hi-zhengcheng.github.io/optima-dx

## References


1. Couvin, D., et al., *CRISPRCasFinder, an update of CRISRFinder, includes a portable version, enhanced performance and integrates search for Cas proteins.* Nucleic Acids Res, 2018. **46**(W1): p. W246-W251.
2. Biswas, A., et al., *CRISPRDetect: A flexible algorithm to define CRISPR arrays.* BMC Genomics, 2016. **17**: p. 356.
3. Kellner, M.J., et al., *SHERLOCK: nucleic acid detection with CRISPR nucleases.* Nat Protoc, 2019. **14**(10): p. 2986-3012.
4. East-Seletsky, A., et al., *RNA Targeting by Functionally Orthogonal Type VI-A CRISPR-Cas Enzymes.* Mol Cell, 2017. **66**(3): p. 373-383 e3.
5. Joung, J., et al., *Detection of SARS-CoV-2 with SHERLOCK One-Pot Testing.* N Engl J Med, 2020. **383**(15): p. 1492-1494.
6. Ramachandran, A. and J.G. Santiago, *CRISPR Enzyme Kinetics for Molecular Diagnostics.* Anal Chem, 2021. **93**(20): p. 7456-7464.
7. Mahas, A., et al., *LAMP-Coupled CRISPR-Cas12a Module for Rapid and Sensitive Detection of Plant DNA Viruses.* Viruses, 2021. **13**(3).
8. Abadi, M., et al., *Tensorflow: Large-scale machine learning on heterogeneous distributed systems.* arXiv preprint arXiv:1603.04467, 2016.
9. Chen, T., et al., *Mxnet: A flexible and efficient machine learning library for heterogeneous distributed systems.* arXiv preprint arXiv:1512.01274, 2015.
10. Jia, Y., et al. *Caffe: Convolutional architecture for fast feature embedding*. in *Proceedings of the 22nd ACM international conference on Multimedia*. 2014.
11. Paszke, A., et al., *Pytorch: An imperative style, high-performance deep learning library.* arXiv preprint arXiv:1912.01703, 2019.
12. Liu, W., et al. *Ssd: Single shot multibox detector*. in *European conference on computer vision*. 2016. Springer.
13. Lin, T.-Y., et al. *Focal loss for dense object detection*. in *Proceedings of the IEEE international conference on computer vision*. 2017.
14. Ren, S., et al., *Faster r-cnn: Towards real-time object detection with region proposal networks.* arXiv preprint arXiv:1506.01497, 2015.
15. He, K., et al. *Mask r-cnn*. in *Proceedings of the IEEE international conference on computer vision*. 2017.
16. Howard, A.G., et al., *Mobilenets: Efficient convolutional neural networks for mobile vision applications.* arXiv preprint arXiv:1704.04861, 2017.
17. Sandler, M., et al. *Mobilenetv2: Inverted residuals and linear bottlenecks*. in *Proceedings of the IEEE conference on computer vision and pattern recognition*. 2018.
